# Axonal neuregulin 1 is a rate limiting but not essential factor for nerve remyelination

**DOI:** 10.1093/brain/awt148

**Published:** 2013-06-24

**Authors:** Florence R. Fricker, Ana Antunes-Martins, Jorge Galino, Remi Paramsothy, Federica La Russa, James Perkins, Rebecca Goldberg, Jack Brelstaff, Ning Zhu, Stephen B. McMahon, Christine Orengo, Alistair N. Garratt, Carmen Birchmeier, David L. H. Bennett

**Affiliations:** 1 The Nuffield Department of Clinical Neurosciences, University of Oxford, John Radcliffe Hospital, Oxford, OX3 9DU UK; 2 Wolfson CARD, King's College London, Guy’s Campus, London, SE1 1UL, UK; 3 Institute of Structural and Molecular Biology, University College of London, Gower Street, London WC1E 6BT, UK; 4 Max Delbrueck Centre for Molecular Medicine, Robert-Roessle-Strasse 10, 13092 Berlin, Germany; 5 Charité Universitätsmedizin Berlin, Charitéplatz 1, 10117 Berlin, Germany

**Keywords:** injury, Nrg1, regeneration, remyelination, Schwann

## Abstract

Neuregulin 1 acts as an axonal signal that regulates multiple aspects of Schwann cell development including the survival and migration of Schwann cell precursors, the ensheathment of axons and subsequent elaboration of the myelin sheath. To examine the role of this factor in remyelination and repair following nerve injury, we ablated neuregulin 1 in the adult nervous system using a tamoxifen inducible Cre recombinase transgenic mouse system. The loss of neuregulin 1 impaired remyelination after nerve crush, but did not affect Schwann cell proliferation associated with Wallerian degeneration or axon regeneration or the clearance of myelin debris by macrophages. Myelination changes were most marked at 10 days after injury but still apparent at 2 months post-crush. Transcriptional analysis demonstrated reduced expression of myelin-related genes during nerve repair in animals lacking neuregulin 1. We also studied repair over a prolonged time course in a more severe injury model, sciatic nerve transection and reanastamosis. In the neuregulin 1 mutant mice, remyelination was again impaired 2 months after nerve transection and reanastamosis. However, by 3 months post-injury axons lacking neuregulin 1 were effectively remyelinated and virtually indistinguishable from control. Neuregulin 1 signalling is therefore an important factor in nerve repair regulating the rate of remyelination and functional recovery at early phases following injury. In contrast to development, however, the determination of myelination fate following nerve injury is not dependent on axonal neuregulin 1 expression. In the early phase following injury, axonal neuregulin 1 therefore promotes nerve repair, but at late stages other signalling pathways appear to compensate.

## Introduction

Neuregulin 1 (*Nrg1*) plays key roles in the development of the PNS and controls the survival of Schwann cell precursors, Schwann cell motility, axon ensheathment and myelination (Birchmeier and Nave, 2008). Among the many isoforms of NRG1 that exist, it is the type III NRG1 isoforms (and principally Nrg1TIII β1a) that are expressed on the axolemma and that activate receptors on Schwann cells (ERBB2/ERBB3 heteromers) to drive Schwann cell development, axon ensheathment and myelin thickness (Garratt *et al.*, 2000; Michailov *et al.*, 2004; Taveggia *et al.*, 2005; Chen *et al.*, 2006; Hu *et al.*, 2006; Willem *et al.*, 2006).

The roles of NRG1 in adulthood have been more challenging to elucidate owing to the embryonic lethality of null mutations. Myelin integrity is unchanged following ablation of ERBB2 receptors in adulthood (Atanasoski *et al.*, 2006) and, in agreement, juxtacrine NRG1 signalling is dispensable for the maintenance of the myelin sheath in adult peripheral nerves (Fricker *et al.*, 2011). Traumatic nerve injury is followed by a stereotyped series of events in animal models and in humans. In an early phase of Wallerian degeneration, axons degenerate (Coleman and Freeman, 2010) which is followed by de-differentiation of Schwann cells. These Schwann cells proliferate (Bradley and Asbury, 1970), downregulate myelin gene expression and adopt a repair phenotype (Jessen and Mirsky, 2008; Arthur-Farraj *et al.*, 2012). Subsequently, axons begin to regenerate, Schwann cells differentiate and remyelinate and targets are reinnervated (Chen *et al.*, 2007). Although similarities between the initial development and the repair of myelin are apparent, key differences exist (Fancy *et al.*, 2011), and for instance, remyelinated axons have shorter internodes and thinner myelin sheaths. Traumatic nerve injury provides a model to study these events over a defined time course, but the understanding of axoglial signalling processes during repair in the adult is also relevant to understanding peripheral neuropathies caused by immune, metabolic and genetic dysfunctions (Taveggia *et al.*, 2010). Thus, an understanding of the molecular mechanisms may provide therapeutic strategies to intervene in order to promote effective repair. Given its important developmental role, NRG1 is a candidate for providing neuronal signals during repair. Following injury, expression of NRG1 isoforms and ERBB2 and ERBB3 receptors change dramatically in the peripheral nerve. Levels of Ig-containing isoforms of NRG1 (types I, II, IV, V and VI) and ERBB2 and ERBB3 receptors in Schwann cells significantly increase in the distal nerve following injury. This is first detected 3 days post-injury and is sustained for at least 30 days (Cohen *et al.*, 1992; Carroll *et al.*, 1997; Kwon *et al.*, 1997; Fricker and Bennett, 2011). In contrast, neuronal NRG1 type III levels initially decrease but increase again between 7 and 10 days post-injury when targets are reinnervated (Bermingham-McDonogh *et al.*, 1997; Carroll *et al.*, 1997). By ablation of NRG1 in a small subpopulation of peripheral neurons, we have previously shown that axonally-derived NRG1 is required for remyelination at early time points following peripheral nerve injury (Fricker *et al.*, 2011). A role of endogenous NRG1 in the functional recovery following peripheral nerve injury has not been analysed, nor has the mechanism by which NRG1 may promote peripheral nerve repair been investigated.

In this study, we employ a transgenic mouse model to ablate all isoforms of NRG1 in nervous tissue in adulthood by the use of a tamoxifen inducible Cre recombinase that is expressed ubiquitously (Hayashi and McMahon, 2002; Guy *et al.*, 2007). We confirm that NRG1 signalling is not required for the maintenance of the uninjured peripheral nerve structure. NRG1 is dispensable for Schwann cell proliferation associated with either axon degeneration or regeneration, but promotes multiple aspects of the reparative response including re-myelination and re-innervation of the neuromuscular junction and of the epidermis. Interestingly, eventually remyelination does occur with efficient discrimination between small and large diameter axons, indicating that after injury in adulthood the myelination fate is determined independently of axonal NRG1.

## Materials and methods

### Animals

All work carried out conformed to UK Home Office legislation (Scientific Procedures Act 1986). CAG-Cre-ER^TM^; Nrg1*^fl/fl^* mice were bred by crossing Nrg1*^fl/fl^* mice with CAG-Cre-ER^TM^ mice [JAX(r) Mice 004682]; both colonies were on a C57BL/6 background (Hayashi and McMahon, 2002). The generation and genotyping of mutant mice with floxed alleles of *Nrg1* (Nrg1*^fl/fl^*) mice has previously been described (Yang *et al.* 2001; Brinkmann *et al.* 2008; Young *et al.* 2008). These mice are null for α-isoforms of NRG1 in the absence of Cre recombination as they carry a premature stop codon in exon 7, which encodes the α-EGF domain (Li *et al.*, 2002). The loxP sites flank exons 7–9, and exon 8 encodes the β-EGF domain. Cre recombination therefore results in ablation of all remaining β isoforms. To detect the CAG-Cre-ER^TM^ construct, PCR of genomic DNA was carried out (see Supplementary material for primers). In CAG-Cre-ER^TM^ mice, a tamoxifen inducible form of Cre recombinase is expressed ubiquitously driven by a chimeric promoter constructed from a cytomegalovirus intermediate-early enhancer and a chicken β actin promoter/enhancer (Hayashi and McMahon, 2002). To evaluate CAG-Cre-ER^TM^ expression and induction, CAG-Cre-ER^TM^ animals were crossed with Rosa reporter mice (R26R), resulting in CAG-Cre-ER^TM^;R26R mice. The generation and genotyping of Nrg1*^fl/fl^*;Nav1.8-Cre mice has been described previously (Fricker *et al.*, 2009).

Conditional NRG1 (conNrg1) mutant mice were generated by administering tamoxifen (Sigma T5648, 0.25 mg/g body weight in corn oil) by oral gavage for five consecutive days to 10-week-old CAG-Cre-ER^TM^; Nrg1*^fl/fl^* mice. After this treatment, animals were left for 4 weeks before surgery. Two types of control animals were used for comparison: Vehicle controls, which were CAG-Cre-ER^TM^;Nrg1*^fl/fl^* littermates treated with corn oil alone and tamoxifen control, which were tamoxifen treated CAG-Cre-ER^TM^; Nrg1*^+/+^*. Wherever possible, we included equal numbers of animals of each gender in each experimental group.

### Surgery

One month post-tamoxifen/vehicle treatment, nerve crush surgery, nerve transection or nerve transection followed by repair was carried out (see Supplementary Fig. 1 for summary of the different models). For anastamosis experiments, the left tibial nerve was double ligated with 5-0 prolene sutures as close to the branching site of the sciatic nerve as possible, transected between the ligatures, and the wound was then closed. A cohort of animals underwent a repair 2 weeks after the initial injury; the common peroneal nerve was ligated close to the branching site of the sciatic nerve, and then transected above the ligature. The distal ligature on the tibial nerve was trimmed and the nerve stump and the proximal freshly cut common peroneal nerve branch were manipulated into close apposition. The two nerves were co-apted using fibrin glue, the wound was closed and axons were allowed to regenerate into the denervated nerve stump for 10 days. For nerve transection and reanastomosis the sciatic nerve was immediately co-apted using fibrin glue following the transection. For nerve crushes, the left sciatic nerve was exposed and crushed twice, in two different directions 30 s each time, with fine forceps. The crush site was labelled with lamp black. In all cases the lesion site was kept a constant 43 mm from the tip of the third digit and the wound was closed with 6-0 sutures and disinfected. To assess Schwann cell proliferation, intraperitoneal injections of 100 mg/kg 5-Bromo-2′-deoxyuridine (BrdU) (Sigma 858811) dissolved in filtered PBS were given daily for the 3 days before animals were euthanized.

### Western blotting

Tissues were homogenized in NP40 lysis buffer and the lysates were spun at 13 000 rpm for 15 min and the protein concentration of supernatant was determined using a BCA Protein Assay kit (Thermo Scientific, 23 227). Protein (50-100 µg) was mixed with SDS gel sample buffer and electrophoresed on 8% SDS-polyacrylamide gels, and transferred to nitrocellulose membranes, blocked in 10% w/v milk and immunoblotted with antibodies against NRG1 [Neuregulin-1α/β1/2 (C-20) SantaCruz SC-348] at a dilution of 1:500. Secondary antibodies were anti-rabbit IgG horseradish peroxidase linked (GE Healthcare NA9340V;1: 10 000). ECL prime western blotting detection system (GE Healthcare) was used to visualize the immunoreactive band on chemiluminescence film (GE Healthcare). Membranes were stripped and immunoblotted with anti-beta-actin (Clone AC-15, Sigma A1978; 1:5000). Secondary antibodies were anti-mouse IgG horseradish peroxidase linked (GE Healthcare NA931V; 1:10 000). The intensity of specific bands was quantified using Quantity One® software (Bio-Rad). The same size square was drawn around each band to measure intensity and the background was subtracted. Each band detected by the anti-NRG1 antibody [Neuregulin-1α/β1/2 (C-20) SantaCruz SC-348] was normalized against the loading control beta-actin.

### Histology

Animals were deeply anaesthetized with pentobarbitone and transcardially perfused with 5 ml saline followed by 25 ml paraformaldehyde (4% w/v in 0.1 M phosphate buffer). All tissue taken was post-fixed in paraformaldehyde (4% w/v in 0.1 M phosphate buffer), mounted in O.C.T. embedding compound on dry ice and stored at −80°C. Tissues were sectioned on a cryostat at the following thicknesses: tibial nerve and sciatic nerve at 10 μm, lumbar spinal cord 20 µm, skin 14 μm, onto SuperFrost Ultra Plus® slides. Longitudinal sections of gastrocnemius were cut on a freezing microtome at 100 μm into a 24 well plate and stored in PBS containing 0.1% sodium azide.

#### X-gal staining

For 5-bromo-4-chloro-3-indolyl-D-galactoside (X-gal) staining of tissue sections, the sections were washed and then incubated overnight at 37°C in darkness covered with 3 mM potassium ferrocyanide, 3 mM potassium ferricyanide, 1 mM MgCl_2_, X-gal 1 mg/ml in 0.1 M phosphate buffer. Slides were washed with PBS and counterstained with Nuclear Fast Red. Sections were then dehydrated by washing in an ethanol series. Slides were submerged in xylene and covered with Distrene, Plasticizer, Xylene (DPX) mounting medium.

#### Immunohistological analysis

The following antibodies were used: rabbit S100 polyclonal antibody (Dako Z0311, 1:300), rat anti-BrdU (Abcam ab74545, 1:300), α-bungarotoxin Alexa Fluor® 647 conjugate (Invitrogen B-35450, 1:1000), mouse anti-2H3 (Developmental Studies Hybridoma Bank; 1:200) against 165 kDa neurofilament protein, monoclonal mouse anti-SV2 (Developmental Studies Hybridoma Bank 1:200), rat monoclonal antibody to myelin basic protein (Abcam 7349-2, 1:200), polyclonal rabbit PGP9.5 (Ultraclone 1:800), rabbit anti-IBA1 (WAKO 019-19741 1:1000) and rabbit Neuregulin-1α/β1/2 (C-20) (SantaCruz SC-348, 1:100). Secondary antibodies used: anti-rabbit-Cy3 (Stratech Scientific Ltd. 711-166-152, 1:500), Alexa Fluor® 488 goat anti-rat (Invitrogen A11006, 1:1000), donkey anti-mouse FITC (Stratech 715-095-150, 1:200), and Alexa Fluor® 546 goat anti-rat (Invitrogen A11051, 1:1000). For all stainings, sections were washed in PBS and blocked using 10% normal donkey serum (Chemicon S30). Primary antibodies were incubated overnight and secondary antibodies were incubated for 3 h, both at room temperature. All reagents were diluted in PBS containing 0.2% Triton™ X-100 and 0.1% sodium azide. For immunohistological analysis of BrdU, sciatic nerve sections were incubated in 1 M HCl at 37°C for 30 min before the normal protocol was applied.

### Image analysis

Immunofluorescence was visualised using a Zeiss Imager.Z1 microscope or a confocal Zeiss LSM 700 laser scanning microscope. Photographs were taken using the AxioCam and AxioVision LE Rel. 4.2 or the LSM Image Browser software for image analysis. BrdU and IBA1 positive cell quantification was performed by taking photomicrographs of three sections from different levels within the nerve per animal, and three to four animals per genotype were analysed. Two adjacent ×40 objective pictures using a *Z*-stack throughout the section were taken per section 2–3 mm from the injury site in the ipsilateral side, and approximately on the same level in the contralateral side. In the case of the BrdU analysis the percentage of nuclei [determined by 4',6-diamidino-2-phenylindole (DAPI) labelling] in S100 positive cells (i.e. Schwann cells) that were also BrdU-positive was determined. For MBP analysis pictures were taken as above; however, the exposure time was kept constant and the intensity of staining was measured using ImageJ software. Epidermal fibres were counted at a ×40 magnification on the microscope as described (Lauria *et al.*, 2005). Reinnervation of the gastrocnemius muscle was quantified by taking a complete mosaic picture of two whole sections of muscle per animal at ×20 magnification with a *Z*-stack through the entire thickness of the muscle on the confocal Zeiss LSM 700 laser scanning microscope. The total number of neuromuscular junctions, and the number that were innervated by axons were counted using Zeiss Zen software. In all cases, the person performing the analysis was blinded to genotype and to the presence of an injury.

### Electron microscopy

Sciatic nerves were dissected and a section 2–3 mm distal to the crush site on the injured side and an equivalent level on the uninjured side was processed for electron microscopy. Nerves were post-fixed in 4% paraformaldehyde, 3% glutaraldehyde in 0.1 M phosphate buffer at 4°C overnight, and processed as previously described (Fricker *et al.*, 2009). Ultrathin and semithin sections were cut and stained by the Centre for Ultrastructural Imaging Kings College, London. Semithin sections were analysed for numbers of macrophages and myelin ovoids. Macrophages were identified as cells containing foamy lipid bodies, and myelin ovoids as individual swirls of unravelling myelin. All macrophages and myelin ovoids were counted from an entire cross section of the sciatic nerve for each animal. Grids were viewed on a Hitachi H7600 transmission electron microscope. For analysis, photographs of randomly chosen entire grid squares were taken, the area of which totalled at least 20% of the total area of the cross section of the sciatic nerve. These photographs were taken at a magnification of ×8000 and then merged to construct montages of the grid square (∼100 pictures per grid square). The number of myelinated and unmyelinated axons as well as Schwann cell nuclei were counted from these montages of grid squares and normalized to the total area of the nerve. To calculate G-ratios, 25 individual pictures at ×8000 magnification were randomly picked using randomly generated numbers. All myelinated axons within each picture were measured to calculate the G-ratio and all unmyelinated axons in each picture were measured for axon diameter, using AxioVision LE Rel. 4.2 software. The examiner was blind to genotype.

### Sciatic functional index

Hind paws were inked and mice ran along a catwalk leaving their paw prints on the paper. Paw prints used for measuring were chosen based on clarity and consistency and a succession of 3-4 prints at a point at which the mouse was walking at a moderate pace. Both uninjured and injured paw prints were measured. Measurements were: toe spread, first to fifth toes; intermediate toe spread, second to fourth toes; and print length end of the third toe to the bottom of the hind pad. These measurements were then used to calculate the sciatic functional index (Bain *et al.*, 1989; Inserra *et al.*, 1998; Monte-Raso *et al.*, 2008).

### Neurophysiology

For recording of compound nerve action potentials, the animal was terminally anaesthetized and the sciatic nerve and its tibial branch were dissected from the level of the sciatic notch to the ankle. The nerve was washed in Hank’s buffered saline solution and freed from debris. The nerve was then placed in a chamber with three compartments that were sealed with Vaseline. One side compartment was used for stimulation, in which the sciatic nerve was laid over bipolar stimulating electrodes and filled with mineral oil. The crush site had been marked with carbon and was clearly visible; stimulation was performed just proximal to the injury site so that recordings represented function in the regenerated axons. The nerve was then passed through to a central compartment which was filled with Hank’s buffered saline solution. The final compartment containing the distal tibial nerve was filled with mineral oil and the nerve was laid over bipolar silver wire recording electrodes, which were 1 mm apart. Recordings were performed at room temperature, the nerve was crushed between the recording electrodes to ensure monophasic recording. Square wave current stimulation pulses of duration 100 µs were given at a frequency of 1 Hz. Evoked activity was recorded and threshold for generation of a compound nerve action potential was determined; recordings were made when stimulating at twice threshold and 32 recording sweeps were recorded and averaged using Powerlab software (ADI instruments). The distance between stimulating and recording electrodes was measured in order to calculate conduction velocity.

### Genome-wide expression profiling and real-time quantitative polymerase chain reaction analysis

Microarray analysis was performed on sciatic nerve tissue (after perineurium removal) of conNrg1 mutant and vehicle control mice at different time points after sciatic nerve crush: 10 days, uninjured/contralateral, (conNrg1 mutant; *n = *4; vehicle control *n = *5) and injured nerves (conNrg1 mutant *n = *5; vehicle control *n = *5); and 28 days ipsilateral only (conNrg1 mutant *n = *4; vehicle control *n = *4). Total RNA was extracted using the miRNeasy mini kit (Qiagen), according to the manufacturer’s instructions, including treatment with RNase-free DNase (Qiagen). Complementary DNA was amplified from 20 ng of total RNA using the Ovation® Pico WTA system with Ribo-SPIA technology (NuGEN) and was further processed for hybridization to Affymetrix mouse gene 1.0 ST arrays (Affymetrix) according to manufacturer’s instructions. RNA processing and hybridization were performed by UCL genomics. Background adjustment, normalization and summarization of probes to the transcript level were performed using the Robust Multichip Average (RMA) method (Irizarry *et al.*, 2003). Differential expression between conNrg1 mutant and vehicle control animals was calculated for each time point using the Linear Models for Microarray data (LIMMA) package (Smyth, 2004). A False Discovery Rate (FDR) *P*-value cut-off of <0.1 was used throughout the analysis. Transcripts differentially expressed at the 10 day time point were used for clustering across all experimental groups and functional enrichment analysis. Clustering was performed using BioLayout Express3D (Theocharidis *et al.*, 2009), using a Pearson Correlation threshold = 0.7 and default settings. Functional enrichment analysis was performed using the ‘core analysis’ option of the Ingenuity Pathway Analysis software (Ingenuity Systems) using default settings. Enrichments for ‘biological functions’ are represented as ratios (number of genes in the dataset/ number of genes in database) and respective *P*-values given.

For real-time quantitative PCR analysis, complementary DNA was reverse transcribed from total RNA using random primers (Promega) and SuperScript® III Reverse Transcriptase (Invitrogen). Real-time quantitative PCR for *Nrg1*, *Mbp* and *Mpz* was performed using the LC FS DNA MasterPLUS SG (Roche) with final primer concentrations of 1 µM, in a Rotor Gene 6000 (Corbett Research) instrument. Relative quantification to a reference gene (*GAPDH*) was performed using the ΔΔCt method (see Supplementary material for primer pairs). *Nrg1* primers were designed with Primer3 software (http://frodo.wi.mit.edu/) and submitted for BLAST analysis to ensure specificity.

### Statistics

The Students *t*-test was used for comparison of two groups, one-way ANOVA or two-way ANOVA using the Tukey *post hoc* test for more than two groups. Results are reported as mean values ± SEM. Cumulative frequencies were compared statistically using the Kolmogorov-Smirnov test.

## Results

### Efficient inducible ablation of NRG1 in adulthood

**Figure 1:**
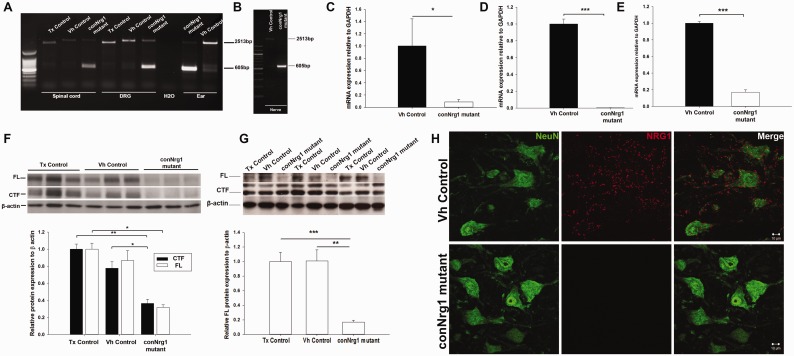
NRG1 is efficiently ablated following tamoxifen treatment. (**A–B**) PCR of genomic DNA extracted from spinal cord, dorsal root ganglion, ear punches and sciatic nerve (**B**), using primers to detect the recombined floxed allele with a size of 605 bp. The product amplified from the unrecombined flox allele can also be detected running at 2513 bp; note that in animals with a wild-type *Nrg1* allele, i.e. lacking loxP sites (tamoxifen control mice), the product amplified from the *Nrg1* allele can be seen to run at a lower size of 2219 bp. The 605 bp band can be seen only in the tamoxifen treated CAG-Cre-ERTM;Nrg1*^fl/fl^* mice (conNrg1 mutant) whereas the unrecombined allele is seen in vehicle-treated CAG-Cre-ERTM;Nrg1*^fl/fl^* mice (vehicle control) or tamoxifen treated CAG-Cre-ERTM;Nrg1*^+/+^*mice (tamoxifen control). (**C*–*E**) Quantitative real-time PCR with primers designed against the βEGF domain, relative to the expression of *GAPDH*. A significant decrease in messenger RNA expression is seen in sciatic nerve a reduction of 91% (**C**), lumbar dorsal root ganglions a reduction of 99.7% (**D**) and lumbar spinal cord, a reduction of 83% (**E**) *n* = 4, Student’s *t*-test***.*** (**F–G**) Western blot analysis of lumbar spinal cord (**F**) and sciatic nerve (**G**), the membrane was probed with an NRG1 antibody that recognizes the ‘a-tail’ C terminal epitope. The 135 kDa band represents full length NRG1 type III pro-protein and the 60 kDa band represents a cleaved terminal fragment (CTF) (which does not contain the EGF domain) of the NRG1 protein. β-actin was used as a loading control. A clear reduction of the full length band can be seen in both spinal cord and nerve but the cleaved terminal form can still be detected in nerve. (**H**) Photomicrographs of the ventral horn of the spinal cord at the level of L5 from the vehicle control and conNrg1 mutants, motor neurons are identified by their characteristic morphology and the NeuN labelling, NRG1 labelling can be seen as punctate staining in vehicle spinal cords but is no longer present in conNrg1mutants. Scale bars = 10 μm. All the tissue in this figure was taken from uninjured animals (14 weeks of age) that had been dosed with either vehicle or tamoxifen 4 weeks previously. Tx = tamoxifen; Vh = vehicle. *P < 0.05, **P < 0.005 and ***P < 0.001, one-way ANOVA *post hoc* Tukey.

After tamoxifen treatment, adult CAG-Cre-ER^TM^;R26R reporter mice showed clear induction of Cre recombinase activity in motor neurons in the lumbar spinal cord, with 86.8 ± 3.9% motor neurons positive for the β-galactosidase blue reaction product 5,5'-dibromo-4,4'-dichloro-indigo. In sensory neurons in the dorsal root ganglion 94.8 ± 1.1% neuronal cell bodies were positive and clear induction was seen in cells of the sciatic nerve (Supplementary Fig. 2). CAG-Cre-ER^TM^ mice were crossed with mice homozygous for the conditional *Nrg1* allele (Nrg1*^fl/fl^*), which have a premature stop codon in the αEGF domain. They are therefore functionally null for αNRG1 (which is not required for nervous system development) and Cre-mediated recombination within the βEGF domain results in functional ablation of all NRG1 isoforms (Meyer and Birchmeier, 1995; Yang *et al.*, 2001; Li *et al.*, 2002). In CAG-Cre-ER^TM^;Nrg1*^fl/fl^* mice (conNrg1 mutant), PCR demonstrated recombination of the floxed allele after tamoxifen treatment, but recombination was not detected in vehicle treated or tamoxifen control mice (Fig. 1A and B). A large decrease in *Nrg1* expression detected by primers flanking the region encoding the βEGF domain was seen in conNrg1 but not control mice (Fig. 1C–E). Further evidence of ablation of NRG1 was provided by western blot analysis using an antibody that recognizes the most abundant a-tail isoforms (Fig. 1F and G). After tamoxifen treatment, expression of NRG1 was no longer detected in the cell bodies of motor neurons in lumbar spinal cord by immunohistological analysis (Fig. 1H).

### NRG1 does not regulate Schwann cell proliferation following peripheral nerve injury

**Figure 2:**
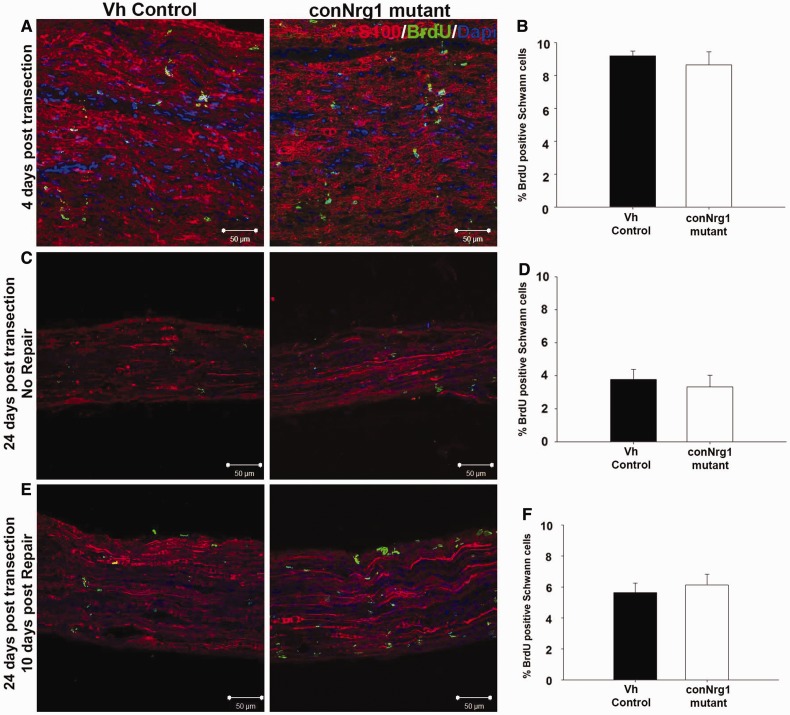
Nrg1 signalling is dispensable for Schwann cell proliferation following peripheral nerve injury. (**A**) Photomicrographs of longitudinal sciatic nerve 4 days post transection, (**B**) Percentage of Schwann cells positive for BrdU 4 days post transection. (**C**) Photomicrographs of longitudinal sections of tibial nerve 24 days post transection (without undergoing a repair; see Supplementary Fig. 1C). (**D**) Percentage of Schwann cells positive for BrdU 24 days post transection. (**E**) Photomicrographs of longitudinal sections of tibial nerve 10 days post repair, reflecting Schwann cell proliferation associated with axonal re-contact (repair following 2 weeks denervation; see Supplementary Fig. 1C). (**F**) Percentage of Schwann cells positive for BrdU 10 days post repair. Schwann cells are labelled with S100 (red), proliferating cells with BrdU (green) and nuclei with DAPI (blue). Scale bars = 50 μm, *n = *5. Vh = vehicle.

One of the very first consequences of peripheral nerve injury is a wave of Schwann cell proliferation due to the loss of axonal contact that peaks at 3 days post injury (Bradley and Asbury, 1970; Stoll and Muller, 1999; Griffin and Thompson, 2008). A second wave of Schwann cell proliferation is subsequently induced when Schwann cells re-contact regenerating axons in the distal nerve segment (Pellegrino and Spencer, 1985). To examine whether NRG1 signalling plays a role in Schwann cell proliferation associated with axon degeneration, conNrg1 mutant and control mice underwent a sciatic nerve transection and ligation to prevent reinnervation. Four days later, tissue was harvested from mice. A significant increase in Schwann cell proliferation at 4 days post-injury was seen compared with uninjured levels using BrdU incorporation, which diminished over time but was still apparent at 24 days post-injury in a denervated tibial nerve stump (Fig. 2A–D). We detected no difference in Schwann cell proliferation between the control and conNrg1 mutant animals at either time post injury. To distinguish between the initial wave of Schwann cell proliferation associated with axon degeneration and the second wave of Schwann cell proliferation that occurs during repair, i.e. when axons re-contact Schwann cells, a delayed nerve repair model was utilized (Pellegrino and Spencer, 1985) (Supplementary Fig. 1). The tibial nerve was left to denervate for 2 weeks before a freshly cut common peroneal nerve was anastamosed to the tibial nerve stump distal of the lesion, which allows axons to re-contact denervated Schwann cells. Ten days after this, Schwann cell proliferation during repair was again examined (Fig. 2E and F). Schwann cell proliferation upon axonal re-contact was increased compared to that observed in a denervated stump without repair (Fig. 2D), but again we observed no difference between control and conNrg1 mutant mice. Following peripheral nerve injury, NRG1 signalling is therefore dispensable for Schwann cell proliferation after the initial axonal loss and during the ensuing axonal regeneration.

### NRG1 does not regulate macrophage recruitment or myelin clearance after peripheral nerve injury

**Figure 3:**
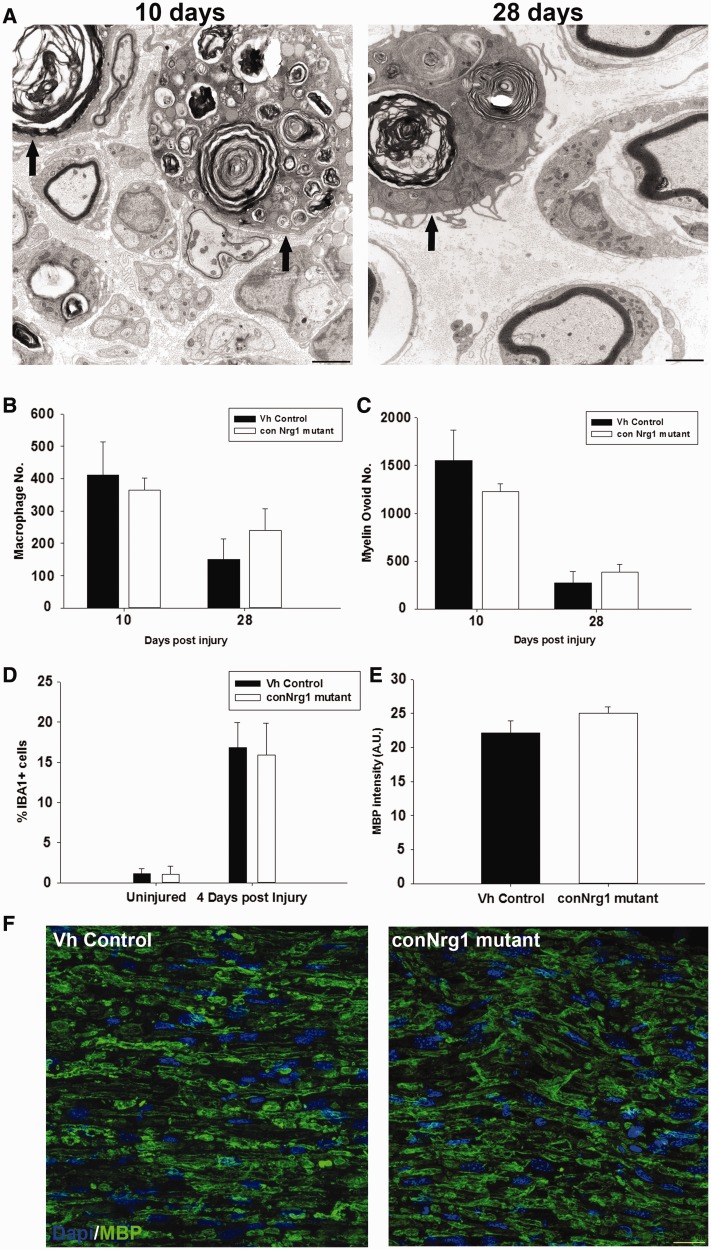
Nrg1 signalling is dispensable for macrophage recruitment and myelin clearance following peripheral nerve injury. (**A**) Electron micrographs of transverse sections of sciatic nerve 10 days and 28 days following sciatic nerve crush injury. Arrows identify macrophages containing myelin ovoids. Quantification of macrophage numbers (**B**), and myelin ovoid numbers (**C**), show that there is a decrease over time but the absence of NRG1 has no effect. (**D**) Quantification of the number of IBA1-positive cells in uninjured sciatic nerve and 4 days post-injury. (**E**) Quantification of MBP intensity 4 days post injury. (**F**) Representative photomicrographs of transverse sections of sciatic nerve 4 days post injury labelled with DAPI and MBP. Scale bar = 20 µm. Vh = vehicle.

An early event following peripheral nerve injury is the recruitment of macrophages and the dedifferentiation of Schwann cells. To examine whether NRG1 plays a role in the recruitment of macrophages and the subsequent removal of myelin debris, macrophages were counted 10 and 28 days following peripheral nerve crush (Fig. 3A and B), there was no change in the number of macrophages (Fig. 3B), nor in the number of myelin ovoids contained within them (Fig. 3C). In addition at the earlier time point of 4 days post-injury and in uninjured nerve there was no change in the number of cells that labelled positive for IBA1, a marker for macrophages (Fig. 3D). There was also no change in MBP immunostaining in sciatic nerve 4 days post-injury (Fig. 3E and F), indicating that there is no delay in myelin clearance following peripheral nerve injury in the absence of NRG1.

### NRG1 promotes remyelination and target reinnervation after peripheral nerve injury

Consistent with our previous findings and those of others, we did not observe any change in the morphology of the uninjured sciatic nerve in conNrg1 mutant animals, i.e. 12 weeks following tamoxifen treatment, the number of axons, G-ratio, unmyelinated axon morphology and gastrocnemius muscle innervation were unchanged (Supplementary Fig. 3). To assess the role of NRG1 in nerve repair, we employed a model of sciatic nerve crush or the more severe injury of sciatic nerve transection and reanastamosis (Supplementary Fig. 1A and B).

**Figure 4:**
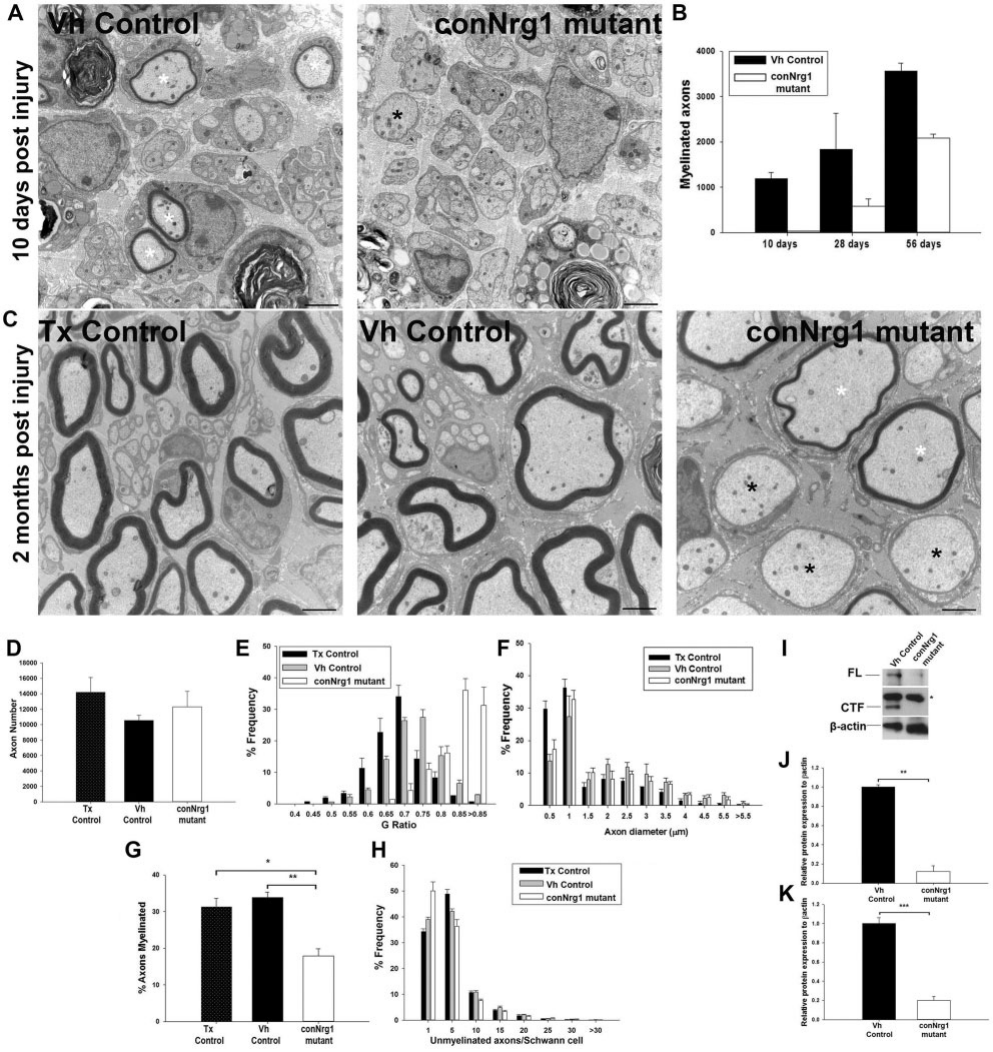
Nrg1 signalling promotes remyelination following peripheral nerve injury. (**A**) Electron micrographs of transverse sections of the sciatic nerve 10 days after sciatic nerve crush injury in vehicle control and conNrg1 mutant animals. In vehicle control animals most large diameter axons are undergoing remyelination (white asterisk) whereas in conNrg1 mutant animals large diameter axons are still without myelin (black asterisk). (**B**) The number of myelinated axons per sciatic nerve cross section at 10 days, 28 days and 2 months post-sciatic nerve crush. In conNrg1 mutant animals there are many fewer myelinated axons but this does increase over time *n = *3. (**C**) Electron micrographs of transverse sections of the sciatic nerve 2 months after sciatic nerve crush injury in vehicle control and conNrg1 mutant animals. In vehicle and tamoxifen control animals effective remyelination can be seen. In contrast, in conNrg1 mutant animals**,** frequently**,** axons with a diameter of >1 μm are ensheathed by a Schwann cell but have no myelin sheath (black asterisk), or remyelination has occurred but the myelin sheath is abnormally thin (white asterisk). Scale bars = 2 μm. (**D–H**) Analysis of 2 month post-injury nerve morphology. (**D**) Total axon number counts, the total number of axons present in the sciatic nerve is unchanged between groups following peripheral nerve injury. (**E**) Frequency distribution of G-ratios, there is a clear shift to higher G-ratios in the conNrg1 mutant animals (*P* < 0.001 Kolmogorov-Smirnov test). (**F**) Frequency distribution of axon diameters, axon diameters are unchanged. (**G**) The percentage of axons that have a myelin sheath 2 months following peripheral nerve injury is decreased in conNrg1 mutant animals compared with vehicle and tamoxifen control animals. *P < 0.05, **P < 0.005 one-way ANOVA *post hoc* Tukey *n* = 4. (**H**) Frequency distribution of unmyelinated axons associated with each Schwann cell in control vehicle and tamoxifen nerves 2 months post-sciatic nerve crush, *n* = 4. There is no change in the distribution frequencies between vehicle and tamoxifen animals, note the higher numbers of unmyelinated axons in a 1:1 relationship with a Schwann cell post injury in both groups. (**I**) Western blots of injured sciatic nerve 28 days post injury probed with an NRG1 antibody showing the full length (FL) and cleaved terminal form (CTF) forms of the NRG1 protein. The asterisk identifies a non-specific band. β-actin was used as a loading control. (**J**) Western blot analysis of the full length NRG1 form, which is reduced in tamoxifen animals (**P < 0.005 Student’s *t*-test) and (**K**) analysis of the cleaved terminal form NRG1 form also reduced in tamoxifen animals (***P < 0.001 Student’s *t*-test). *n* = 3. Tx = tamoxifen; Vh = vehicle.

Ten days following sciatic nerve crush, many axons 2 mm distal to the crush site were observed undergoing remyelination in control mice. In conNrg1 mutant mice, we noted many naked axons and only very occasionally axons had undergone remyelination (Fig. 4A). More myelinated axons were present at 28 days post-injury in conNrg1 mutant mice but the number of myelinated axons was still 61% less than in control mice (Fig. 4B). Two months following sciatic nerve crush, control animals (either vehicle or tamoxifen control animals) showed effective remyelination (Fig. 4C). Remyelination in conNrg1 mutants was still deficient at this stage, and in particular a significant proportion of large axons were ensheathed by Schwann cells but had failed to elaborate a myelin sheath (Fig. 4C and G). Those axons that were remyelinated had thinner myelin sheaths (Fig. 4C), reflected in a considerable (and significant) shift in frequency distribution to much larger G-ratios (Fig. 4E). There was a much smaller shift in frequency distribution towards a larger G-ratio in vehicle control compared with tamoxifen controls. Vehicle control and tamoxifen control are not littermates and this small difference could be due to either the absence of αNRG1 isoforms in the vehicle control or potentially an effect of tamoxifen itself (G-ratios mean ± SEM; 0.661 ± 0.0002 tamoxifen control, 0.705 ± 0.0046 vehicle control and 0.815 ± 0.0089 conNrg1 mutant, *P* < 0.001 conNrg1 versus vehicle control and *P* < 0.001 conNrg1 versus tamoxifen control, tamoxifen control versus vehicle control *P* = 0.009, one-way ANOVA *post hoc* Tukey). There was an increase in the average diameter of unmyelinated axons (axon diameters mean ± SEM; 0.56 ± 0.0097 µm tamoxifen control, 0.65 ± 0.008 µm vehicle control and 0.86 ± 0.056 µm conNrg1, *P* < 0.05). However the frequency distribution of axon diameters overall (Fig. 4F), nor the average diameter of myelinated axons did not change (axon diameters mean ± SEM; 2.37 ± 0.094 µm tamoxifen control, 2.60 ± 0.018 µm vehicle control and 2.88 ± 0.212 µm conNrg1). The total numbers of axons were similar in control and mutant mice (Fig. 4D) but the percentage that were myelinated was much reduced (Fig. 4G). We did not detect a change in the numbers of unmyelinated axons ensheathed per Schwann cell (Fig. 4H), nor did we see any structural abnormalities in Remak bundles at any time points in conNrg1 mutants. We confirmed reduced NRG1 protein levels at 28 days post-injury in the *Nrg1* mutant mice, as assessed by western blotting analysis of protein lysate of injured sciatic nerves, and both full length NRG1 and cleaved terminal form were detected at reduced levels (Fig. 4I–K).

**Figure 5:**
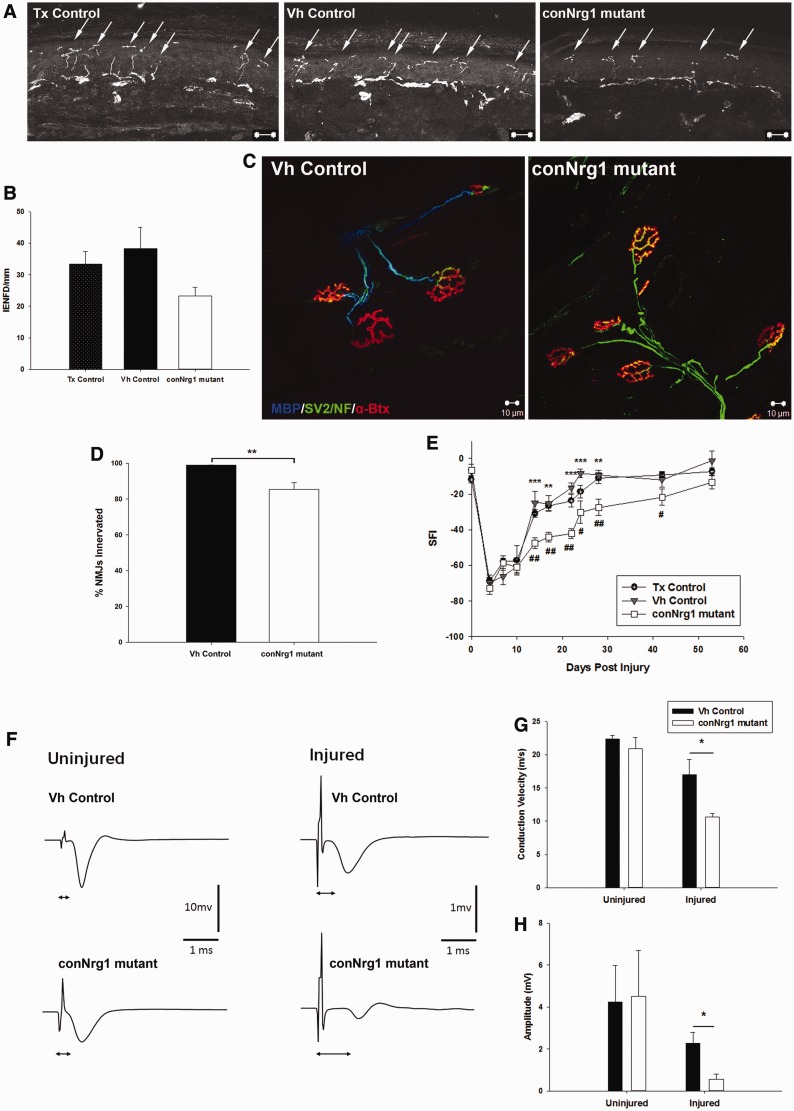
NRG1 promotes reinnervation of targets and functional recovery following peripheral nerve injury. (**A**) Photomicrographs of transverse sections of glabrous skin from the hind paw of skin 2 months post-injury, arrows identify individual epidermal fibres. (**B**) Quantification of epidermal innervation, intraepidermal nerve fibre (IENF)/mm, a trend to fewer fibres reinnervating the epidermis 2 months after injury in conNrg1 mutant animals is seen, although this does not reach significance. (**C**) Photomicrographs of neuromuscular junctions, the gastrocnemius is labelled for: MBP (blue), SV2 and neurofilament marker NF (green) and α-bungarotoxin (red). Neuromuscular junction morphology is normal 8 weeks following sciatic nerve crush in both vehicle control and conNrg1 groups. Note the lack of myelin staining following injury in conNrg1 mutant animals. (**D**) Quantification of the number of neuromuscular junctions re-innervated at 2 months after sciatic nerve injury. There was a significant reduction in the number of re-innervated neuromuscular junctions in injured conNrg1 animals compared with injured vehicle control animals (*P < 0.05 one-way ANOVA *post hoc* Tukey), *n* = 3. (**E**) Sciatic Functional Index (SFI) was used to assess the functional recovery following sciatic nerve crush. Animals were tested at baseline and post-injury. In conNrg1 mutant animals, functional recovery was significantly slower than vehicle and tamoxifen control animals. conNrg1 versus vehicle control: **P < 0.005 ***P < 0.001 conNrg1 versus tamoxifen control ^#^*P* < 0.05 ^##^*P* < 0.005 two-way repeated measures ANOVA *post hoc* Tukey. Tamoxifen control, *n* = 7–8. (**F**) Representative traces of compound nerve action potentials recorded from the distal tibial nerve after proximal (at crush site) sciatic nerve stimulation of vehicle and conNrg1 mutants. Recording from the uninjured nerve, there is no change in the latency or the amplitude, whereas in the injured nerve, there is an increase in latency and a decrease in the amplitude in mutant animals. (**G**) Quantification of the conduction velocity. (**H**) Quantification of the amplitude. *P < 0.05 two tailed Students *t*-test, *n* = 4. Tx = tamoxifen; Vh = vehicle.

To test whether disrupted axoglial signalling caused by NRG1 ablation leads to a change in the target reinnervation by sensory and motor neurons, the innervation of the epidermis and the gastrocnemius muscle were analysed 8 weeks following sciatic nerve injury. Re-innervation of the epidermis (Fig. 5A and B) and of the gastrocnemius muscle (Fig. 5C and D) was less effective in conNrg1 mutants. In addition, no axons innervating the muscle had a myelin sheath as assessed by MBP staining (Fig. 5C). Thus, distal motor axons innervating the gastrocnemius muscle had failed to remyelinate in conNrg1 animals.

### NRG1 is required for the early phase of functional recovery following peripheral nerve injury

Functional recovery after peripheral nerve injury was assessed using the sciatic functional index. Animals developed a severe functional deficit immediately following sciatic nerve crush, which was consistently observed in control as well as conNrg1 mutant mice 10 days post injury (Fig. 5E). At 14 days post-injury, control animals showed significantly improved functional output compared to conNrg1 mutant. Differences in the functional recovery between control and conNrg1 mutant mice persisted until 56 days post-injury, but became less pronounced at late stages. Functional recovery was further examined by performing electrophysiology on control and conNrg1 mutant mice 2 months after injury on the injured sciatic nerve and on the contralateral uninjured nerve (Fig. 5F–H). In the uninjured nerve, neither the conduction velocity nor the amplitude of the compound nerve action potential was affected by *Nrg1* ablation (Fig. 5F–H). Following sciatic nerve injury, there was a decrease in nerve conduction in both controls and conNrg1 mutants; however, this reduction was more pronounced in conNrg1 mutants (Fig. 5G). Similarly, the compound nerve action potential amplitude was diminished following peripheral nerve injury in both groups; however, it was more severely affected after *Nrg1* ablation (Fig. 5H). *Nrg1* ablation during adulthood therefore results in reduced functional recovery following peripheral nerve injury.

### NRG1 regulates the rate of remyelination but myelination fate is not absolutely dependent on axonal NRG1

**Figure 6:**
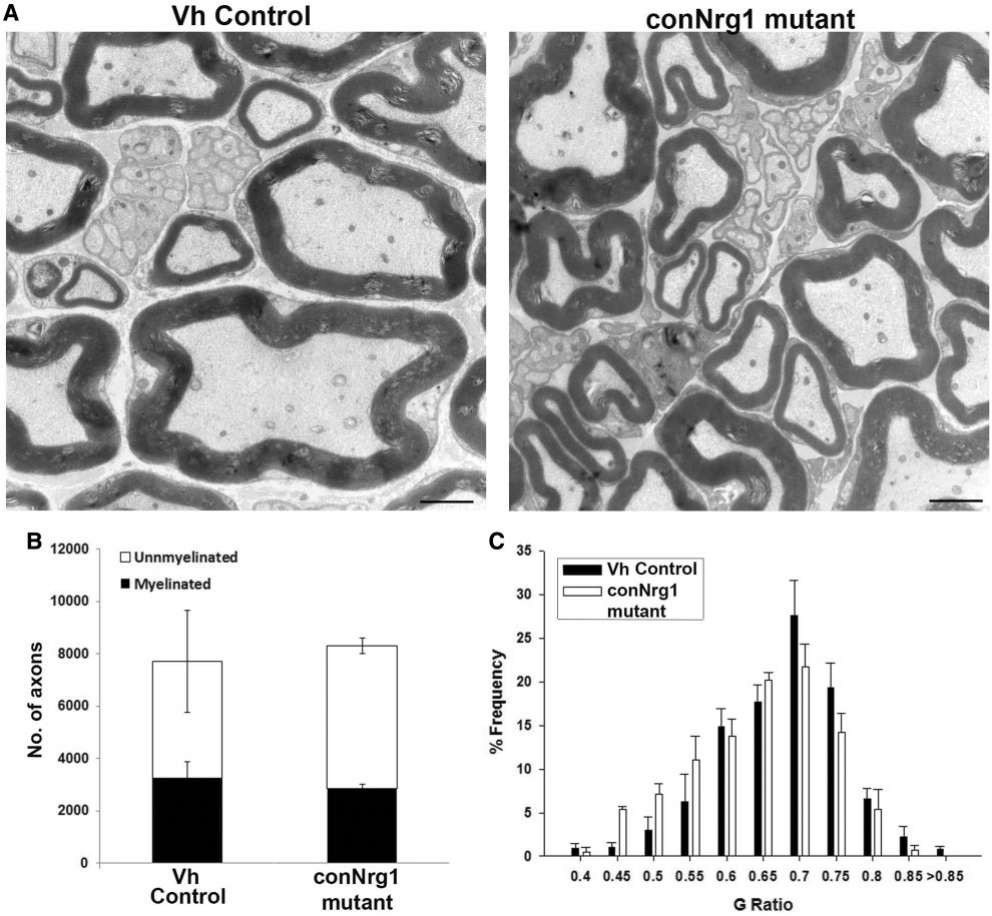
Axonal NRG1 is not essential for remyelination at longer time points after injury. (**A**) Electron micrographs of sciatic nerve distal to sciatic nerve transection and re-anastomosis lesion site 3 months after injury. Note effective remyelination in conNrg1 animals. Scale bars = 2 µm. (**B**) Counts of myelinated and unmyelinated axons in vehicle control and conNrg1 animals, neither the total number of axons nor the proportion that are myelinated is altered in the absence of Nrg1. (**C**) G-ratio frequency distribution demonstrating no increase in G-ratio in the remyelinated sciatic nerve fibres from the conNrg1 animals relative to control. *n* = 3.

We also studied the role of NRG1 in a more severe model of nerve injury: sciatic nerve transection and reanastamosis (Supplementary Fig. 1B). Because of the severity of the injury, we analysed remyelination 8 and 12 weeks post-injury, i.e. at later stages than that analysed in the previous models. Eight weeks post-transection and reanastamosis, remyelination was significantly impaired in the conNrg1 mutant mice, similar to what we had seen in the nerve crush model (Supplementary Fig. 4A). However, 12 weeks after transection, efficient remyelination was observed in conNrg1 mutants (Fig. 6A–C), in particular an equivalent proportion of axons were remyelinated (Fig. 6B), and myelin thickness was restored to normal levels (G-ratios mean ± SEM; vehicle control 0.653 ± 0.008 conNrg1 0.619 ± 0.013) (Fig. 6C). Remak bundle morphology was normal in conNrg1 mutants. Distally in the gastrocnemius muscle, remyelination was also evident in conNrg1 animals (Supplementary Fig. 4C), although there were segments of axons that were still naked. Functional recovery was poor in all animals independent of their genotype (Supplementary Fig. 4B). Poor functional recovery in mice following this injury paradigm has previously been reported and is a result of inappropriate/ineffective reinnervation (Monk *et al.*, 2011). Despite the remyelination observed, neuronal NRG1 protein levels were still low and had not recovered (Supplementary Fig. 4D).

Schwann cell derived NRG1 has been shown to drive remyelination, and an early upregulation of NRG1 type I is seen in nerves of wild-type mice following sciatic nerve injury (Stassart *et al.*, 2013). Consequently, a residual source of NRG1 potentially from Schwann cells that have escaped genomic recombination, could account for the delayed remyelination phenotype observed. To examine this effect, we determined levels of αNRG1 isoforms compared to βNRG1 isoforms in nerves of wild-type mice when uninjured or 1 or 10 days post-sciatic nerve injury (Supplementary Fig. 5). βNRG1 isoforms only showed a slight non-significant increase following injury whereas there was a more than 10-fold significant increase in αNRG1 isoforms at 1 day post-injury, which was sustained at 10 days post-injury. Considering all of our experimental animals (excluding tamoxifen control animals) are αNRG1 null mutants there is no functional αNRG1 following nerve injury. In addition, 3 months post-injury levels of βNRG1 are still greatly reduced when measured at the messenger RNA (10.3 ± 3.3% of vehicle control) and at the protein level (Supplementary Fig. 6). Thus, NRG1 regulates the rate of remyelination at early phases of repair, but at later stages remyelination can occur effectively in a manner that appears largely independent of NRG1 and clearly myelination fate is no longer determined by the level of NRG1 expressed on the axolemma.

### The developmental consequences of NRG1 ablation are not compensated for at long survival times in adulthood

**Figure 7:**
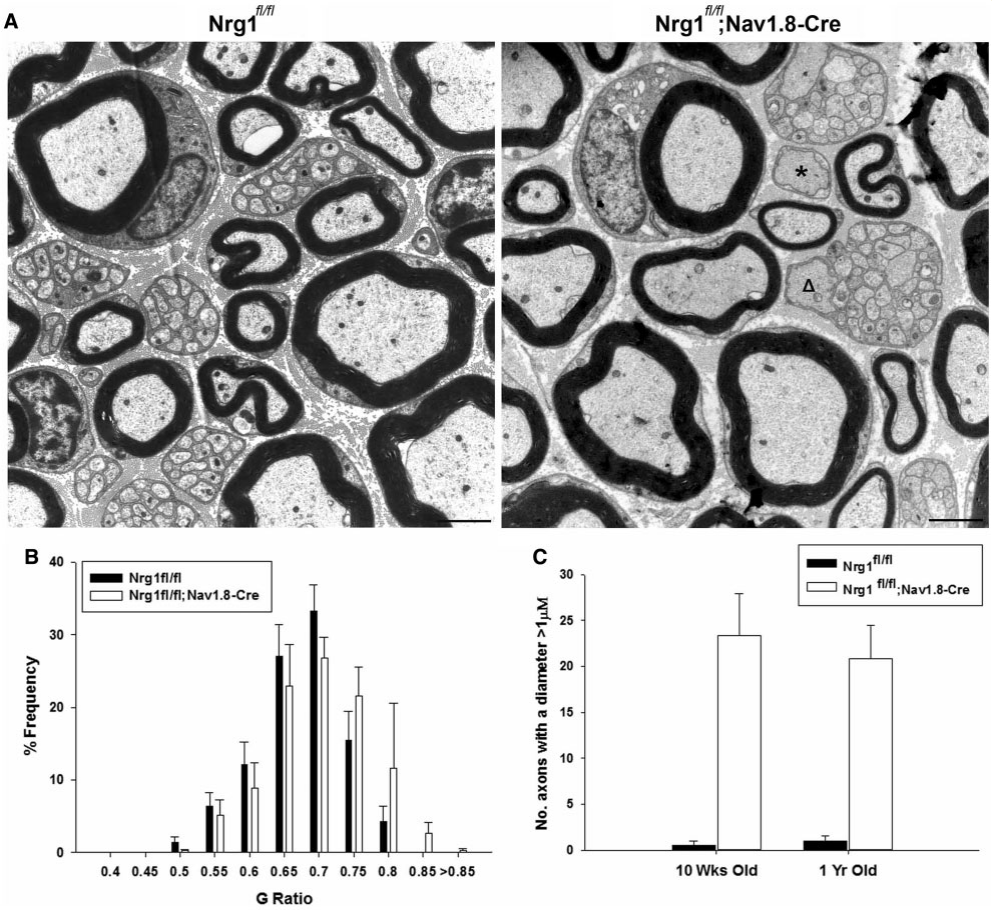
Axonal Nrg1 is required for Remak bundle structure and myelination in development and this is not compensated for over long periods of time. (**A**) Electron micrographs of sural nerves from 1-year-old Nrg1*^fl/fl^* control mice and Nrg1*^fl/fl^*;Nav1.8-Cre mice in which Nrg1 was ablated in a subpopulation of sensory neurons. The developmental phenotype characterized at 10 weeks of age (Fricker *et al.*, 2009), of large unordered Remak bundles, within which axons are not separated by Schwann cell processes and which also contain axons with a diameter >1 µm (open triangle) persists at this age. Also singly sorted axons >1 µm in diameter and surrounded by a Schwann cell remain unmyelinated (asterisk). Scale bar = 2 µm. (**B**) G-ratio frequency distribution shows there is still a shift to larger G-ratios in the Nrg1*^fl/fl^*;Nav1.8-Cre mice, indicating a proportion of axons with thinner myelin sheaths (*P* < 0.001 Kolmogorov-Smirnov test) *n* = 3. (**C**) Counts of the number of unmyelinated axons in a 1:1 relationship with a Schwann cell and >1 µm in diameter as depicted in **A** by an asterisk, at both 10 weeks and 1 year of age. In summary there is no recovery in the developmental phenotype at 1 year of age, *n* = 3–4.

To see whether over time there is compensation by signals apart from axonal NRG1 in developmental myelination, we examined 1-year-old nerves of Nrg1*^fl/fl^*;Nav1.8-Cre mice. In such animals, recombination of the *Nrg1* allele occurs early and when examining the sural nerve of 10-week-old animals, we had previously described severely disrupted Remak bundle structure, thinner myelin or no myelin sheath (Fricker *et al.*, 2009) in a subset of axons. The phenotype observed in 10-week-old mice was still clearly present at an age of 1 year (Fig. 7), indicating that compensation of the developmental phenotype does not occur. Thus, axonal NRG1 controls developmental myelination and remyelination after injury in a distinct manner.

### NRG1 regulates multiple myelin-related gene transcripts following nerve injury

**Figure 8:**
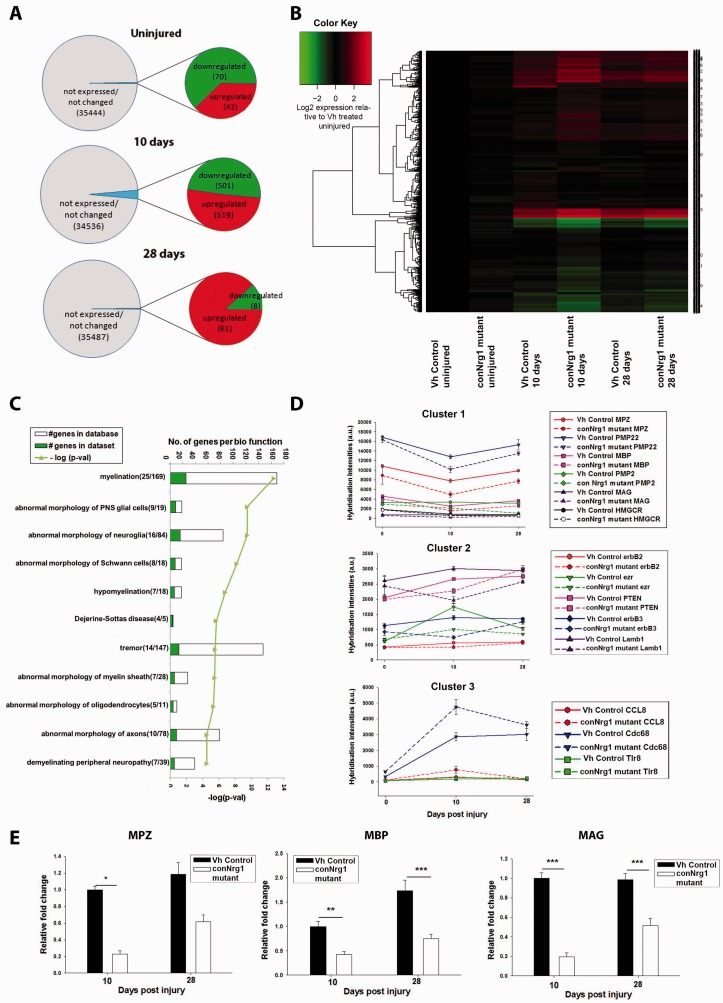
Myelin gene-related transcriptional changes in conditional Nrg1 mutants after peripheral nerve injury. (**A**) Comparison of sciatic nerve transcriptional profiles in conNrg1 and vehicle control animals in the naive state and at distinct time points (10 days and 28 days) after sciatic nerve crush. Out of a total of 35 556 transcripts probed in the mouse gene 1.0 ST microarray chip, only a very modest number were differentially expressed (*P* < 0.1, FDR) in uninjured states (*top*). In contrast, NRG1 ablation has a larger impact on gene expression 10 days after injury with ∼3% of the probes being differentially expressed (*middle*). At 28 days post-injury, the number of differentially expressed transcripts was similar to baseline levels. (**B**) Heat map representing levels of gene expression in all experimental groups for the subset of genes showing differential expression at the 10 day post-injury time point. Gene expression levels were normalized to uninjured nerve from vehicle control animals and values of expression were log2 transformed. (**C**) Ingenuity pathway analysis of all genes downregulated at 10 days post-injury in the conNRG1 animals revealed an enrichment for myelination-related biological function. Numbers in brackets represent number of genes with assigned biofunction in the data set/total number of genes assigned to this pathways based on literature curation. (**D**) Cluster analysis of coregulated genes (Pearson correlation ≥0.7, Biolayout Express 3D), revealed three main clusters. Graphical representation of hybridization intensities (average ± SEM) in each experimental group for illustrative example genes in each cluster. (**E**) Confirmation using real-time quantitative PCR of some of the transcriptional changes detected by microarrays. Levels of *Mpz*, *Mbp* and *Mag*, 10 and 28 days after injury. Messenger RNA expression levels were normalized to the reference gene *GAPDH* and depicted as relative fold change compared to uninjured nerve from vehicle control animals, (one-way ANOVA, *post hoc* Tukey,*P < 0.05 ***P < 0.001).

We performed genome-wide transcription profiling using Affymetrix Gene Chip Mouse Gene 1.0 ST Arrays (Fig. 8) in order to identify genes deregulated in the absence of NRG1 signalling or genes downstream of NRG1 signalling during nerve repair. We performed differential expression analysis between treatments (conNrg1 versus vehicle control groups) in naive and 10 and 28 days post-crush. We considered fold changes with an FDR adjusted *P*-value of *P* < 0.1 as significant. This analysis demonstrated a minimal effect of *Nrg1* ablation, and only 112 transcripts (out of 35 556 transcript clusters probed in the chip) were differentially expressed in uninjured nerves in adult control and conNrg1 mutant animals (Fig. 8 A). This number was greatly increased at 10 days post-injury when ∼1000 genes were differentially expressed (Fig. 8A and B). At 28 days post-injury, the number of deregulated genes was again small, and we identified 69 differentially expressed transcripts when nerves of control and conNrg1 mutants were compared (Fig. 8A). Because the majority of transcriptional changes occurred 10 days post-injury, the remaining functional and clustering analyses were performed on this list of deregulated genes (Fig. 8B). Ingenuity pathway analysis revealed a highly significant overrepresentation of biological functions related to myelination among the deregulated genes (Fig. 8C; the full data set is shown in Supplementary Table 1). In particular, the myelin-related genes displayed downregulated expression at 10 days post-injury in control versus conNrg1 mutant animals.

Graphical analysis of levels of gene expression normalized to uninjured control nerve suggested a number of distinct patterns of expression across time points and groups (Fig. 8B). We identified clusters of co-regulated transcripts (Pearson correlation ≥ 0.7) using Biolayout Express3D (Theocharidis *et al.*, 2009) (Fig. 8D). The majority of genes could be clustered into three main patterns according to the time courses of expression in the nerves of control and conNrg1 mutant mice (Fig. 8D). Cluster 1 contains genes that were downregulated in injured nerves at 10 days post-injury in both control and conNrg1 mutants, and which tended to return towards baseline levels at 28 days. Their overall temporal pattern of expression observed in the nerves of the conNrg1 mutant animals was similar, but the downregulation at the 10-day time point was more pronounced. This cluster included a number of genes with well documented functions in myelination, like the myelin genes *Mbp*, *Mpz* and *Mag*, lipid synthesis enzymes such as *Hmgcr*, and components of other pro-myelination signalling pathways such as *Fzd3*, a Wnt receptor (Tawk *et al.*, 2011), and *GPR126* (Monk *et al.*, 2011) (Fig. 8D). Ingenuity pathway analysis revealed an enrichment for ‘myelination’ function. Real-time quantitative PCR confirmed the downregulation of expression of *Mag*, *Mbp* and *Mpz* genes in mutant nerves (Fig. 8E).

Genes in Cluster 2 displayed different time courses in gene expression. For example, *Erbb3* messenger RNA was upregulated in control mice after injury, but in *Nrg1* mutant nerves, the levels of expression were significantly reduced in the injured compared to the uninjured nerve. Conversely transcripts encoding *Ezr* were upregulated in the injured nerve of control mice but remained unchanged in the nerves of mutant mice. Overall this cluster is also enriched for myelination-related genes, albeit less prominently than Cluster 1.

Cluster 3 corresponds to genes upregulated following nerve injury in both treatment groups. Although these genes display similar time courses (upregulated at 10 days and back to baseline levels by 28 days), their upregulation was more pronounced in the nerves of *Nrg1* mutants. Ingenuity pathway analysis revealed an enrichment for genes functioning in immune response, suggesting a more pronounced activation of the immune system in the absence of functional NRG1. Interestingly Schwann cells were recently shown to participate in the initial immune response to nerve injury (Napoli *et al.*, 2012). We conclude that in the naive state, NRG1 is not required for maintenance of myelin gene expression program. However, after nerve injury NRG1 signalling promotes a broad transcriptional programme of myelin-related genes especially at early stages of nerve repair (10 days).

## Discussion

We report here the function of NRG1 in nerve repair and functional recovery in the PNS. All NRG1 isoforms were ablated in adulthood using a conditional genetic strategy. In the naive state, NRG1 was dispensable for the maintenance of the myelin sheath. Following injury, Schwann cell proliferation associated with axon degeneration or regeneration occurred independently of axonal NRG1 but at early time points after nerve injury, impaired functional recovery was observed after *Nrg1* ablation. We found that NRG1 drives a general pro-myelination transcriptional response in Schwann cells during nerve repair, and in accordance remyelination was impaired during early stages of nerve repair when *Nrg1* was ablated. In contrast to developmental myelination, however, the determination of myelination fate and myelination thickness in the late phase of nerve repair did not depend on axonal NRG1.

In agreement with previous studies using conditional ablation of the ERBB2 receptor in Schwann cells (Atanasoski *et al.*, 2006) or NRG1 in subsets of peripheral neurons (Fricker *et al.*, 2011) conditional ablation of NRG1 within the PNS in the naive state did not result in any change in axon or myelin morphology or target innervation compared with controls. This is in contrast with the key transcriptional regulators of myelination, *Krox20* (now known as *Egr2*) and *Sox10* whose continued expression is necessary for myelin maintenance in adulthood (Le *et al.*, 2005; Decker *et al.*, 2006; Bremer *et al.*, 2011).

Nerve injury and repair is associated with two phases of Schwann cell proliferation: the first phase is due to loss of axonal contact and peaks around 3 days post-injury (Bradley and Asbury, 1970; Stoll *et al.*, 1989; Carroll *et al.*, 1997; Stoll and Muller, 1999; Griffin and Thompson, 2008). The second phase of proliferation occurs as axons enter the denervated nerve stump (Pellegrino and Spencer, 1985). Because of the role of NRG1 in Schwann cell proliferation during development (Dong *et al.*, 1995; Parkinson *et al.*, 2004; Perlin *et al.*, 2011) and in the post-natal period (Chen *et al.*, 2003), we investigated the role of NRG1 in modulating Schwann cell proliferation following injury. The proliferative response to axon degeneration was not altered in the absence of NRG1. Similarly, in mice in which ERBB2 was inducibly ablated in adult Schwann cells, no change in Schwann cell proliferation was seen at 4 or 12 days post-injury (Atanasoski *et al.*, 2006). Axons are known to be mitogenic for Schwann cells in culture preparations (Salzer *et al.*, 1980), and there is evidence that this is in part mediated by NRG1 signalling (Morrissey *et al.*, 1995). Although we found a clear Schwann cell proliferative response to regenerating axons, this also was not dependent on NRG1, indicating that other axon-derived signals stimulating proliferation exist. Such a signal might be provided by NOTCH1, which regulates Schwann cell proliferation during development (Woodhoo *et al.*, 2009).

NRG1 signalling has been implicated as an important pathway in the repair process following peripheral nerve injury (Fricker and Bennett, 2011). An early event in peripheral nerve repair involves the recruitment of macrophages and the clearance of myelin debris; we found no impairment in this process in animals in conNrg1 mutant animals. Consistent with our previous report of impaired remyelination in individual axons where juxtacrine NRG1 signalling is ablated (Fricker *et al.*, 2011), or in animals lacking BACE1, an enzyme required for the proteolytic cleavage of NRG1 into its active form (Hu *et al.*, 2008), we saw an essential role of NRG1 in promoting remyelination in early phases of peripheral nerve repair. Within the sciatic nerve distal to the crush site, remyelination had commenced at 10 days post-injury in the control nerve with many axons ensheathed by compact myelin. Following conditional *Nrg1* ablation almost no myelinated axons were observed at this time point. At later time points up to 2 months post-injury, progressively more myelinated axons were observed in the conNrg1 mutant animals albeit with a significant increase in the G-ratio.

The importance of NRG1 in remyelination is emphasized by a broad pro-myelination transcriptional programme driven by this factor. In the naive state there were few alterations in gene expression in the absence of NRG1. Following nerve injury, myelin-related genes were significantly over-represented in those genes which were downregulated after ablation of *Nrg1*. However, the down-regulation in myelin-related genes in the absence of NRG1 was most apparent at early rather than late time points post-injury.

Consistent with our previous findings that axonal NRG1 can modulate the rate of long range axon regeneration there was a small but significant deficit in reinnervation of the neuromuscular junction following sciatic nerve crush in the absence of NRG1. The use of recombinant or virally expressed NRG1 therapeutically following peripheral nerve injury has shown some efficacy in promoting axon regeneration (Chen *et al.*, 1998; Joung *et al.*, 2010; Fricker and Bennett, 2011; Yildiz *et al.*, 2011). This may be due to the release of neurotrophic factors from Schwann cells in response to NRG1 (Mahanthappa *et al.*, 1996), or potentially even direct effects on axons (Terenghi, 1999).

Using the sciatic functional index as a measure of functional recovery we noted significant slowing in the rate of recovery following sciatic nerve crush in the absence of NRG1. Interestingly in conNrg1 mice recovery was delayed rather than prevented and by 42 days post injury there was no longer a significant difference. In addition to sciatic nerve crush, we therefore also examined a more severe injury model over a longer time course: sciatic nerve transection and reanastamosis.

At 2 months post-sciatic transection and reanastamosis, we noted as expected deficient remyelination in mice that lacked NRG1. Interestingly, at the late time point i.e. 3 months after this injury, effective remyelination had been achieved even in the absence of NRG1. During development, axonal expression of NRG1 type III is absolutely required for axon ensheathment and subsequent myelination (Michailov *et al.*, 2004; Taveggia *et al.*, 2005). The thickness of the myelin sheath is modulated by the level of NRG1 type III expression on the axon. Haplo-insufficiency of NRG1 results in a thinner myelin sheath (Michailov *et al.*, 2004; Taveggia *et al.*, 2005). Neuronal over-expression of type III but not type I NRG1 results in a thicker myelin sheath. Although the key promyelinating cue is thought to be juxtacrine NRG1 type III, soluble type I or type III NRG1 can promote myelination *in vitro* at low doses (high doses of soluble NRG1 promote demyelination) (Zanazzi *et al.*, 2001; Syed *et al.*, 2010). Recently soluble Schwann cell-derived type I NRG1 has been shown to promote remyelination following peripheral nerve injury (Stassart *et al.*, 2013).

Although NRG1 determines the rate of remyelination at early time points following injury, we show here that at later time points myelin thickness is no longer regulated by the level of expression of NRG1 on the axolemma. During development, axon diameter is known to have a key role in determining myelination fate (Duncan, 1934; Voyvodic, 1989). In the PNS, axons >1 µm in diameter are myelinated, and the higher levels of NRG1 type III expression in large diameter axons provides a logical mechanism for such a relationship (Taveggia *et al.*, 2005). At 3 months post-sciatic nerve transection and repair, axon diameter was still a key factor in determining whether axons were remyelinated, however remyelination was not dependent on axonal expression of NRG1.

We have conditionally ablated *Nrg1* using a globally expressed Cre-transgene and have not examined the distinct role of Schwann cell versus axon derived NRG1 in nerve repair and remyelination. Stassart *et al.* (2013) report an immediate increase in NRG1 type I expression in Schwann cells following nerve injury, which is sustained up to Day 14. This Schwann cell-derived NRG1 promotes remyelination at early time points following nerve injury. Given our previous findings (Fricker *et al.*, 2011), Schwann cell-derived NRG1 type 1 is not able to fully substitute for a lack of axon-derived juxtacrine NRG1 type III. The role of Schwann cell derived NRG1 on the late phase of nerve repair has not been examined. We find that it is primarily the α and not β EGF domain containing NRG1 isoforms that show increased expression following nerve injury; vehicle control and conNrg1 mutant animals are global αNRG1 null mutants and Cre-mediated recombination results in efficient ablation of βNRG1 isoforms within nerve. Therefore we consider it unlikely that Schwann cell-derived NRG1 is contributing to the late remyelination phenotype we observed although we cannot completely exclude the possibility that a small amount of βNRG1 expression persists within the nerve or is available from other sources such as the circulation.

Coordinated signals provided by the axon and the extracellular matrix are likely to be integrated by Schwann cells for effective remyelination. It is possible that alternative signalling systems may compensate for the absence of axonal NRG1 at later stages of repair (Taveggia *et al.*, 2010). It is becoming increasingly clear that nerve repair and remyelination are not simply a recapitulation of development (Fancy *et al.*, 2011). Following axonal injury, distinct signalling pathways and transcriptional programmes drive the dedifferentiation of Schwann cells (Arthur-Farraj *et al.*, 2012; Napoli *et al.*, 2012). The population of Schwann cells that appear after nerve injury is distinct from that of developing immature Schwann cells, and these cells are specialized to support nerve repair (Arthur-Farraj *et al.*, 2011). Our findings suggest that NRG1 has a distinct role in axoglial signalling during nerve repair in adulthood versus its development role in both, non-myelinating and myelinating Schwann cells. During development non-myelinating Schwann cells require axonal NRG1 to establish and maintain normal Remak bundle structure (Taveggia *et al.*, 2005; Fricker *et al.*, 2009). In contrast, non-myelinating Schwann cells re-establish normal Remak bundles after peripheral nerve injury in animals in which NRG1 is conditionally ablated. This is likely a reflection of the fact that axon sorting is not required in adulthood, and basal laminae have been established. Similarly, in myelinating Schwann cells, NRG1 is required for developmental myelination and acts to accelerate the early phase of remyelination during repair; however, at later stages of repair the myelination fate is no longer absolutely dependent on axonal expression of this factor.

## Supplementary Material

Supplementary Data

## Abbreviations

BrdU: 5-Bromo-2’-deoxyuridine
conNrg1: conditional neuregulin 1
