# Supplementary material for: Axonal neuregulin 1 is a rate limiting but not essential factor for nerve remyelination
Source: Brain. 2013 Jun 24;136(7):2279–97. doi: 10.1093/brain/awt148 (PMC3692042; doi:10.1093/brain/awt148)
Supplement: Supplementary Data [file supp_136_7_2279__index.html]

Axonal neuregulin 1 is a rate limiting but not essential factor for nerve remyelination — Supplementary Data 

# Axonal neuregulin 1 is a rate limiting but not essential factor for nerve remyelination

## 

files

**Files in this Data Supplement:**

- Supplementary Data - docx file
- Supplementary Data - tif file
- Supplementary Data - tif file
- Supplementary Data - tif file
- Supplementary Data - jpg file
- Supplementary Data - tif file
- Supplementary Data - docx file
- Supplementary Data - doc file
