# Supplementary material for: Axonal neuregulin 1 is a rate limiting but not essential factor for nerve remyelination
Source: Brain. 2013 Jun 24;136(7):2279–97. doi: 10.1093/brain/awt148 (PMC3692042; doi:10.1093/brain/awt148)
Supplement: Supplementary Data [file supp_awt148_brain-2012-02167-File010.docx]

**Supplementary Methods**

Primers (all 5’-3’):

| Primers to: | Forward | Reverse | Notes/References |
| --- | --- | --- | --- |
| **CAG-Cre-ER^TM^ construct** | CTCTAGAGCCTCTGCTAACC | CCTGGCGATCCCTGAACATGTCC |  |
| **βEGF-Nrg1** | CGTAATGGCCAGCTTCTACA | CCATGTTGTTTCGTTCTGACC | Designed specifically detect the excision of the 59bp sequence encoding the β-EGF domain that is floxed in these animals and excised by Tx treatment |
|  | CATCTACATCCACGACTGG ([Makinodan *et al.*, 2012](#_ENREF_2)) | CGTAGTTTTGGCAACGATCACC  (Cheret *et al., unpublished*) |  |
| **αEGF-Nrg1** | CATCTACATCCACGACTGG ([Makinodan *et al.*, 2012](#_ENREF_2)) | CTTTCATGGGCACATTCTCA  (Cheret *et al., unpublished*) |  |
| **GAPDH** | TGTGTCCGTCGTGGATCTGA | TTGCTGTTGAAGTCGCAGGAG |  |
| **MPZ** | GTCAAGTCCCCCAGTAGAA | AGGAGCAAGAGGAAAGCAC | ([Tawk *et al.*, 2011](#_ENREF_3)) |
| **MBP** | CTCCCTGCCCCAGAAGTCGC | CTCTTCCTCCCCAGCTAAATCTTGC |  |
| **MAG** | TGGGCCTACGAAACTGTACC | GCTCCGAGAAGGTGTACTGG | ([Lin *et al.*, 2006](#_ENREF_1)) |

Lin T, Xiang Z, Cui L, Stallcup W, Reeves SA. New mouse oligodendrocyte precursor (mOP) cells for studies on oligodendrocyte maturation and function. Journal of neuroscience methods. 2006;157(2):187-94.

Makinodan M, Rosen KM, Ito S, Corfas G. A critical period for social experience-dependent oligodendrocyte maturation and myelination. Science. 2012;337(6100):1357-60.

Tawk M, Makoukji J, Belle M, Fonte C, Trousson A, Hawkins T, et al. Wnt/beta-catenin signaling is an essential and direct driver of myelin gene expression and myelinogenesis. The Journal of neuroscience : the official journal of the Society for Neuroscience. 2011;31(10):3729-42.
