# Supplementary figures and images for: Axonal neuregulin 1 is a rate limiting but not essential factor for nerve remyelination
Source: Brain. 2013 Jun 24;136(7):2279–97. doi: 10.1093/brain/awt148 (PMC3692042; doi:10.1093/brain/awt148)

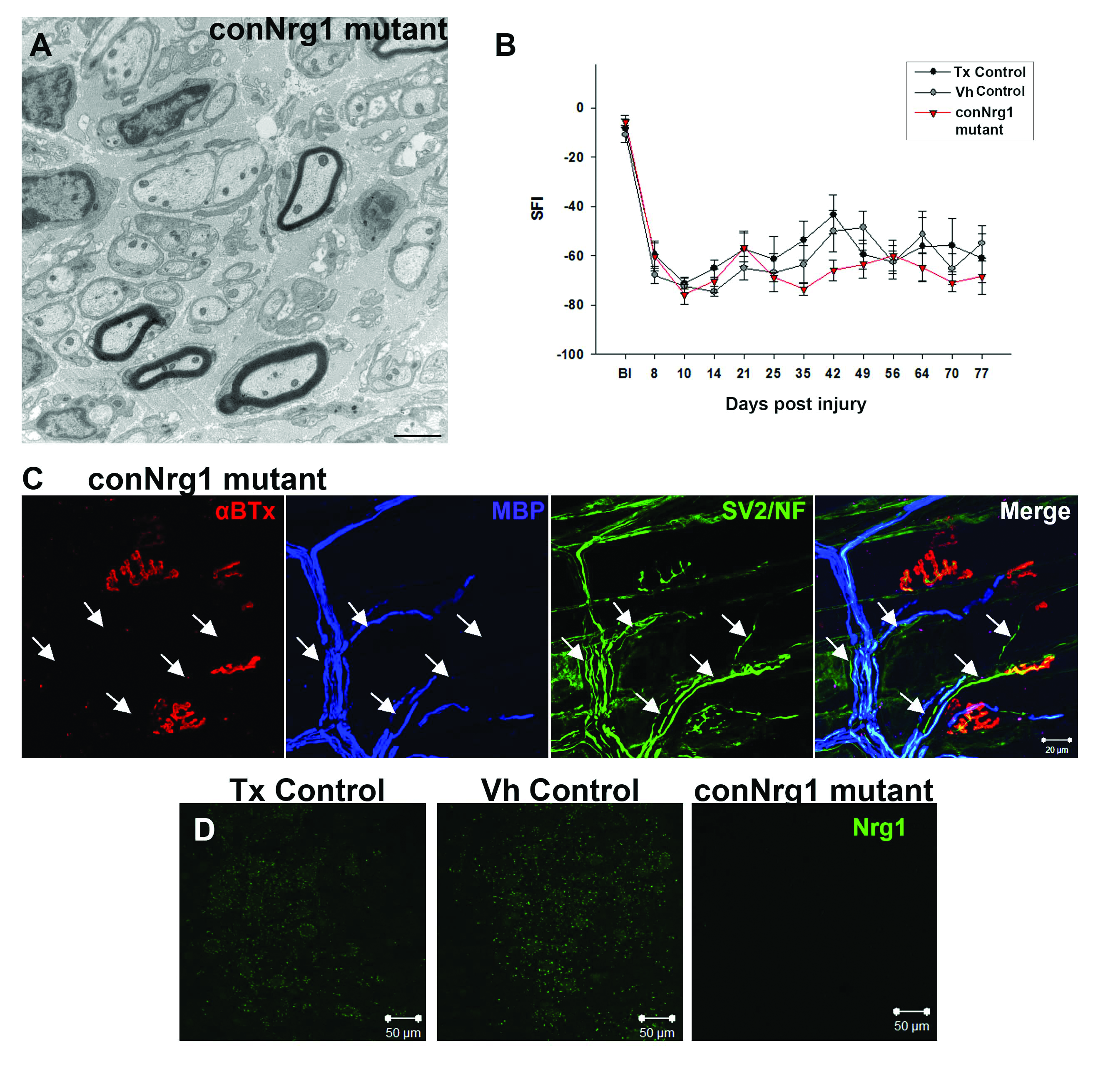

Supplement: Supplementary Data [file supp_awt148_brain-2012-02167-File012.tif]

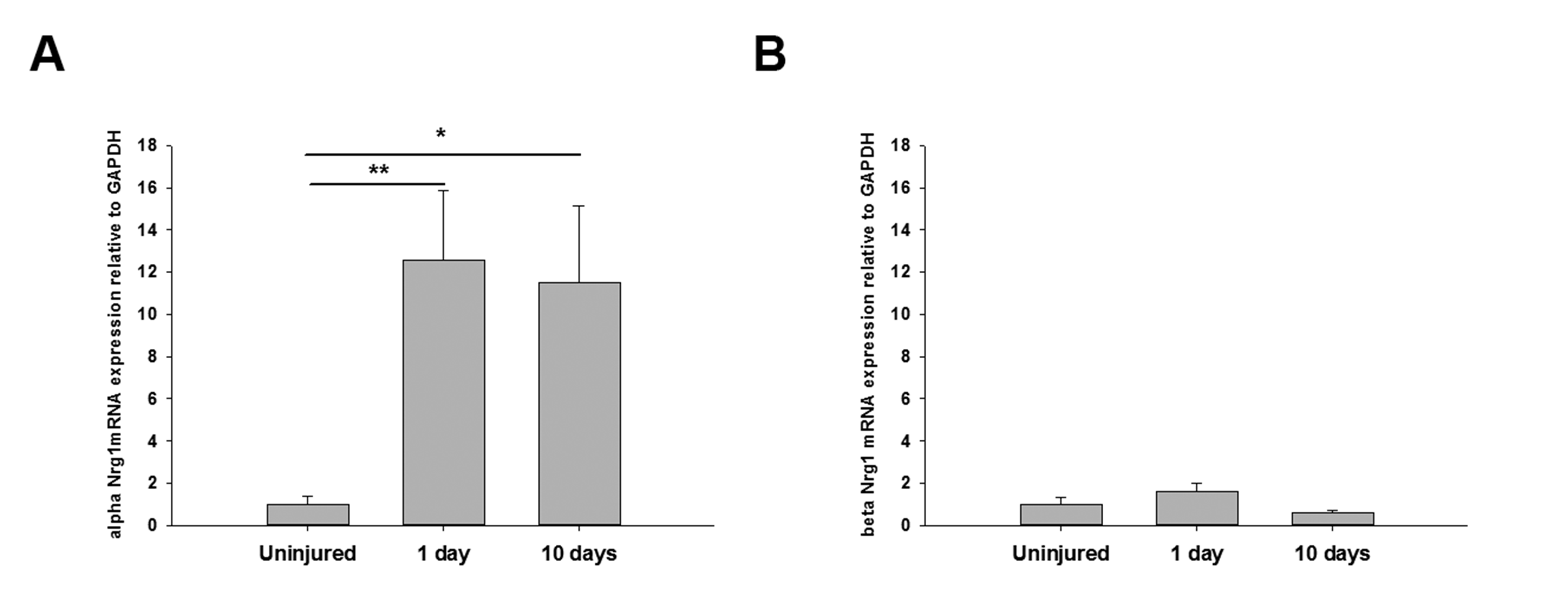

Supplement: Supplementary Data [file supp_awt148_brain-2012-02167-File013.tif]

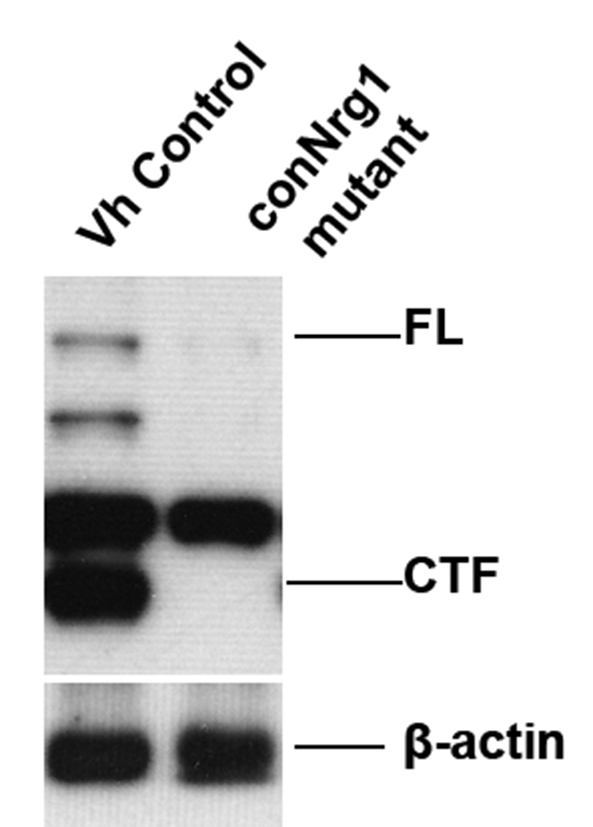

Supplement: Supplementary Data [file supp_awt148_brain-2012-02167-File014.tif]

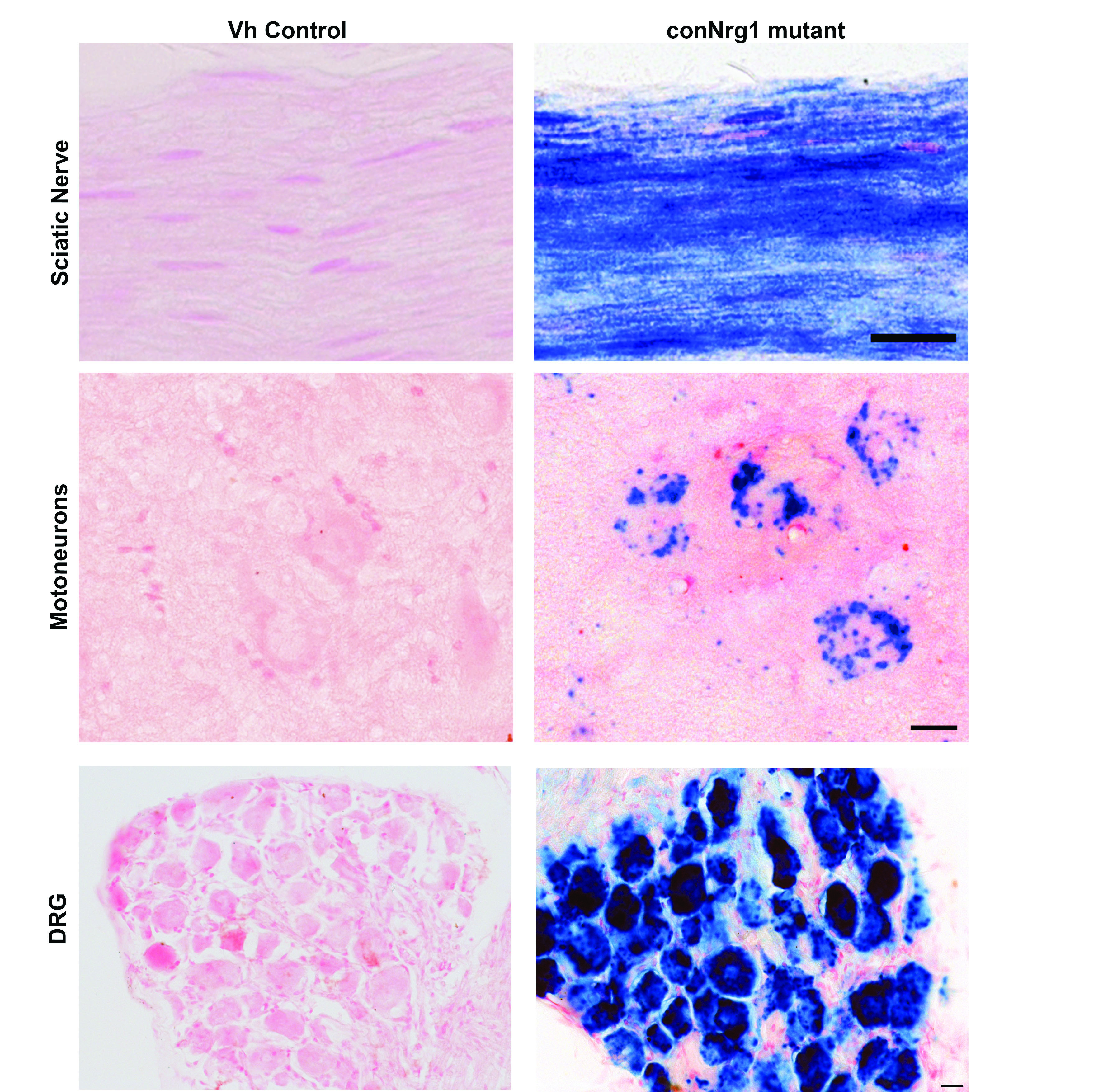

Supplement: Supplementary Data [file supp_awt148_brain-2012-02167-File015.jpg]

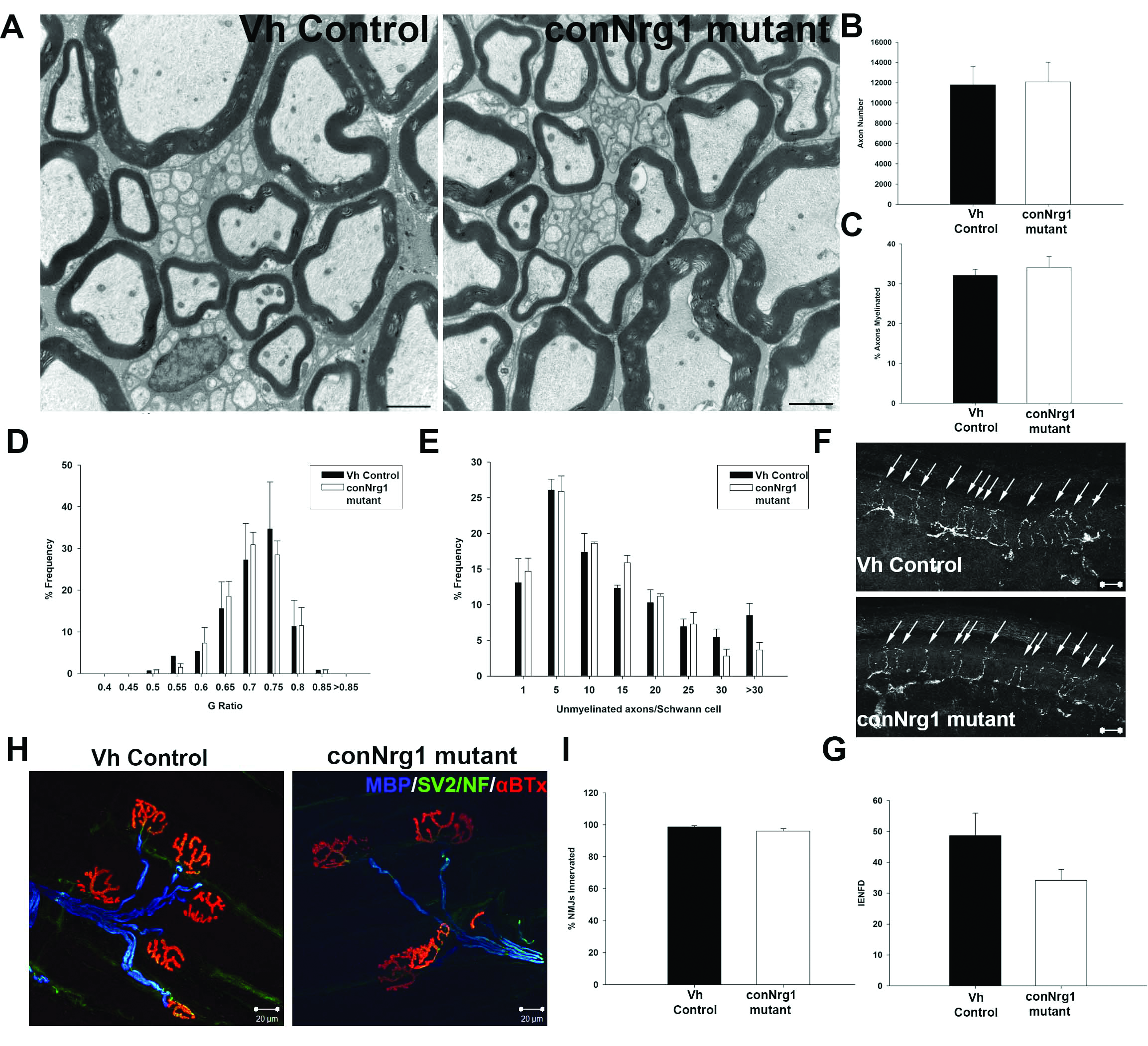

Supplement: Supplementary Data [file supp_awt148_brain-2012-02167-File016.tif]
