# Supplementary material for: Axonal neuregulin 1 is a rate limiting but not essential factor for nerve remyelination
Source: Brain. 2013 Jun 24;136(7):2279–97. doi: 10.1093/brain/awt148 (PMC3692042; doi:10.1093/brain/awt148)
Supplement: Supplementary Data [file supp_awt148_brain-2012-02167-File017.docx]

Table 1- Transcripts differentially expressed between tx and vh-treated animals in the injured sciatic nerve 10d post injury. Levels of expression correspond to hybridization intensities after normalization, background subtraction and summarization of individual probe sets to the “transcript cluster level”, and are indicated as averages for each of the experimental groups considered. Log 2 Fold change is also indicated as well as respective adjusted p-value (only transcripts with FDR p-value <0.1 are indicated).

| Cluster | **Affymetrix transcript cluster ID** | Gene Symbol | Average levels of expression (arbitrary units) | | | | | | **Fold change**  **Tx_10d_ injured /Vh_10d_ injured** | |
| --- | --- | --- | --- | --- | --- | --- | --- | --- | --- | --- |
|  |  |  | **Vh_10d_ uninjured** | **Vh_10d_ injured** | **Vh_28d_ injured** | **Tx_10d_ uninjured** | **Tx_10d_ injured** | **Tx_28d_ injured** | **Log (2) Fold change** | **Adj. p-value** |
| 1 | 10338107 |  | 754.46 | 855.88 | 780.30 | 832.33 | 583.69 | 762.59 | -0.5732 | 0.0097 |
| 1 | 10338181 |  | 29.58 | 28.84 | 31.05 | 23.38 | 17.37 | 26.57 | -0.7207 | 0.0749 |
| 1 | 10338601 |  | 4320.47 | 1908.05 | 2399.28 | 4219.20 | 1261.61 | 2078.06 | -0.6066 | 0.0207 |
| 1 | 10339203 |  | 243.82 | 242.50 | 200.54 | 214.74 | 143.37 | 195.13 | -0.8029 | 0.0399 |
| 1 | 10339653 |  | 6457.61 | 3680.38 | 4477.03 | 6601.28 | 2722.00 | 4169.83 | -0.4372 | 0.0216 |
| 1 | 10340219 |  | 7382.15 | 3662.87 | 4560.94 | 6865.90 | 2646.40 | 4015.05 | -0.4806 | 0.0116 |
| 1 | 10340406 |  | 712.07 | 400.58 | 473.97 | 764.71 | 241.25 | 401.65 | -0.8263 | 0.0840 |
| 1 | 10340423 |  | 2112.10 | 902.08 | 1215.64 | 2153.72 | 601.38 | 1028.49 | -0.6040 | 0.0326 |
| 1 | 10341101 |  | 355.08 | 326.03 | 243.99 | 223.38 | 123.42 | 132.56 | -1.4443 | 0.0598 |
| 1 | 10341559 |  | 37.07 | 38.89 | 29.27 | 31.79 | 23.61 | 26.90 | -0.7223 | 0.0111 |
| 1 | 10342053 |  | 5503.59 | 2918.87 | 3910.98 | 5801.86 | 2168.99 | 3232.97 | -0.4437 | 0.0479 |
| 1 | 10342382 |  | 1718.72 | 853.22 | 923.25 | 2091.77 | 506.48 | 896.87 | -0.7637 | 0.0107 |
| 1 | 10343434 |  | 2002.08 | 939.56 | 1144.13 | 1855.47 | 690.41 | 1171.48 | -0.4498 | 0.0799 |
| 1 | 10344322 |  | 485.75 | 537.60 | 542.13 | 527.16 | 386.61 | 492.32 | -0.4906 | 0.0781 |
| 1 | 10344567 |  | 255.69 | 230.98 | 180.78 | 137.62 | 105.30 | 91.52 | -1.1817 | 0.0867 |
| 1 | 10345241 | DST | 2777.49 | 2484.39 | 2686.65 | 2520.69 | 1984.38 | 2539.38 | -0.3347 | 0.0479 |
| 1 | 10347748 | Utp14b | 914.59 | 661.76 | 688.53 | 885.55 | 424.94 | 483.23 | -0.6261 | 0.0362 |
| 1 | 10348194 | EFHD1 | 1486.84 | 1359.72 | 1444.00 | 1291.19 | 609.55 | 905.67 | -1.1655 | 0.0000 |
| 1 | 10350128 | NAV1 | 308.01 | 265.41 | 286.10 | 249.00 | 216.31 | 232.36 | -0.2999 | 0.0791 |
| 1 | 10350594 | IVNS1ABP | 4042.17 | 3834.41 | 3925.28 | 3416.71 | 2953.76 | 3700.07 | -0.3727 | 0.0261 |
| 1 | 10351525 | MPZ | 10872.97 | 7813.67 | 9883.73 | 8918.64 | 4950.37 | 7742.16 | -0.6866 | 0.0397 |
| 1 | 10351971 | FMN2 | 1004.88 | 591.25 | 681.98 | 925.49 | 392.55 | 498.73 | -0.5934 | 0.0165 |
| 1 | 10351998 |  | 403.64 | 269.57 | 289.14 | 377.79 | 116.13 | 191.05 | -1.2408 | 0.0177 |
| 1 | 10352178 | SCCPDH | 4242.66 | 2974.56 | 3395.65 | 3688.22 | 1914.08 | 2351.23 | -0.6351 | 0.0004 |
| 1 | 10354141 | LONRF2 | 260.38 | 241.83 | 195.68 | 195.05 | 175.36 | 166.36 | -0.4758 | 0.0621 |
| 1 | 10354598 | HECW2 | 922.23 | 431.43 | 591.70 | 756.21 | 327.35 | 436.44 | -0.3915 | 0.0671 |
| 1 | 10356154 | SPHKAP | 217.22 | 133.44 | 167.87 | 203.41 | 88.31 | 123.72 | -0.5879 | 0.0219 |
| 1 | 10356712 | KIF1A | 2087.75 | 1061.91 | 1213.89 | 1701.42 | 663.74 | 810.06 | -0.6896 | 0.0238 |
| 1 | 10357103 | CDH19 | 3941.63 | 2955.06 | 3798.53 | 2968.71 | 2251.09 | 3404.14 | -0.3898 | 0.0740 |

| Cluster | **Affymetrix transcript #** | | Gene Symbol | Average levels of expression (arbitrary units) | | | | | | **Fold change**  **Tx_10d_ injured /Vh_10d_ injured** | |
| --- | --- | --- | --- | --- | --- | --- | --- | --- | --- | --- | --- |
|  |  |  |  | **Vh_10d_ uninjured** | **Vh_10d_ injured** | **Vh_28d_ injured** | **Tx_10d_ uninjured** | **Tx_10d_ injured** | **Tx_28d_ injured** | **Log (2) Fold change** | **Adj. p-value** |
| 1 | | 10358091 | NAV1 | 768.47 | 763.81 | 857.91 | 673.63 | 558.70 | 595.33 | -0.4514 | 0.0115 |
| 1 | | 10359235 | RASAL2 | 2144.47 | 1815.60 | 1713.28 | 1827.28 | 825.54 | 1398.93 | -1.1493 | 0.0000 |
| 1 | | 10359754 | MPZL1 | 677.39 | 553.95 | 552.95 | 574.87 | 429.98 | 533.50 | -0.3583 | 0.0940 |
| 1 | | 10360506 | AKT3 | 4342.82 | 3175.83 | 3405.09 | 3575.47 | 2514.59 | 2986.74 | -0.3387 | 0.0339 |
| 1 | | 10361007 | SMYD2 | 537.07 | 533.02 | 511.07 | 510.75 | 421.08 | 546.12 | -0.3398 | 0.0244 |
| 1 | | 10362596 | FYN | 1784.38 | 1590.61 | 1498.58 | 1472.39 | 970.16 | 1143.91 | -0.7190 | 0.0035 |
| 1 | | 10362717 | WASF1 | 433.39 | 492.98 | 364.99 | 358.21 | 252.88 | 275.84 | -0.9732 | 0.0019 |
| 1 | | 10363392 | DNAJB12 | 578.21 | 561.66 | 539.57 | 560.51 | 435.80 | 457.03 | -0.3670 | 0.0611 |
| 1 | | 10363676 | CTNNA3 | 1542.05 | 594.27 | 862.62 | 1336.74 | 334.74 | 677.58 | -0.8340 | 0.0108 |
| 1 | | 10363696 |  | 1522.32 | 699.79 | 984.18 | 1287.40 | 350.07 | 825.64 | -1.0008 | 0.0044 |
| 1 | | 10365601 | GNPTAB | 806.73 | 869.92 | 683.99 | 810.96 | 659.80 | 696.76 | -0.4009 | 0.0624 |
| 1 | | 10365891 | TMCC3 | 630.07 | 343.10 | 486.20 | 581.35 | 435.77 | 487.57 | 0.3485 | 0.0641 |
| 1 | | 10367600 | ESR1 | 608.30 | 543.69 | 491.79 | 571.24 | 410.54 | 383.91 | -0.4058 | 0.0079 |
| 1 | | 10367734 | UST | 354.13 | 373.12 | 292.38 | 285.66 | 231.07 | 278.56 | -0.6965 | 0.0056 |
| 1 | | 10367772 | SAMD5 | 538.79 | 446.12 | 386.65 | 463.58 | 355.65 | 360.71 | -0.3280 | 0.0822 |
| 1 | | 10367843 | UTRN | 5491.29 | 3551.30 | 4181.54 | 4877.38 | 2558.51 | 3558.61 | -0.4902 | 0.0156 |
| 1 | | 10367982 | GPR126 | 1705.96 | 1395.31 | 1439.18 | 1521.12 | 927.05 | 1267.00 | -0.5906 | 0.0034 |
| 1 | | 10368585 | NKAIN2 | 2700.13 | 1540.98 | 1946.33 | 2071.35 | 904.27 | 1309.20 | -0.7635 | 0.0040 |
| 1 | | 10369750 | Gm10118 | 1214.36 | 722.95 | 890.34 | 1144.99 | 325.61 | 678.28 | -1.1600 | 0.0012 |
| 1 | | 10369752 | LRRTM3 | 1899.39 | 973.08 | 1364.87 | 1387.04 | 570.03 | 1041.93 | -0.7678 | 0.0062 |
| 1 | | 10369779 | ADO | 727.33 | 691.99 | 668.36 | 707.72 | 536.19 | 592.69 | -0.3720 | 0.0345 |
| 1 | | 10370766 | GAMT | 533.11 | 605.83 | 607.43 | 596.44 | 445.96 | 463.18 | -0.4491 | 0.0489 |
| 1 | | 10371770 | GAS2L3 | 7818.33 | 6304.97 | 6061.50 | 6335.73 | 3721.66 | 4813.05 | -0.7732 | 0.0023 |
| 1 | | 10372094 |  | 59.04 | 60.62 | 62.25 | 61.24 | 49.31 | 57.61 | -0.3040 | 0.0792 |
| 1 | | 10373826 | GAL3ST1 | 482.86 | 436.87 | 443.48 | 448.77 | 273.72 | 369.90 | -0.6704 | 0.0161 |
| 1 | | 10376950 | PMP22 | 16889.88 | 12789.18 | 15301.45 | 16331.37 | 10201.91 | 13515.14 | -0.3371 | 0.0710 |
| 1 | | 10379262 | NF1 (includes EG:18015) | 2827.68 | 2298.24 | 2505.61 | 2410.84 | 1790.65 | 2234.06 | -0.3591 | 0.0150 |
| 1 | | 10380341 | SPAG9 | 2741.28 | 2477.86 | 2562.00 | 2519.78 | 1830.45 | 1996.88 | -0.4356 | 0.0122 |
| 1 | | 10381154 | CNP | 2742.75 | 2197.55 | 2434.49 | 2410.51 | 1196.22 | 2493.68 | -0.8827 | 0.0005 |
| 1 | | 10382300 | MAP2K6 | 591.64 | 394.86 | 422.37 | 509.30 | 255.74 | 364.39 | -0.6314 | 0.0048 |
| 1 | | 10382409 | KIF19 | 694.64 | 316.39 | 403.17 | 568.67 | 210.88 | 283.19 | -0.5886 | 0.0520 |
| 1 | | 10383395 | SLC25A10 | 329.68 | 336.21 | 310.32 | 328.95 | 272.88 | 287.31 | -0.3012 | 0.0348 |
| 1 | | 10383731 | SMTN | 1959.37 | 950.60 | 1272.60 | 1560.88 | 552.41 | 742.01 | -0.7773 | 0.0349 |
| 1 | | 10384150 | PURB | 1966.35 | 1838.07 | 1951.12 | 1974.86 | 1352.57 | 1666.56 | -0.4289 | 0.0823 |
| 1 | | 10384622 | EHBP1 | 1961.12 | 1356.70 | 1468.65 | 1754.47 | 1043.63 | 1522.73 | -0.3819 | 0.0697 |

| Cluster | **Affymetrix transcript #** | Gene Symbol | Average levels of expression (arbitrary units) | | | | | | **Fold change**  **Tx_10d_ injured /Vh_10d_ injured** | |
| --- | --- | --- | --- | --- | --- | --- | --- | --- | --- | --- |
|  |  |  | **Vh_10d_ uninjured** | **Vh_10d_ injured** | **Vh_28d_ injured** | **Tx_10d_ uninjured** | **Tx_10d_ injured** | **Tx_28d_ injured** | **Log (2) Fold change** | **Adj. p-value** |
| 1 | 10388254 | ASPA | 2983.62 | 2276.34 | 3361.00 | 2481.16 | 1634.81 | 3062.55 | -0.4680 | 0.0081 |
| 1 | 10389581 | YPEL2 | 396.62 | 419.62 | 401.81 | 385.25 | 254.89 | 304.40 | -0.7164 | 0.0071 |
| 1 | 10393668 | AATK | 707.23 | 561.78 | 613.84 | 538.56 | 389.93 | 488.46 | -0.5375 | 0.0980 |
| 1 | 10394789 | B430203G13Rik | 302.07 | 297.23 | 288.00 | 292.16 | 239.56 | 265.56 | -0.3168 | 0.0997 |
| 1 | 10394990 | MBOAT2 | 2203.52 | 1776.33 | 2155.92 | 1773.83 | 1226.63 | 1961.72 | -0.5311 | 0.0646 |
| 1 | 10396671 | PLEKHG3 | 262.94 | 238.43 | 242.36 | 256.84 | 189.41 | 218.68 | -0.3385 | 0.0392 |
| 1 | 10396712 | FUT8 | 2222.84 | 1185.94 | 1616.58 | 1976.21 | 783.36 | 1108.61 | -0.5950 | 0.0034 |
| 1 | 10399421 | MYCN | 188.56 | 201.06 | 193.76 | 194.73 | 160.79 | 151.17 | -0.3249 | 0.0663 |
| 1 | 10401891 | STON2 | 595.30 | 599.77 | 445.02 | 539.34 | 374.46 | 371.54 | -0.6893 | 0.0046 |
| 1 | 10402318 | PRIMA1 | 314.64 | 275.38 | 276.44 | 295.25 | 211.49 | 256.13 | -0.3867 | 0.0792 |
| 1 | 10402473 | CLMN | 612.69 | 437.22 | 483.65 | 571.92 | 279.36 | 429.53 | -0.6430 | 0.0295 |
| 1 | 10403413 | IDI1 | 965.83 | 270.10 | 236.75 | 862.39 | 133.61 | 147.50 | -0.9419 | 0.0366 |
| 1 | 10403842 | ELMO1 | 761.56 | 742.18 | 639.79 | 653.98 | 508.04 | 619.09 | -0.5518 | 0.0047 |
| 1 | 10406176 | SLC9A3 (includes EG:105243) | 634.20 | 390.00 | 208.30 | 415.96 | 160.62 | 188.13 | -1.2529 | 0.0314 |
| 1 | 10407072 | ELOVL7 | 3535.19 | 1263.17 | 1907.29 | 3285.85 | 757.43 | 884.03 | -0.7405 | 0.0417 |
| 1 | 10410317 | Zfp459 | 83.94 | 91.71 | 79.23 | 83.85 | 62.85 | 67.03 | -0.5607 | 0.0839 |
| 1 | 10410695 | RHOBTB3 | 3292.68 | 1253.40 | 1662.56 | 2509.89 | 838.60 | 1204.36 | -0.5743 | 0.0398 |
| 1 | 10411126 | JMY | 896.16 | 751.64 | 686.49 | 820.19 | 608.90 | 673.72 | -0.3046 | 0.0590 |
| 1 | 10411332 | HMGCR | 1844.93 | 919.88 | 738.76 | 1762.62 | 552.96 | 519.02 | -0.7277 | 0.0538 |
| 1 | 10411395 | RGNEF | 543.43 | 493.03 | 527.84 | 513.52 | 406.10 | 551.67 | -0.2822 | 0.0468 |
| 1 | 10412466 | HMGCS1 | 4932.30 | 1683.99 | 1924.54 | 4234.03 | 949.60 | 1139.32 | -0.8008 | 0.0077 |
| 1 | 10414417 | PELI2 | 1110.25 | 1104.48 | 910.32 | 1077.97 | 586.33 | 743.25 | -0.9430 | 0.0005 |
| 1 | 10414427 | Gm6498 | 435.94 | 426.39 | 336.93 | 353.12 | 239.74 | 255.43 | -0.8137 | 0.0007 |
| 1 | 10414623 | ARHGEF40 | 444.22 | 458.00 | 468.41 | 447.19 | 356.04 | 453.16 | -0.3686 | 0.0181 |
| 1 | 10415132 | CMTM5 | 2274.09 | 1278.57 | 2019.47 | 2288.69 | 870.01 | 1525.56 | -0.5549 | 0.0813 |
| 1 | 10415678 | CAB39L | 1440.01 | 839.15 | 1117.25 | 1165.68 | 610.04 | 885.79 | -0.4582 | 0.0740 |
| 1 | 10415784 | TRIM13 | 1133.95 | 1292.03 | 1284.62 | 1244.98 | 800.91 | 1073.77 | -0.7009 | 0.0617 |
| 1 | 10418991 | FAM190B | 5199.69 | 3870.66 | 4305.93 | 4641.92 | 3202.38 | 4013.93 | -0.2732 | 0.0432 |
| 1 | 10419676 | RAB2B | 1017.72 | 1013.37 | 935.00 | 810.75 | 694.91 | 983.72 | -0.5347 | 0.0349 |
| 1 | 10420846 | FZD3 | 2853.48 | 1408.85 | 1558.59 | 2278.75 | 819.13 | 1147.30 | -0.7877 | 0.0131 |
| 1 | 10423902 | ZFPM2 | 130.16 | 127.51 | 114.75 | 124.08 | 107.09 | 119.80 | -0.2535 | 0.0807 |
| 1 | 10425354 | MGAT3 | 513.64 | 383.86 | 455.53 | 533.03 | 296.36 | 409.29 | -0.3714 | 0.0209 |
| 1 | 10426467 | TMEM117 | 460.18 | 264.15 | 369.09 | 413.86 | 182.76 | 216.48 | -0.5215 | 0.0727 |
| 1 | 10428698 | SNTB1 | 869.51 | 519.97 | 648.51 | 836.59 | 358.39 | 524.10 | -0.5378 | 0.0505 |
| 1 | 10428998 | ASAP1 | 840.36 | 879.58 | 726.02 | 751.51 | 708.59 | 629.00 | -0.3169 | 0.0551 |

| Cluster | **Affymetrix transcript #** | Gene Symbol | Average levels of expression (arbitrary units) | | | | | | **Fold change**  **Tx_10d_ injured /Vh_10d_ injured** | |
| --- | --- | --- | --- | --- | --- | --- | --- | --- | --- | --- |
|  |  |  | **Vh_10d_ uninjured** | **Vh_10d_ injured** | **Vh_28d_ injured** | **Tx_10d_ uninjured** | **Tx_10d_ injured** | **Tx_28d_ injured** | **Log (2) Fold change** | **Adj. p-value** |
| 1 | 10430113 | ARHGAP39 | 720.90 | 540.96 | 567.85 | 512.27 | 382.41 | 503.40 | -0.5102 | 0.0838 |
| 1 | 10431051 | SCUBE1 | 750.95 | 522.30 | 667.75 | 533.75 | 360.57 | 320.76 | -0.5375 | 0.0559 |
| 1 | 10432372 | DHH | 379.01 | 355.60 | 359.40 | 365.17 | 272.23 | 311.90 | -0.3865 | 0.0392 |
| 1 | 10434291 | B3GNT5 | 840.44 | 288.14 | 273.79 | 687.82 | 204.50 | 175.46 | -0.4864 | 0.0066 |
| 1 | 10434758 | ST6GAL1 | 1274.10 | 1085.88 | 1392.39 | 1049.11 | 754.81 | 1035.53 | -0.5195 | 0.0583 |
| 1 | 10440216 | EPHA6 | 340.69 | 150.82 | 179.88 | 252.45 | 86.35 | 82.57 | -0.8086 | 0.0447 |
| 1 | 10441565 | RPS6KA2 | 343.58 | 278.41 | 290.69 | 308.60 | 184.72 | 254.96 | -0.5867 | 0.0034 |
| 1 | 10441718 | PARK2 | 438.70 | 306.17 | 366.99 | 373.78 | 236.33 | 287.31 | -0.3773 | 0.0592 |
| 1 | 10443131 | ITPR3 | 957.43 | 745.62 | 793.17 | 845.66 | 533.93 | 654.81 | -0.4836 | 0.0860 |
| 1 | 10444431 | PRRT1 | 220.21 | 202.01 | 215.70 | 220.41 | 167.86 | 214.44 | -0.2694 | 0.0946 |
| 1 | 10446063 | TNFAIP8L1 | 257.97 | 213.78 | 244.34 | 267.65 | 159.88 | 197.81 | -0.4258 | 0.0914 |
| 1 | 10447708 | QKI | 8633.55 | 6641.09 | 7075.96 | 7481.39 | 5254.21 | 6381.06 | -0.3343 | 0.0131 |
| 1 | 10447732 | PACRG | 271.18 | 275.35 | 254.99 | 249.21 | 184.84 | 219.39 | -0.5704 | 0.0416 |
| 1 | 10448967 | SOX8 | 302.07 | 293.63 | 296.81 | 317.95 | 234.59 | 312.66 | -0.3252 | 0.0728 |
| 1 | 10449312 |  | 201.26 | 199.62 | 198.21 | 172.97 | 141.49 | 184.00 | -0.5142 | 0.0148 |
| 1 | 10450622 | ATAT1 | 494.49 | 520.05 | 445.19 | 420.46 | 378.09 | 384.62 | -0.4642 | 0.0131 |
| 1 | 10451167 | TMEM63B | 514.69 | 526.71 | 447.44 | 486.56 | 419.38 | 421.32 | -0.3360 | 0.0666 |
| 1 | 10451763 | SATB1 | 2188.02 | 1247.78 | 1335.43 | 1986.43 | 897.67 | 1035.94 | -0.4713 | 0.0398 |
| 1 | 10454851 | CXXC5 | 525.10 | 454.56 | 487.73 | 498.20 | 383.16 | 463.53 | -0.2507 | 0.0734 |
| 1 | 10455769 | CSNK1G3 | 1307.04 | 1201.23 | 1133.99 | 1218.98 | 896.38 | 1089.51 | -0.4216 | 0.0095 |
| 1 | 10456140 | SH3TC2 | 398.11 | 255.49 | 269.04 | 332.23 | 164.35 | 218.34 | -0.6275 | 0.0108 |
| 1 | 10457022 | MBP | 4592.36 | 2499.45 | 3665.91 | 4035.87 | 1577.56 | 2579.47 | -0.6734 | 0.0127 |
| 1 | 10457475 | ABHD3 | 465.03 | 527.12 | 424.06 | 374.09 | 253.63 | 301.36 | -1.0517 | 0.0000 |
| 1 | 10462035 | Ldhb | 2933.22 | 1587.28 | 1895.44 | 2913.04 | 1088.07 | 1268.90 | -0.5419 | 0.0108 |
| 1 | 10462333 | CDC37L1 | 2013.09 | 1409.40 | 1515.74 | 1699.94 | 1143.92 | 1382.70 | -0.3026 | 0.0829 |
| 1 | 10462587 | STAMBPL1 | 585.08 | 392.14 | 461.70 | 572.36 | 470.33 | 446.95 | 0.2576 | 0.0884 |
| 1 | 10463430 | SEMA4G | 381.62 | 291.47 | 345.13 | 321.62 | 192.09 | 227.32 | -0.6028 | 0.0093 |
| 1 | 10463457 | LZTS2 | 557.15 | 486.65 | 481.65 | 550.47 | 409.16 | 474.19 | -0.2495 | 0.0614 |
| 1 | 10464283 | Gm10007 | 476.74 | 382.53 | 361.58 | 363.15 | 267.52 | 293.07 | -0.5120 | 0.0131 |
| 1 | 10464642 | CARNS1 | 1061.48 | 535.97 | 764.27 | 918.08 | 353.53 | 539.59 | -0.6093 | 0.0407 |
| 1 | 10465844 | ASRGL1 | 1923.67 | 1025.23 | 1464.05 | 1486.92 | 758.46 | 967.14 | -0.4391 | 0.0492 |
| 1 | 10466410 | PSAT1 | 710.27 | 673.48 | 530.70 | 557.67 | 494.36 | 566.24 | -0.4374 | 0.0534 |
| 1 | 10467425 | SORBS1 | 2529.23 | 2335.52 | 2418.43 | 2179.34 | 1758.61 | 2273.39 | -0.4281 | 0.0175 |
| 1 | 10467637 | ARHGAP19 | 4357.09 | 2783.15 | 3030.98 | 3470.13 | 1331.73 | 2203.54 | -1.0847 | 0.0002 |
| 1 | 10468030 | PDZD7 | 318.98 | 346.61 | 321.26 | 333.76 | 261.70 | 291.18 | -0.4019 | 0.0305 |
| 1 | 10468452 | SORCS1 | 2537.26 | 1601.86 | 1695.42 | 2140.66 | 1043.63 | 1452.44 | -0.6196 | 0.0189 |

| Cluster | **Affymetrix transcript #** | Gene Symbol | Average levels of expression (arbitrary units) | | | | | | **Fold change**  **Tx_10d_ injured /Vh_10d_ injured** | |
| --- | --- | --- | --- | --- | --- | --- | --- | --- | --- | --- |
|  |  |  | **Vh_10d_ uninjured** | **Vh_10d_ injured** | **Vh_28d_ injured** | **Tx_10d_ uninjured** | **Tx_10d_ injured** | **Tx_28d_ injured** | **Log (2) Fold change** | **Adj. p-value** |
| 1 | 10469046 | PHYH | 1458.70 | 1479.00 | 1433.76 | 1586.96 | 1136.65 | 1322.03 | -0.3800 | 0.0261 |
| 1 | 10469110 | USP6NL | 4605.90 | 3273.88 | 3699.42 | 3898.04 | 2075.40 | 2904.85 | -0.6614 | 0.0007 |
| 1 | 10469312 | PTER | 822.00 | 603.04 | 567.91 | 796.41 | 445.37 | 403.74 | -0.4348 | 0.0834 |
| 1 | 10471953 | ACVR2A | 1600.15 | 1590.46 | 1270.34 | 1485.06 | 1133.78 | 1021.92 | -0.4831 | 0.0447 |
| 1 | 10472097 | FMNL2 | 2837.52 | 1972.52 | 2417.15 | 2637.47 | 1582.20 | 2185.49 | -0.3259 | 0.0726 |
| 1 | 10472162 | GPD2 | 962.00 | 643.17 | 610.54 | 970.31 | 516.17 | 572.42 | -0.3191 | 0.0515 |
| 1 | 10474419 | LGR4 | 962.43 | 516.78 | 473.86 | 657.57 | 366.70 | 396.36 | -0.4863 | 0.0317 |
| 1 | 10475218 | Stard9 (mouse) | 1435.14 | 1440.05 | 1417.26 | 1231.93 | 1033.14 | 1333.99 | -0.4826 | 0.0486 |
| 1 | 10475226 | Stard9 (mouse) | 756.76 | 827.32 | 730.58 | 770.74 | 557.30 | 744.73 | -0.5696 | 0.0020 |
| 1 | 10475544 | SEMA6D | 2905.17 | 2866.45 | 2553.23 | 2515.09 | 1768.03 | 3196.29 | -0.7035 | 0.0020 |
| 1 | 10476301 | SMOX | 676.46 | 506.08 | 499.17 | 544.05 | 354.31 | 438.69 | -0.5151 | 0.0414 |
| 1 | 10476538 | BTBD3 | 926.67 | 896.57 | 905.36 | 950.44 | 760.92 | 797.57 | -0.2385 | 0.0822 |
| 1 | 10480139 | C1QL3 | 1633.95 | 1222.32 | 1363.93 | 1451.87 | 859.88 | 677.80 | -0.5051 | 0.0127 |
| 1 | 10482762 | IDI1 | 1071.90 | 306.03 | 264.53 | 1006.09 | 145.40 | 157.75 | -1.0089 | 0.0143 |
| 1 | 10482772 | NR4A2 | 1491.09 | 950.21 | 1199.80 | 1429.03 | 665.82 | 784.56 | -0.5122 | 0.0471 |
| 1 | 10483381 | STK39 | 3464.01 | 2974.18 | 2514.23 | 2897.85 | 1711.88 | 2049.10 | -0.7967 | 0.0000 |
| 1 | 10485597 | DEPDC7 | 1119.80 | 1034.95 | 956.06 | 1094.75 | 482.79 | 1014.08 | -1.0906 | 0.0004 |
| 1 | 10487139 | SHC4 | 2807.94 | 1133.81 | 1456.73 | 2280.27 | 648.96 | 811.52 | -0.8016 | 0.0027 |
| 1 | 10487154 | SECISBP2L | 6604.43 | 3692.85 | 4819.21 | 6091.74 | 2301.31 | 3743.94 | -0.6928 | 0.0020 |
| 1 | 10487441 | MAL | 4587.11 | 2646.26 | 3598.88 | 4791.23 | 1890.52 | 2878.83 | -0.4867 | 0.0197 |
| 1 | 10488678 | DUSP15 | 2089.32 | 687.31 | 1085.01 | 1277.67 | 337.19 | 487.82 | -1.0197 | 0.0097 |
| 1 | 10488709 | 8430427H17Rik | 355.65 | 379.61 | 365.52 | 354.05 | 282.77 | 321.92 | -0.4242 | 0.0354 |
| 1 | 10490061 | BCAS1 | 1816.55 | 886.63 | 1232.35 | 1482.97 | 459.80 | 864.35 | -0.9484 | 0.0018 |
| 1 | 10490815 |  | 53.37 | 58.34 | 57.91 | 58.41 | 44.52 | 55.40 | -0.3900 | 0.0842 |
| 1 | 10490826 | ZBTB10 | 1042.62 | 971.71 | 950.05 | 972.04 | 741.44 | 858.77 | -0.3901 | 0.0311 |
| 1 | 10492231 | MED12L | 256.83 | 248.28 | 236.18 | 252.84 | 202.09 | 209.68 | -0.2957 | 0.0112 |
| 1 | 10492341 | ARHGEF26 | 512.07 | 435.13 | 365.80 | 415.45 | 292.24 | 327.02 | -0.5817 | 0.0210 |
| 1 | 10492355 | MME | 4662.56 | 2112.95 | 3545.39 | 4159.43 | 1212.18 | 1917.95 | -0.7607 | 0.0160 |
| 1 | 10492522 | IQCJ-SCHIP1 | 1046.81 | 951.20 | 969.34 | 926.38 | 746.28 | 887.48 | -0.3484 | 0.0929 |
| 1 | 10492590 | PPM1L | 870.17 | 529.75 | 550.60 | 773.98 | 322.60 | 440.54 | -0.7193 | 0.0093 |
| 1 | 10492864 | SH3D19 | 1421.01 | 1289.91 | 1279.34 | 1332.92 | 841.87 | 1149.14 | -0.6183 | 0.0012 |
| 1 | 10494322 | ANP32E | 1942.67 | 2026.22 | 1740.58 | 1805.12 | 1669.27 | 1575.28 | -0.2822 | 0.0813 |
| 1 | 10495193 | KCNA2 | 1600.04 | 1099.17 | 1804.09 | 1344.77 | 777.77 | 2001.08 | -0.5040 | 0.0479 |
| 1 | 10495197 | KCNA2 | 574.22 | 446.60 | 627.49 | 478.72 | 310.24 | 682.87 | -0.5348 | 0.0997 |
| 1 | 10495285 | SORT1 | 1238.39 | 879.40 | 916.02 | 1105.28 | 667.91 | 955.36 | -0.4018 | 0.0813 |

| Cluster | **Affymetrix transcript #** | Gene Symbol | Average levels of expression (arbitrary units) | | | | | | **Fold change**  **Tx_10d_ injured /Vh_10d_ injured** | |
| --- | --- | --- | --- | --- | --- | --- | --- | --- | --- | --- |
|  |  |  | **Vh_10d_ uninjured** | **Vh_10d_ injured** | **Vh_28d_ injured** | **Tx_10d_ uninjured** | **Tx_10d_ injured** | **Tx_28d_ injured** | **Log (2) Fold change** | **Adj. p-value** |
| 1 | 10495993 | ELOVL6 | 3261.53 | 1192.10 | 1451.06 | 3474.64 | 599.99 | 799.16 | -0.9811 | 0.0016 |
| 1 | 10496110 | PAPSS1 | 900.55 | 650.93 | 734.71 | 735.46 | 414.55 | 571.15 | -0.6510 | 0.0044 |
| 1 | 10497253 | PMP2 (includes EG:100334189) | 3274.26 | 3371.10 | 3146.13 | 2939.00 | 2094.23 | 1063.02 | -0.6738 | 0.0846 |
| 1 | 10497773 | MCCC1 | 593.07 | 514.44 | 524.24 | 632.04 | 386.39 | 469.46 | -0.4127 | 0.0445 |
| 1 | 10497920 | Ankrd50 | 971.24 | 676.01 | 689.58 | 759.37 | 487.34 | 572.05 | -0.4726 | 0.0140 |
| 1 | 10497944 | MFSD8 | 512.69 | 512.58 | 442.63 | 442.76 | 361.00 | 384.31 | -0.5051 | 0.0166 |
| 1 | 10499045 | TRIM2 | 428.90 | 305.90 | 305.40 | 276.84 | 234.11 | 239.19 | -0.3892 | 0.0980 |
| 1 | 10499113 | PRSS48 | 723.98 | 773.87 | 794.40 | 744.70 | 458.08 | 720.08 | -0.7651 | 0.0002 |
| 1 | 10499431 | SYT11 | 1186.40 | 840.79 | 1015.90 | 840.57 | 517.62 | 748.78 | -0.7090 | 0.0207 |
| 1 | 10499483 | FDPS | 1045.46 | 464.10 | 434.68 | 954.59 | 316.72 | 361.39 | -0.5447 | 0.0438 |
| 1 | 10500283 | APH1A | 268.84 | 234.03 | 303.05 | 248.02 | 178.98 | 230.18 | -0.3921 | 0.0791 |
| 1 | 10500434 | BCL9 | 836.67 | 743.89 | 818.99 | 873.61 | 597.22 | 643.66 | -0.3235 | 0.0813 |
| 1 | 10501963 | UGT8 | 7188.52 | 3620.06 | 5351.07 | 6832.47 | 1699.54 | 3306.10 | -1.1028 | 0.0004 |
| 1 | 10503952 | IFNK | 569.24 | 228.41 | 341.84 | 529.53 | 147.59 | 314.72 | -0.6495 | 0.0507 |
| 1 | 10504234 | UNC13B | 354.33 | 381.66 | 342.59 | 351.01 | 277.71 | 292.03 | -0.4600 | 0.0165 |
| 1 | 10505568 | FRMD3 | 2167.02 | 1153.06 | 1506.79 | 1842.43 | 517.25 | 1013.46 | -1.1395 | 0.0000 |
| 1 | 10506883 | RAB3B | 246.07 | 191.65 | 231.55 | 165.24 | 138.66 | 215.91 | -0.4650 | 0.0366 |
| 1 | 10507379 | ZSWIM5 | 118.13 | 122.67 | 123.75 | 126.98 | 93.88 | 108.93 | -0.3904 | 0.0050 |
| 1 | 10507539 | ELOVL1 | 4594.03 | 2529.92 | 2933.95 | 3959.05 | 1669.20 | 2076.18 | -0.5951 | 0.0189 |
| 1 | 10507635 | CLDN19 | 2277.55 | 1546.79 | 1753.43 | 2051.60 | 687.06 | 1211.81 | -1.1804 | 0.0003 |
| 1 | 10507908 | FHL3 | 439.83 | 425.19 | 408.23 | 323.14 | 297.68 | 305.55 | -0.5152 | 0.0044 |
| 1 | 10510643 | PLEKHG5 | 290.73 | 325.44 | 302.11 | 320.17 | 237.57 | 275.68 | -0.4645 | 0.0207 |
| 1 | 10513061 | CTNNAL1 | 6702.32 | 4082.67 | 4692.13 | 5875.19 | 2007.47 | 3686.05 | -1.0325 | 0.0000 |
| 1 | 10513082 | TMEM245 | 1067.17 | 687.76 | 763.85 | 905.03 | 472.58 | 665.89 | -0.5378 | 0.0767 |
| 1 | 10513608 | ALAD | 668.31 | 462.70 | 479.93 | 636.66 | 369.03 | 413.78 | -0.3273 | 0.0503 |
| 1 | 10513869 | MEGF9 | 2376.19 | 1619.29 | 1626.67 | 2086.43 | 1028.84 | 1549.49 | -0.6686 | 0.0253 |
| 1 | 10514520 | Cyp2j9 | 2090.99 | 1639.64 | 2177.61 | 1515.96 | 1032.09 | 1882.78 | -0.6583 | 0.0107 |
| 1 | 10514576 | KANK4 | 1421.24 | 767.56 | 775.51 | 1128.42 | 482.49 | 770.13 | -0.6861 | 0.0505 |
| 1 | 10515277 | LURAP1 | 336.22 | 268.19 | 313.28 | 337.94 | 215.82 | 258.94 | -0.3197 | 0.0663 |
| 1 | 10516229 | UTP11L | 1617.02 | 1379.26 | 1384.87 | 1536.22 | 1038.65 | 1206.53 | -0.4104 | 0.0240 |
| 1 | 10517364 | C1orf130 | 1236.09 | 499.63 | 912.08 | 902.84 | 291.30 | 594.10 | -0.7867 | 0.0390 |
| 1 | 10518428 | CLCN6 | 933.64 | 672.80 | 685.38 | 764.99 | 480.93 | 610.82 | -0.4902 | 0.0870 |
| 1 | 10519028 | PRDM16 | 593.82 | 595.81 | 644.28 | 562.79 | 416.27 | 574.93 | -0.5222 | 0.0092 |
| 1 | 10519203 | TMEM88B | 189.32 | 182.43 | 193.61 | 169.03 | 145.50 | 172.64 | -0.3268 | 0.0481 |
| 1 | 10522368 | NIPAL1 | 494.28 | 521.51 | 375.15 | 512.61 | 347.80 | 350.18 | -0.5994 | 0.0364 |

| Cluster | **Affymetrix transcript #** | Gene Symbol | Average levels of expression (arbitrary units) | | | | | | **Fold change**  **Tx_10d_ injured /Vh_10d_ injured** | |
| --- | --- | --- | --- | --- | --- | --- | --- | --- | --- | --- |
|  |  |  | **Vh_10d_ uninjured** | **Vh_10d_ injured** | **Vh_28d_ injured** | **Tx_10d_ uninjured** | **Tx_10d_ injured** | **Tx_28d_ injured** | **Log (2) Fold change** | **Adj. p-value** |
| 1 | 10523231 | ART3 | 4763.76 | 2943.85 | 3516.58 | 4584.86 | 1844.02 | 3674.94 | -0.6678 | 0.0080 |
| 1 | 10525733 | SETD8 | 1627.01 | 1530.89 | 1293.30 | 1765.84 | 1123.97 | 1203.20 | -0.4497 | 0.0641 |
| 1 | 10526014 | SFSWAP | 856.73 | 734.77 | 721.76 | 821.05 | 572.85 | 671.42 | -0.3589 | 0.0422 |
| 1 | 10527051 | SDK1 | 533.95 | 302.28 | 377.62 | 412.80 | 239.09 | 275.84 | -0.3329 | 0.0822 |
| 1 | 10527430 | ARPC1A | 1616.31 | 1468.12 | 1499.47 | 1509.77 | 1092.34 | 1304.78 | -0.4257 | 0.0245 |
| 1 | 10527920 | CYP51A1 | 1650.50 | 517.47 | 502.45 | 1663.46 | 343.23 | 381.54 | -0.5926 | 0.0693 |
| 1 | 10528227 | GNAI1 | 2881.97 | 1856.04 | 2008.16 | 3015.25 | 1133.90 | 1852.05 | -0.7201 | 0.0005 |
| 1 | 10528385 | RELN | 2300.78 | 1632.94 | 1870.61 | 1862.50 | 1194.31 | 1820.41 | -0.4575 | 0.0471 |
| 1 | 10529923 | LCORL | 653.43 | 589.40 | 509.63 | 563.13 | 403.79 | 423.23 | -0.5471 | 0.0186 |
| 1 | 10530319 | ATP8A1 | 3142.66 | 1749.78 | 2323.96 | 2434.43 | 1340.98 | 1369.34 | -0.3723 | 0.0740 |
| 1 | 10530563 | FRYL | 2260.20 | 1192.22 | 1491.53 | 2280.41 | 930.01 | 1333.38 | -0.3657 | 0.0739 |
| 1 | 10531420 | CXCL11 | 466.99 | 359.13 | 395.83 | 451.37 | 257.20 | 340.13 | -0.4817 | 0.0270 |
| 1 | 10532085 | TGFBR3 | 1575.46 | 1516.11 | 1473.36 | 1346.78 | 1122.36 | 1394.86 | -0.4563 | 0.0478 |
| 1 | 10532984 | DYNLL1 | 2949.08 | 2830.28 | 2902.90 | 2894.03 | 2343.04 | 2597.95 | -0.2665 | 0.0671 |
| 1 | 10534168 | AUTS2 | 1227.79 | 779.29 | 764.85 | 1044.10 | 505.76 | 544.87 | -0.6301 | 0.0060 |
| 1 | 10535312 | RADIL | 353.29 | 251.14 | 257.00 | 319.45 | 200.46 | 239.43 | -0.3257 | 0.0505 |
| 1 | 10536297 | PPP1R9A | 1458.76 | 760.79 | 921.03 | 1228.53 | 562.13 | 788.56 | -0.4326 | 0.0348 |
| 1 | 10536494 | CAV2 | 1621.05 | 600.78 | 920.45 | 1416.38 | 784.43 | 792.93 | 0.4031 | 0.0727 |
| 1 | 10538253 | MPP6 (includes EG:35343) | 2184.44 | 2769.87 | 1779.77 | 1990.84 | 1108.91 | 1331.13 | -1.3453 | 0.0000 |
| 1 | 10539002 | RNF103 | 1065.79 | 941.74 | 902.36 | 1055.36 | 711.42 | 874.07 | -0.4037 | 0.0127 |
| 1 | 10539813 | CCDC48 | 1073.51 | 733.86 | 793.51 | 1040.01 | 509.93 | 614.72 | -0.5326 | 0.0487 |
| 1 | 10540207 |  | 720.98 | 656.67 | 501.24 | 551.10 | 398.83 | 279.46 | -0.7148 | 0.0232 |
| 1 | 10541114 | RASGEF1A | 592.10 | 360.91 | 427.33 | 471.86 | 183.70 | 244.86 | -0.9718 | 0.0027 |
| 1 | 10541496 | MFAP5 | 6513.84 | 2475.54 | 3275.90 | 6365.99 | 3641.52 | 3025.10 | 0.5943 | 0.0296 |
| 1 | 10543145 | THSD7A | 471.05 | 458.06 | 464.52 | 430.58 | 352.75 | 461.15 | -0.3820 | 0.0051 |
| 1 | 10544644 | DFNA5 | 760.40 | 910.58 | 648.55 | 757.25 | 364.64 | 503.37 | -1.3127 | 0.0001 |
| 1 | 10544875 | SCRN1 | 1917.54 | 1430.12 | 1568.42 | 1658.17 | 1056.86 | 1198.28 | -0.4351 | 0.0663 |
| 1 | 10544906 | GGCT | 1411.66 | 1157.75 | 1116.10 | 1337.72 | 902.27 | 835.77 | -0.3581 | 0.0697 |
| 1 | 10545760 | PAIP2B | 1749.26 | 1276.16 | 1473.55 | 1398.66 | 968.98 | 1232.43 | -0.3965 | 0.0234 |
| 1 | 10546476 | MAGI1 | 479.65 | 520.60 | 419.32 | 470.86 | 388.95 | 398.73 | -0.4267 | 0.0111 |
| 1 | 10547540 | MICAL3 | 888.44 | 653.70 | 653.35 | 789.53 | 397.68 | 480.80 | -0.7198 | 0.0007 |
| 1 | 10547613 | RIMKLB | 1867.69 | 628.37 | 1134.36 | 1585.52 | 300.47 | 567.25 | -1.0500 | 0.0015 |
| 1 | 10548729 | MANSC1 | 239.08 | 235.43 | 221.74 | 262.99 | 192.07 | 187.77 | -0.2946 | 0.0829 |
| 1 | 10548871 | C12orf69 | 1563.58 | 522.43 | 839.67 | 1365.42 | 307.52 | 453.97 | -0.7594 | 0.0138 |
| 1 | 10549097 | Ldhb | 1032.07 | 606.38 | 700.15 | 948.21 | 433.88 | 498.74 | -0.4894 | 0.0300 |
| 1 | 10549256 | KRAS | 4086.64 | 2822.67 | 3107.64 | 3664.76 | 2029.36 | 2580.83 | -0.4687 | 0.0040 |
| 1 | 10551365 | PRX | 860.77 | 469.00 | 769.92 | 748.53 | 282.87 | 466.08 | -0.7200 | 0.0233 |

| Cluster | **Affymetrix transcript #** | Gene Symbol | Average levels of expression (arbitrary units) | | | | | | **Fold change**  **Tx_10d_ injured /Vh_10d_ injured** | |
| --- | --- | --- | --- | --- | --- | --- | --- | --- | --- | --- |
|  |  |  | **Vh_10d_ uninjured** | **Vh_10d_ injured** | **Vh_28d_ injured** | **Tx_10d_ uninjured** | **Tx_10d_ injured** | **Tx_28d_ injured** | **Log (2) Fold change** | **Adj. p-value** |
| 1 | 10551852 | CLIP3 | 1779.54 | 1194.14 | 1238.86 | 1830.84 | 834.17 | 1111.50 | -0.5306 | 0.0258 |
| 1 | 10552262 |  | 126.06 | 125.27 | 119.82 | 114.62 | 85.99 | 99.73 | -0.5266 | 0.0613 |
| 1 | 10552982 | PLEKHA4 | 1088.98 | 761.59 | 985.18 | 877.96 | 503.29 | 868.31 | -0.6159 | 0.0369 |
| 1 | 10553004 | HSD17B14 | 267.11 | 273.41 | 270.75 | 255.14 | 200.75 | 247.61 | -0.4446 | 0.0225 |
| 1 | 10554059 |  | 588.09 | 688.31 | 646.62 | 524.23 | 291.22 | 443.00 | -1.2606 | 0.0007 |
| 1 | 10554061 | ADAMTS17 | 1086.74 | 1157.21 | 1201.46 | 1218.09 | 870.10 | 1095.29 | -0.4067 | 0.0548 |
| 1 | 10556491 | FAR1 (includes EG:100034578) | 3418.26 | 2480.24 | 2692.87 | 2894.41 | 1970.57 | 2364.23 | -0.3328 | 0.0965 |
| 1 | 10556640 | CCP110 | 388.28 | 373.27 | 308.14 | 290.82 | 294.69 | 308.96 | -0.3385 | 0.0710 |
| 1 | 10558481 | DPYSL4 | 326.26 | 270.35 | 309.37 | 269.52 | 211.77 | 307.17 | -0.3521 | 0.0399 |
| 1 | 10560043 | ZNF329 | 1261.78 | 1248.41 | 1142.35 | 1126.24 | 990.99 | 1092.02 | -0.3342 | 0.0165 |
| 1 | 10562152 | MAG | 780.21 | 678.78 | 542.37 | 552.97 | 207.73 | 394.55 | -1.6988 | 0.0000 |
| 1 | 10562223 | LGI4 | 2914.01 | 1683.49 | 2778.72 | 2496.91 | 1012.88 | 1588.59 | -0.7526 | 0.0119 |
| 1 | 10565292 | ARNT2 | 448.15 | 486.33 | 460.57 | 411.22 | 298.73 | 371.77 | -0.6998 | 0.0002 |
| 1 | 10566723 | LMO1 | 443.52 | 323.77 | 283.27 | 424.37 | 213.92 | 216.19 | -0.5984 | 0.0144 |
| 1 | 10567134 | PLEKHA7 | 396.46 | 300.47 | 361.81 | 375.49 | 195.56 | 247.93 | -0.6195 | 0.0050 |
| 1 | 10571241 | PURG | 1581.19 | 1459.09 | 1407.81 | 1463.94 | 871.41 | 1129.26 | -0.7581 | 0.0016 |
| 1 | 10571266 | PPP2CB | 2097.80 | 2180.13 | 1995.68 | 1969.44 | 1773.95 | 1902.32 | -0.2982 | 0.0733 |
| 1 | 10571371 | TUSC3 | 516.59 | 530.20 | 515.22 | 514.68 | 431.82 | 478.71 | -0.2938 | 0.0994 |
| 1 | 10571907 | MFAP3L | 579.44 | 502.71 | 510.71 | 536.25 | 342.36 | 423.66 | -0.5546 | 0.0108 |
| 1 | 10571922 | NEK1 | 4825.12 | 2418.52 | 3319.95 | 4370.24 | 1678.49 | 2755.56 | -0.5333 | 0.0486 |
| 1 | 10574350 | MMP15 | 786.74 | 645.22 | 658.80 | 693.44 | 377.18 | 583.40 | -0.7957 | 0.0045 |
| 1 | 10575052 | CDH1 | 1959.94 | 826.95 | 882.36 | 1407.77 | 437.33 | 608.64 | -0.9049 | 0.0026 |
| 1 | 10577910 | FNTA | 1278.99 | 1326.76 | 1071.08 | 1085.79 | 702.49 | 951.85 | -0.9155 | 0.0000 |
| 1 | 10578572 | STOX2 | 2930.30 | 1945.41 | 1710.96 | 2390.44 | 1106.27 | 1442.18 | -0.7967 | 0.0004 |
| 1 | 10578810 | CLCN3 | 1217.72 | 886.59 | 856.81 | 967.34 | 600.07 | 755.01 | -0.5666 | 0.0059 |
| 1 | 10580765 | PLLP | 2921.37 | 1691.70 | 2359.17 | 2480.80 | 810.87 | 1514.79 | -1.0820 | 0.0033 |
| 1 | 10581824 | FA2H | 3494.15 | 1557.34 | 2166.05 | 3190.16 | 683.00 | 1436.86 | -1.3090 | 0.0033 |
| 1 | 10584741 | SLC37A4 | 207.37 | 198.77 | 209.11 | 196.87 | 160.76 | 183.52 | -0.3196 | 0.0829 |
| 1 | 10585146 | TMPRSS5 | 374.22 | 365.57 | 375.40 | 379.66 | 267.18 | 353.43 | -0.4524 | 0.0144 |
| 1 | 10586017 | UACA | 2895.52 | 2064.32 | 2392.93 | 2704.49 | 1699.43 | 2291.56 | -0.2831 | 0.0801 |
| 1 | 10587446 | MYO6 | 998.21 | 719.19 | 810.79 | 888.71 | 563.88 | 656.73 | -0.3433 | 0.0972 |
| 1 | 10587988 | GK5 | 452.85 | 462.57 | 464.79 | 418.26 | 290.18 | 422.83 | -0.6826 | 0.0300 |
| 1 | 10589368 | PLXNB1 | 637.06 | 391.46 | 453.08 | 574.04 | 308.23 | 419.10 | -0.3557 | 0.0821 |
| 1 | 10591967 | JAM3 | 1074.26 | 742.43 | 859.56 | 844.28 | 450.08 | 571.61 | -0.7246 | 0.0299 |
| 1 | 10593927 | SCAMP5 | 835.98 | 660.55 | 719.89 | 704.61 | 431.06 | 622.54 | -0.6151 | 0.0126 |
| 1 | 10595094 | MLIP | 3167.49 | 1590.03 | 1854.73 | 2723.73 | 891.78 | 1127.27 | -0.8378 | 0.0005 |
| 1 | 10595298 | FILIP1 | 3294.22 | 1444.89 | 1700.79 | 3094.32 | 1045.97 | 1199.52 | -0.4548 | 0.0944 |

| Cluster | **Affymetrix transcript #** | Gene Symbol | Average levels of expression (arbitrary units) | | | | | | **Fold change**  **Tx_10d_ injured /Vh_10d_ injured** | |
| --- | --- | --- | --- | --- | --- | --- | --- | --- | --- | --- |
|  |  |  | **Vh_10d_ uninjured** | **Vh_10d_ injured** | **Vh_28d_ injured** | **Tx_10d_ uninjured** | **Tx_10d_ injured** | **Tx_28d_ injured** | **Log (2) Fold change** | **Adj. p-value** |
| 1 | 10598872 | RBM10 | 414.17 | 456.97 | 445.75 | 463.73 | 370.70 | 378.99 | -0.3031 | 0.0141 |
| 1 | 10599773 | GPR112 | 143.12 | 141.25 | 134.70 | 143.43 | 117.61 | 127.96 | -0.2647 | 0.0340 |
| 1 | 10600284 | SRPK3 | 344.81 | 344.77 | 340.46 | 303.83 | 244.13 | 308.93 | -0.5100 | 0.0207 |
| 1 | 10601161 | GJB1 | 646.79 | 333.37 | 472.14 | 478.93 | 239.96 | 262.78 | -0.4620 | 0.0982 |
| 1 | 10601303 | CHIC1 | 400.46 | 302.80 | 319.32 | 297.89 | 207.51 | 227.50 | -0.5449 | 0.0812 |
| 1 | 10601583 | MTHFD2L | 131.58 | 134.15 | 88.08 | 85.82 | 83.16 | 82.25 | -0.6575 | 0.0812 |
| 1 | 10601846 | Arxes1/Arxes2 | 1055.90 | 453.22 | 527.55 | 1177.37 | 329.66 | 286.84 | -0.4360 | 0.0535 |
| 1 | 10603151 | GPM6B | 5796.87 | 3994.12 | 4703.03 | 4813.12 | 3000.27 | 4229.04 | -0.4108 | 0.0651 |
| 1 | 10605797 | ARHGEF9 | 915.42 | 650.19 | 688.29 | 717.15 | 312.59 | 441.97 | -1.0653 | 0.0007 |
| 1 | 10606880 | GLRA4 | 144.20 | 109.80 | 112.04 | 142.12 | 89.03 | 115.47 | -0.3040 | 0.0840 |
| 1 | 10607366 | SHROOM2 | 356.79 | 331.69 | 330.31 | 309.45 | 258.93 | 294.85 | -0.3584 | 0.0144 |
| 1 | 10608684 |  | 4080.11 | 3786.23 | 3925.88 | 3862.80 | 3271.10 | 3454.84 | -0.2097 | 0.0763 |
| 2 | 10338579 |  | 31.98 | 56.59 | 41.36 | 31.91 | 30.90 | 31.68 | -0.8325 | 0.0760 |
| 2 | 10338599 |  | 56.45 | 104.69 | 69.30 | 59.43 | 51.03 | 76.15 | -1.0303 | 0.0314 |
| 2 | 10338744 |  | 18.85 | 29.15 | 21.71 | 18.46 | 19.63 | 22.02 | -0.5659 | 0.0997 |
| 2 | 10339236 |  | 967.79 | 1357.96 | 1262.44 | 1068.63 | 989.96 | 1245.38 | -0.4553 | 0.0812 |
| 2 | 10339622 |  | 88.32 | 122.44 | 75.40 | 56.01 | 57.69 | 80.34 | -1.1205 | 0.0251 |
| 2 | 10340308 |  | 508.65 | 941.24 | 770.46 | 517.16 | 648.89 | 744.65 | -0.5461 | 0.0181 |
| 2 | 10340361 |  | 968.26 | 1181.23 | 974.03 | 937.83 | 943.35 | 1041.86 | -0.3276 | 0.0882 |
| 2 | 10340374 |  | 179.83 | 205.76 | 233.05 | 165.41 | 156.68 | 173.02 | -0.3949 | 0.0812 |
| 2 | 10340395 |  | 139.18 | 236.98 | 151.70 | 156.03 | 150.19 | 136.33 | -0.7551 | 0.0792 |
| 2 | 10340601 |  | 118.81 | 201.78 | 130.98 | 143.71 | 108.61 | 127.48 | -0.8569 | 0.0812 |
| 2 | 10340723 |  | 54.63 | 83.75 | 47.63 | 48.41 | 34.75 | 36.49 | -1.2278 | 0.0880 |
| 2 | 10340819 |  | 56.87 | 90.80 | 76.37 | 58.34 | 48.20 | 63.83 | -0.9609 | 0.0821 |
| 2 | 10340940 |  | 81.08 | 134.94 | 76.53 | 72.32 | 46.95 | 60.58 | -1.4526 | 0.0075 |
| 2 | 10340996 |  | 378.40 | 605.39 | 535.18 | 381.26 | 371.09 | 527.09 | -0.7379 | 0.0234 |
| 2 | 10341028 |  | 384.82 | 581.40 | 432.96 | 378.38 | 392.99 | 465.18 | -0.5619 | 0.0175 |
| 2 | 10341034 |  | 269.47 | 567.67 | 465.33 | 328.59 | 343.77 | 441.31 | -0.7386 | 0.0359 |
| 2 | 10341157 |  | 117.36 | 240.50 | 207.25 | 140.86 | 130.38 | 178.65 | -0.8660 | 0.0340 |
| 2 | 10342916 |  | 40.95 | 62.21 | 52.13 | 45.95 | 32.21 | 47.38 | -0.9851 | 0.0195 |
| 2 | 10342933 |  | 51.55 | 60.87 | 48.64 | 48.14 | 42.49 | 53.02 | -0.5144 | 0.0306 |
| 2 | 10343108 |  | 54.75 | 157.38 | 89.64 | 99.90 | 66.75 | 64.97 | -1.2780 | 0.0131 |
| 2 | 10343133 |  | 16.02 | 22.58 | 19.06 | 17.04 | 15.70 | 19.33 | -0.4914 | 0.0883 |
| 2 | 10343164 |  | 217.06 | 329.80 | 152.82 | 128.82 | 85.33 | 105.33 | -1.8163 | 0.0090 |
| 2 | 10343237 |  | 239.19 | 579.43 | 414.26 | 268.81 | 310.61 | 322.33 | -0.8831 | 0.0108 |

| Cluster | **Affymetrix transcript #** | Gene Symbol | Average levels of expression (arbitrary units) | | | | | | **Fold change**  **Tx_10d_ injured /Vh_10d_ injured** | |
| --- | --- | --- | --- | --- | --- | --- | --- | --- | --- | --- |
|  |  |  | **Vh_10d_ uninjured** | **Vh_10d_ injured** | **Vh_28d_ injured** | **Tx_10d_ uninjured** | **Tx_10d_ injured** | **Tx_28d_ injured** | **Log (2) Fold change** | **Adj. p-value** |
| 2 | 10343531 |  | 515.33 | 656.51 | 608.56 | 500.20 | 507.58 | 576.22 | -0.3793 | 0.0573 |
| 2 | 10344022 |  | 38.61 | 39.81 | 33.03 | 25.55 | 20.00 | 37.04 | -0.9387 | 0.0684 |
| 2 | 10345350 |  | 954.34 | 1077.23 | 1179.48 | 959.15 | 807.66 | 1092.09 | -0.4233 | 0.0514 |
| 2 | 10345715 | MAP4K4 | 2344.24 | 3096.73 | 2600.50 | 2123.34 | 2419.60 | 2361.88 | -0.3598 | 0.0849 |
| 2 | 10346882 | ADAM23 | 728.66 | 1158.78 | 883.76 | 440.31 | 530.02 | 936.06 | -1.1260 | 0.0040 |
| 2 | 10349249 | CLASP1 | 635.45 | 816.37 | 596.80 | 536.67 | 660.65 | 642.77 | -0.3030 | 0.0564 |
| 2 | 10351037 | GAS5 (includes EG:14455) | 3177.23 | 3808.74 | 3036.03 | 2758.97 | 2808.06 | 2891.70 | -0.4414 | 0.0084 |
| 2 | 10351781 | KCNJ10 | 210.88 | 692.49 | 400.84 | 181.81 | 266.31 | 362.80 | -1.3792 | 0.0000 |
| 2 | 10352306 | PYCR2 | 359.11 | 388.77 | 357.30 | 344.17 | 327.49 | 359.24 | -0.2484 | 0.0505 |
| 2 | 10352954 | HMGB3 | 529.12 | 785.85 | 630.07 | 555.82 | 567.31 | 536.11 | -0.4681 | 0.0432 |
| 2 | 10355343 | ABCA12 | 72.64 | 86.72 | 79.86 | 78.93 | 72.17 | 76.92 | -0.2689 | 0.0946 |
| 2 | 10357345 | NCKAP5 | 153.42 | 194.89 | 161.34 | 162.95 | 156.53 | 165.15 | -0.3226 | 0.0397 |
| 2 | 10357363 | NCKAP5 | 57.73 | 78.96 | 63.57 | 55.01 | 55.37 | 63.06 | -0.5114 | 0.0020 |
| 2 | 10366293 | CSRP2 | 737.48 | 3953.33 | 1347.82 | 960.78 | 1161.19 | 1303.41 | -1.7721 | 0.0000 |
| 2 | 10366645 | 1700006J14Rik | 182.78 | 315.80 | 193.45 | 209.09 | 235.60 | 195.97 | -0.4157 | 0.0443 |
| 2 | 10366983 | TMEM194A | 252.01 | 396.78 | 271.18 | 218.23 | 236.59 | 277.69 | -0.7359 | 0.0005 |
| 2 | 10367697 | PPP1R14C | 188.12 | 264.48 | 257.80 | 167.60 | 197.63 | 260.68 | -0.4150 | 0.0353 |
| 2 | 10368806 | SMPD2 | 212.27 | 270.05 | 243.94 | 237.08 | 211.77 | 243.14 | -0.3566 | 0.0719 |
| 2 | 10368999 | GRIK2 | 828.63 | 1299.10 | 1137.78 | 574.78 | 759.46 | 1308.57 | -0.7773 | 0.0266 |
| 2 | 10371466 | SYN3 | 151.84 | 171.14 | 158.50 | 147.24 | 138.71 | 160.08 | -0.3057 | 0.0505 |
| 2 | 10371784 | NR1H4 | 95.97 | 267.80 | 238.92 | 98.18 | 112.76 | 141.73 | -1.2470 | 0.0003 |
| 2 | 10371796 | SLC17A8 | 105.69 | 415.13 | 165.82 | 122.82 | 167.57 | 110.96 | -1.2746 | 0.0000 |
| 2 | 10372342 | NAV3 | 438.31 | 540.04 | 401.09 | 386.85 | 334.91 | 354.12 | -0.6838 | 0.0024 |
| 2 | 10372796 | HMGA2 | 222.60 | 467.32 | 234.73 | 276.02 | 296.63 | 238.94 | -0.6500 | 0.0040 |
| 2 | 10372891 | SRGAP1 | 504.12 | 886.89 | 564.52 | 466.50 | 551.93 | 568.03 | -0.6741 | 0.0043 |
| 2 | 10373313 | NAB2 (includes EG:100005680) | 567.05 | 723.27 | 686.23 | 545.08 | 557.28 | 604.95 | -0.3770 | 0.0300 |
| 2 | 10373467 | ERBB3 | 1130.74 | 1393.20 | 1352.38 | 926.64 | 745.13 | 1258.13 | -0.9092 | 0.0005 |
| 2 | 10373810 | DUSP18 | 618.41 | 735.64 | 504.49 | 517.18 | 407.77 | 417.41 | -0.8532 | 0.0066 |
| 2 | 10375263 | Gm9972 | 60.17 | 99.11 | 70.66 | 80.13 | 53.50 | 55.84 | -0.8483 | 0.0569 |
| 2 | 10375265 | ATP10B | 252.50 | 323.07 | 242.31 | 252.42 | 199.68 | 198.66 | -0.6824 | 0.0008 |
| 2 | 10380210 | SRSF1 | 1902.55 | 2013.33 | 1867.99 | 1745.35 | 1699.02 | 1803.76 | -0.2446 | 0.0749 |
| 2 | 10380896 | ERBB2 | 423.94 | 560.06 | 593.75 | 407.33 | 420.06 | 566.23 | -0.4259 | 0.0812 |
| 2 | 10383953 | EMID1 | 399.02 | 460.54 | 464.16 | 395.86 | 361.40 | 426.40 | -0.3542 | 0.0070 |
| 2 | 10395163 | LAMB1 | 2609.67 | 3002.60 | 2938.78 | 2436.57 | 1964.45 | 2574.40 | -0.6203 | 0.0051 |
| 2 | 10395374 | Gm5434 | 66.71 | 115.09 | 87.03 | 52.89 | 64.82 | 77.33 | -0.8240 | 0.0331 |
| 2 | 10396956 | PCNX | 777.58 | 922.80 | 779.63 | 679.49 | 712.39 | 684.27 | -0.3641 | 0.0630 |

| Cluster | **Affymetrix transcript #** | Gene Symbol | Average levels of expression (arbitrary units) | | | | | | **Fold change**  **Tx_10d_ injured /Vh_10d_ injured** | |
| --- | --- | --- | --- | --- | --- | --- | --- | --- | --- | --- |
|  |  |  | **Vh_10d_ uninjured** | **Vh_10d_ injured** | **Vh_28d_ injured** | **Tx_10d_ uninjured** | **Tx_10d_ injured** | **Tx_28d_ injured** | **Log (2) Fold change** | **Adj. p-value** |
| 2 | 10400740 |  | 289.78 | 366.28 | 331.76 | 284.47 | 286.50 | 338.73 | -0.3462 | 0.0614 |
| 2 | 10401063 | ZBTB25 | 289.16 | 383.07 | 327.39 | 295.99 | 304.47 | 287.00 | -0.3300 | 0.0851 |
| 2 | 10401595 | RPS6KL1 | 196.47 | 219.52 | 218.85 | 210.39 | 191.70 | 207.83 | -0.1954 | 0.0977 |
| 2 | 10401987 | KCNK10 | 139.21 | 211.39 | 170.03 | 135.43 | 167.93 | 198.79 | -0.3332 | 0.0862 |
| 2 | 10402708 | CKB | 955.85 | 893.12 | 1019.94 | 711.71 | 639.19 | 892.72 | -0.4922 | 0.0592 |
| 2 | 10406031 | LPCAT1 | 595.21 | 918.12 | 699.35 | 565.50 | 628.95 | 709.84 | -0.5396 | 0.0007 |
| 2 | 10406407 | ARRDC3 | 1699.63 | 1972.75 | 1504.45 | 1565.96 | 1316.49 | 1544.01 | -0.5721 | 0.0365 |
| 2 | 10406598 | SERINC5 | 931.47 | 2283.22 | 1991.81 | 833.42 | 1568.44 | 1751.29 | -0.5298 | 0.0548 |
| 2 | 10407097 | Pde4d | 260.91 | 457.56 | 341.83 | 285.61 | 334.38 | 383.64 | -0.4538 | 0.0122 |
| 2 | 10407122 |  | 207.71 | 703.69 | 352.54 | 184.46 | 312.22 | 383.47 | -1.1788 | 0.0192 |
| 2 | 10407563 |  | 458.27 | 548.60 | 483.93 | 372.81 | 429.85 | 518.87 | -0.3555 | 0.0344 |
| 2 | 10408616 | SLC22A23 | 349.58 | 859.09 | 838.34 | 333.47 | 569.68 | 699.23 | -0.5794 | 0.0039 |
| 2 | 10409616 | SPOCK1 | 243.49 | 265.83 | 239.82 | 229.81 | 207.63 | 197.20 | -0.3556 | 0.0187 |
| 2 | 10412921 | NID2 | 602.52 | 857.92 | 958.25 | 530.25 | 646.17 | 843.60 | -0.4099 | 0.0164 |
| 2 | 10417065 | RAP2A | 2595.15 | 3344.47 | 2741.21 | 2587.52 | 2682.06 | 2555.06 | -0.3141 | 0.0509 |
| 2 | 10419170 | TXNDC16 | 1301.86 | 1510.38 | 1389.47 | 1175.48 | 1056.63 | 1147.38 | -0.5118 | 0.0434 |
| 2 | 10419966 | ZFHX2 | 410.32 | 618.59 | 578.95 | 458.69 | 430.54 | 491.70 | -0.5271 | 0.0052 |
| 2 | 10422164 | EDNRB | 471.80 | 2375.70 | 1445.87 | 328.73 | 962.26 | 1257.18 | -1.2951 | 0.0004 |
| 2 | 10425116 | CDC42EP1 | 171.31 | 183.94 | 172.13 | 142.34 | 136.08 | 138.98 | -0.4290 | 0.0078 |
| 2 | 10425265 |  | 353.02 | 587.37 | 769.40 | 320.54 | 326.23 | 472.88 | -0.8720 | 0.0133 |
| 2 | 10425267 | PICK1 | 418.27 | 640.12 | 444.11 | 410.72 | 359.05 | 387.67 | -0.8344 | 0.0019 |
| 2 | 10425421 | FAM83F | 253.09 | 308.75 | 296.65 | 217.10 | 198.81 | 256.23 | -0.6261 | 0.0282 |
| 2 | 10426081 | FAM19A5 | 339.25 | 387.41 | 338.57 | 272.31 | 281.09 | 322.05 | -0.4490 | 0.0134 |
| 2 | 10426798 | SMARCD1 | 365.38 | 418.57 | 394.83 | 323.99 | 297.67 | 344.12 | -0.5056 | 0.0657 |
| 2 | 10426835 | DIP2B | 775.57 | 808.26 | 749.41 | 638.45 | 571.26 | 704.98 | -0.4938 | 0.0232 |
| 2 | 10427862 | CDH6 | 223.94 | 608.70 | 389.05 | 204.39 | 300.22 | 256.74 | -1.0166 | 0.0011 |
| 2 | 10431749 | ADAMTS20 | 1214.93 | 1653.63 | 978.00 | 1078.25 | 921.68 | 1490.92 | -0.8698 | 0.0020 |
| 2 | 10432378 | LMBR1L | 302.88 | 356.04 | 343.09 | 360.80 | 301.03 | 329.13 | -0.2433 | 0.0883 |
| 2 | 10434934 | BDH1 (includes EG:100037356) | 142.03 | 167.70 | 141.56 | 127.76 | 121.98 | 132.34 | -0.4439 | 0.0282 |
| 2 | 10435733 | IGSF11 | 750.77 | 902.84 | 732.18 | 599.90 | 520.53 | 625.04 | -0.7939 | 0.0003 |
| 2 | 10438603 | IGF2BP2 | 418.02 | 882.52 | 522.16 | 345.62 | 489.91 | 315.19 | -0.8480 | 0.0009 |
| 2 | 10438815 | MB21D2 | 237.28 | 284.92 | 243.04 | 235.75 | 230.50 | 259.49 | -0.3043 | 0.0831 |
| 2 | 10439710 | PHLDB2 | 608.02 | 1539.68 | 874.41 | 686.90 | 746.14 | 759.96 | -1.0430 | 0.0001 |
| 2 | 10439848 | Gm4827 | 80.08 | 93.96 | 88.17 | 78.41 | 74.61 | 75.74 | -0.3268 | 0.0440 |
| 2 | 10445879 | KCNH8 | 404.81 | 501.08 | 687.12 | 258.93 | 303.09 | 548.64 | -0.7281 | 0.0165 |
| 2 | 10447602 | EZR | 614.85 | 1743.00 | 1032.62 | 677.10 | 1003.16 | 853.96 | -0.7836 | 0.0002 |

| Cluster | **Affymetrix transcript #** | Gene Symbol | Average levels of expression (arbitrary units) | | | | | | **Fold change**  **Tx_10d_ injured /Vh_10d_ injured** | |
| --- | --- | --- | --- | --- | --- | --- | --- | --- | --- | --- |
|  |  |  | **Vh_10d_ uninjured** | **Vh_10d_ injured** | **Vh_28d_ injured** | **Tx_10d_ uninjured** | **Tx_10d_ injured** | **Tx_28d_ injured** | **Log (2) Fold change** | **Adj. p-value** |
| 2 | 10451061 | RUNX2 | 334.04 | 672.65 | 620.66 | 329.09 | 542.83 | 464.60 | -0.3016 | 0.0710 |
| 2 | 10454235 | ASXL3 | 758.81 | 1195.09 | 852.09 | 634.18 | 651.89 | 875.58 | -0.8823 | 0.0004 |
| 2 | 10454369 | Fhod3 (includes EG:100360334) | 232.78 | 324.55 | 331.30 | 226.05 | 215.50 | 262.56 | -0.5869 | 0.0117 |
| 2 | 10455826 | MEGF10 | 178.90 | 325.78 | 207.70 | 137.72 | 224.89 | 194.01 | -0.5300 | 0.0030 |
| 2 | 10455901 | SLC27A6 | 107.45 | 273.93 | 188.49 | 127.44 | 156.58 | 201.98 | -0.8066 | 0.0022 |
| 2 | 10457040 | ZNF516 | 482.73 | 764.41 | 580.14 | 460.83 | 565.44 | 601.56 | -0.4356 | 0.0001 |
| 2 | 10457243 | Gm10125 | 142.74 | 200.07 | 178.33 | 132.71 | 145.98 | 169.14 | -0.4318 | 0.0438 |
| 2 | 10458028 | GYPC | 434.66 | 521.60 | 570.78 | 454.16 | 373.56 | 508.83 | -0.4897 | 0.0062 |
| 2 | 10458046 | NREP | 2454.34 | 5741.96 | 4144.93 | 2154.46 | 3781.48 | 4095.81 | -0.6086 | 0.0181 |
| 2 | 10458940 | ZNF608 | 754.89 | 891.53 | 784.56 | 709.53 | 664.58 | 726.04 | -0.4243 | 0.0051 |
| 2 | 10462039 | TRPM3 | 490.48 | 1006.70 | 585.67 | 346.90 | 384.62 | 438.10 | -1.3766 | 0.0000 |
| 2 | 10462084 | mir-204 | 64.61 | 80.99 | 77.52 | 65.13 | 58.14 | 71.79 | -0.4770 | 0.0336 |
| 2 | 10462086 |  | 121.43 | 194.48 | 148.40 | 95.61 | 64.71 | 102.12 | -1.5893 | 0.0000 |
| 2 | 10462521 | PTEN | 2052.43 | 2662.71 | 2752.34 | 1992.81 | 2269.83 | 2988.22 | -0.2332 | 0.0997 |
| 2 | 10468200 | CUEDC2 | 352.11 | 665.66 | 479.54 | 343.26 | 317.30 | 414.96 | -1.0697 | 0.0000 |
| 2 | 10468668 | AFAP1L2 | 909.49 | 1000.39 | 1016.27 | 670.87 | 641.64 | 819.72 | -0.6445 | 0.0041 |
| 2 | 10468762 | KIAA1598 | 1754.24 | 2843.31 | 2215.01 | 1592.15 | 1851.92 | 1952.40 | -0.6156 | 0.0043 |
| 2 | 10469151 | ITIH5 | 1220.59 | 1599.47 | 1220.08 | 1039.00 | 947.60 | 1058.37 | -0.7540 | 0.0026 |
| 2 | 10469433 |  | 88.84 | 106.63 | 90.01 | 92.56 | 81.00 | 88.76 | -0.4031 | 0.0509 |
| 2 | 10469514 | COMMD3-BMI1 | 829.14 | 884.39 | 971.70 | 770.75 | 714.33 | 769.27 | -0.3062 | 0.0843 |
| 2 | 10469936 | NRARP | 124.01 | 169.01 | 121.44 | 139.38 | 127.06 | 127.24 | -0.4052 | 0.0943 |
| 2 | 10471882 | OLFML2A | 134.96 | 256.46 | 217.17 | 144.47 | 182.31 | 235.24 | -0.4861 | 0.0634 |
| 2 | 10473608 | OR4S2 | 63.23 | 81.08 | 61.26 | 60.77 | 62.03 | 68.63 | -0.3742 | 0.0739 |
| 2 | 10479297 | LSM14B | 425.49 | 492.46 | 464.22 | 398.96 | 402.08 | 406.54 | -0.2958 | 0.0880 |
| 2 | 10480939 | MGC50722 | 140.48 | 151.25 | 131.46 | 144.45 | 123.06 | 143.31 | -0.2953 | 0.0652 |
| 2 | 10481155 | REXO4 | 494.24 | 543.83 | 499.02 | 475.85 | 454.38 | 471.35 | -0.2612 | 0.0997 |
| 2 | 10482448 | ZEB2 | 3082.97 | 3854.49 | 4069.52 | 2768.16 | 3152.47 | 3775.42 | -0.2882 | 0.0840 |
| 2 | 10483150 | FIGN | 499.76 | 672.67 | 621.37 | 338.33 | 461.04 | 632.35 | -0.5453 | 0.0288 |
| 2 | 10490950 | BHLHE22 | 252.09 | 313.21 | 288.90 | 256.52 | 231.22 | 265.98 | -0.4414 | 0.0051 |
| 2 | 10490986 | 4632415L05Rik | 254.57 | 261.46 | 264.58 | 233.71 | 212.82 | 249.32 | -0.3073 | 0.0997 |
| 2 | 10491212 | Egfem1 | 64.25 | 79.40 | 82.42 | 68.06 | 64.76 | 74.79 | -0.2943 | 0.0507 |
| 2 | 10491477 | SOX2 | 505.08 | 911.80 | 854.80 | 377.86 | 597.85 | 638.83 | -0.6130 | 0.0489 |
| 2 | 10496519 | UNC5C | 167.76 | 252.34 | 191.84 | 179.17 | 179.33 | 175.41 | -0.4806 | 0.0275 |
| 2 | 10497051 | NEGR1 | 388.64 | 367.88 | 431.19 | 257.16 | 236.35 | 415.42 | -0.6453 | 0.0305 |

| Cluster | **Affymetrix transcript #** | Gene Symbol | Average levels of expression (arbitrary units) | | | | | | **Fold change**  **Tx_10d_ injured /Vh_10d_ injured** | |
| --- | --- | --- | --- | --- | --- | --- | --- | --- | --- | --- |
|  |  |  | **Vh_10d_ uninjured** | **Vh_10d_ injured** | **Vh_28d_ injured** | **Tx_10d_ uninjured** | **Tx_10d_ injured** | **Tx_28d_ injured** | **Log (2) Fold change** | **Adj. p-value** |
| 2 | 10498076 | MAML3 | 263.87 | 368.77 | 311.95 | 251.80 | 260.14 | 282.29 | -0.5047 | 0.0040 |
| 2 | 10499138 | DCLK2 | 324.86 | 364.71 | 380.95 | 262.64 | 273.64 | 297.00 | -0.4133 | 0.0268 |
| 2 | 10501319 | CELSR2 | 274.04 | 299.92 | 274.21 | 263.61 | 233.67 | 253.41 | -0.3599 | 0.0398 |
| 2 | 10503448 | MMP16 | 246.82 | 333.26 | 315.04 | 162.17 | 214.76 | 265.41 | -0.6434 | 0.0385 |
| 2 | 10503695 | BACH2 | 135.82 | 166.82 | 157.39 | 143.31 | 134.24 | 159.66 | -0.3112 | 0.0946 |
| 2 | 10505064 | TMEM38B | 339.77 | 444.76 | 355.63 | 353.37 | 291.88 | 320.71 | -0.6027 | 0.0020 |
| 2 | 10505614 | TYRP1 | 98.89 | 709.50 | 230.15 | 117.92 | 165.03 | 203.59 | -2.0087 | 0.0000 |
| 2 | 10505623 | LURAP1L | 158.11 | 669.05 | 222.26 | 213.03 | 376.79 | 248.83 | -0.8074 | 0.0016 |
| 2 | 10508217 | SFPQ | 3744.89 | 4576.97 | 3953.26 | 3527.69 | 3819.68 | 3692.03 | -0.2671 | 0.0349 |
| 2 | 10509410 | RAP1GAP | 184.73 | 206.83 | 208.64 | 167.72 | 131.67 | 160.53 | -0.6538 | 0.0062 |
| 2 | 10510668 | TNFRSF25 | 217.33 | 247.88 | 237.69 | 223.71 | 187.48 | 213.27 | -0.4002 | 0.0123 |
| 2 | 10511810 | POU3F2 | 268.07 | 568.42 | 384.01 | 189.68 | 237.39 | 278.31 | -1.2603 | 0.0046 |
| 2 | 10512709 | SLC25A51 | 1453.39 | 2173.29 | 1754.96 | 1329.38 | 1747.42 | 1637.65 | -0.3150 | 0.0465 |
| 2 | 10514088 | FREM1 | 379.89 | 442.65 | 377.21 | 331.65 | 235.94 | 352.40 | -0.8965 | 0.0001 |
| 2 | 10514510 | CYP2J2 | 1508.69 | 1781.48 | 2088.11 | 1185.33 | 1091.62 | 1678.05 | -0.7059 | 0.0022 |
| 2 | 10515894 | CCDC30 | 61.75 | 77.65 | 69.10 | 63.86 | 54.51 | 67.17 | -0.5071 | 0.0141 |
| 2 | 10516227 | 3100002H09Rik | 200.23 | 270.75 | 248.37 | 234.42 | 189.59 | 234.90 | -0.5146 | 0.0005 |
| 2 | 10519747 | SEMA3E | 371.98 | 354.85 | 565.01 | 300.03 | 272.70 | 444.93 | -0.3758 | 0.0557 |
| 2 | 10521337 | RGS12 | 180.42 | 203.33 | 175.93 | 166.63 | 171.53 | 179.00 | -0.2460 | 0.0282 |
| 2 | 10525542 | BCL7A | 695.21 | 853.54 | 811.83 | 713.82 | 672.84 | 760.26 | -0.3433 | 0.0816 |
| 2 | 10526726 | ZKSCAN1 | 583.06 | 638.23 | 618.69 | 533.26 | 535.77 | 528.02 | -0.2521 | 0.0510 |
| 2 | 10530870 | EPHA5 | 305.03 | 1220.04 | 897.31 | 216.54 | 634.44 | 400.94 | -0.9117 | 0.0331 |
| 2 | 10534596 | CUX1 | 1029.68 | 1150.86 | 987.11 | 898.48 | 780.35 | 877.38 | -0.5616 | 0.0000 |
| 2 | 10534960 | GJC3 | 3259.92 | 4754.48 | 4766.34 | 2424.25 | 1849.23 | 4065.40 | -1.3595 | 0.0000 |
| 2 | 10537026 | CPA4 | 69.67 | 119.59 | 97.08 | 79.56 | 95.84 | 92.91 | -0.3201 | 0.0509 |
| 2 | 10538269 | DFNA5 | 216.21 | 409.69 | 278.03 | 214.12 | 150.36 | 182.08 | -1.4643 | 0.0001 |
| 2 | 10539169 | LRRTM1 | 62.84 | 74.89 | 62.53 | 59.44 | 59.20 | 62.40 | -0.3384 | 0.0813 |
| 2 | 10539211 | LRRTM4 | 447.15 | 565.04 | 511.17 | 342.76 | 275.61 | 482.13 | -1.0295 | 0.0018 |
| 2 | 10541803 | CHD4 | 2124.73 | 2863.80 | 2358.77 | 1995.10 | 2090.44 | 2233.61 | -0.4585 | 0.0035 |
| 2 | 10542522 | PLEKHA5 | 638.64 | 686.52 | 738.30 | 567.04 | 531.16 | 648.19 | -0.3692 | 0.0259 |
| 2 | 10543591 | OPN1SW | 107.07 | 142.76 | 124.17 | 120.38 | 114.03 | 121.30 | -0.3282 | 0.0870 |
| 2 | 10545298 | RMND5A | 662.41 | 714.61 | 631.03 | 596.65 | 556.13 | 616.57 | -0.3661 | 0.0424 |
| 2 | 10548051 | KCNA6 | 443.85 | 587.62 | 714.37 | 398.72 | 381.92 | 687.83 | -0.6319 | 0.0127 |
| 2 | 10548735 | DUSP16 | 605.75 | 967.28 | 629.52 | 556.13 | 547.60 | 595.24 | -0.8213 | 0.0000 |
| 2 | 10549200 | SOX5 | 174.49 | 293.67 | 258.40 | 214.70 | 209.75 | 232.26 | -0.4902 | 0.0049 |
| 2 | 10549594 | TTYH1 | 1028.56 | 1422.09 | 1283.95 | 852.39 | 881.54 | 1174.50 | -0.6941 | 0.0021 |
|  |  |  |  |  |  |  |  |  |  |  |

| Cluster | **Affymetrix transcript #** | Gene Symbol | Average levels of expression (arbitrary units) | | | | | | **Fold change**  **Tx_10d_ injured /Vh_10d_ injured** | |
| --- | --- | --- | --- | --- | --- | --- | --- | --- | --- | --- |
|  |  |  | **Vh_10d_ uninjured** | **Vh_10d_ injured** | **Vh_28d_ injured** | **Tx_10d_ uninjured** | **Tx_10d_ injured** | **Tx_28d_ injured** | **Log (2) Fold change** | **Adj. p-value** |
| 2 | 10551421 | Zfp60 (includes others) | 57.49 | 93.67 | 61.90 | 52.65 | 53.29 | 52.46 | -0.7801 | 0.0314 |
| 2 | 10553071 | NTN5 | 192.71 | 256.14 | 307.28 | 191.51 | 191.77 | 219.95 | -0.4052 | 0.0237 |
| 2 | 10553280 | GTF2H1 | 616.98 | 956.52 | 698.79 | 636.59 | 724.35 | 648.76 | -0.4028 | 0.0164 |
| 2 | 10553354 | NAV2 | 224.11 | 592.52 | 373.59 | 226.52 | 455.33 | 351.17 | -0.3700 | 0.0503 |
| 2 | 10553537 | LUZP2 | 504.22 | 548.91 | 596.62 | 289.59 | 308.15 | 418.92 | -0.8294 | 0.0001 |
| 2 | 10554045 | ADAMTS17 | 229.50 | 401.31 | 350.41 | 223.64 | 223.50 | 284.09 | -0.8400 | 0.0001 |
| 2 | 10554057 | ADAMTS17 | 677.29 | 930.93 | 891.76 | 799.41 | 731.63 | 833.90 | -0.3451 | 0.0340 |
| 2 | 10554063 | ADAMTS17 | 158.49 | 309.63 | 274.21 | 157.42 | 182.23 | 222.27 | -0.7694 | 0.0141 |
| 2 | 10554325 | C15orf42 | 75.12 | 128.28 | 80.97 | 90.03 | 101.60 | 89.75 | -0.3353 | 0.0790 |
| 2 | 10554588 | SH3GL3 | 146.96 | 320.58 | 172.61 | 130.25 | 138.43 | 177.30 | -1.1903 | 0.0001 |
| 2 | 10554800 | RAB38 | 317.08 | 449.86 | 305.38 | 252.69 | 325.48 | 389.76 | -0.4628 | 0.0136 |
| 2 | 10556442 | TEAD1 | 1364.13 | 2016.95 | 1494.34 | 1270.56 | 1338.14 | 1387.06 | -0.5900 | 0.0041 |
| 2 | 10556456 | TEAD1 | 3637.86 | 4737.77 | 3791.79 | 3502.47 | 3280.93 | 3839.38 | -0.5312 | 0.0090 |
| 2 | 10556553 | INSC | 423.68 | 572.40 | 549.99 | 358.10 | 414.42 | 508.83 | -0.4560 | 0.0173 |
| 2 | 10558134 | PLEKHA1 | 2049.20 | 3294.10 | 2840.64 | 1803.80 | 2129.18 | 2504.85 | -0.6280 | 0.0066 |
| 2 | 10558345 | DOCK1 | 958.99 | 1093.73 | 1029.16 | 919.41 | 875.93 | 960.42 | -0.3210 | 0.0826 |
| 2 | 10559146 | BRSK2 (includes EG:100334759) | 254.84 | 276.40 | 232.69 | 224.98 | 199.07 | 213.07 | -0.4732 | 0.0307 |
| 2 | 10559500 | D030047H15Rik | 119.16 | 154.98 | 146.01 | 123.94 | 122.00 | 137.59 | -0.3488 | 0.0403 |
| 2 | 10562532 | ZNF536 | 194.07 | 206.19 | 187.29 | 160.87 | 147.74 | 186.05 | -0.4753 | 0.0645 |
| 2 | 10565689 | CAPN5 | 426.62 | 580.35 | 598.57 | 407.09 | 413.13 | 511.31 | -0.4914 | 0.0347 |
| 2 | 10567316 | TMC7 | 319.26 | 895.78 | 577.90 | 279.55 | 504.18 | 545.47 | -0.8034 | 0.0061 |
| 2 | 10568705 | FAM196A | 913.35 | 1202.43 | 1067.49 | 874.13 | 1023.00 | 1010.59 | -0.2316 | 0.0954 |
| 2 | 10569278 | DUSP8 | 216.99 | 275.51 | 215.26 | 185.98 | 202.63 | 228.46 | -0.4378 | 0.0252 |
| 2 | 10569569 | CTTN | 916.61 | 1693.24 | 1277.44 | 874.94 | 1269.10 | 1198.29 | -0.4158 | 0.0599 |
| 2 | 10569583 | PPFIA1 | 917.46 | 968.31 | 888.54 | 899.55 | 827.09 | 843.29 | -0.2321 | 0.0727 |
| 2 | 10571246 | 5930422O12Rik | 138.87 | 184.79 | 174.21 | 140.70 | 100.46 | 133.51 | -0.8830 | 0.0914 |
| 2 | 10571312 | DUSP4 | 149.88 | 257.20 | 169.61 | 164.30 | 144.24 | 149.57 | -0.8323 | 0.0002 |
| 2 | 10573779 | NKD1 | 518.01 | 786.48 | 645.25 | 505.50 | 387.43 | 522.10 | -1.0131 | 0.0001 |
| 2 | 10578649 | ODZ3 | 786.30 | 2126.35 | 1676.08 | 610.34 | 1442.86 | 1386.23 | -0.5526 | 0.0366 |
| 2 | 10580365 | C16orf87 | 776.01 | 1056.60 | 897.69 | 742.95 | 730.56 | 900.54 | -0.5390 | 0.0131 |
| 2 | 10580382 | NETO2 | 87.31 | 204.77 | 98.84 | 52.56 | 114.44 | 105.47 | -0.8162 | 0.0156 |
| 2 | 10583215 | MTMR2 | 754.01 | 967.14 | 799.64 | 641.16 | 716.09 | 623.98 | -0.4288 | 0.0741 |
| 2 | 10584674 | MCAM | 893.07 | 1921.00 | 1467.63 | 965.41 | 1524.11 | 1702.31 | -0.3344 | 0.0342 |
| 2 | 10585048 | CADM1 | 1811.79 | 2748.44 | 3274.66 | 1444.02 | 1786.74 | 2648.15 | -0.6180 | 0.0006 |
| 2 | 10585099 | USP28 | 242.95 | 279.99 | 263.91 | 244.66 | 229.11 | 272.30 | -0.2869 | 0.0812 |

| Cluster | **Affymetrix transcript #** | Gene Symbol | Average levels of expression (arbitrary units) | | | | | | **Fold change**  **Tx_10d_ injured /Vh_10d_ injured** | |
| --- | --- | --- | --- | --- | --- | --- | --- | --- | --- | --- |
|  |  |  | **Vh_10d_ uninjured** | **Vh_10d_ injured** | **Vh_28d_ injured** | **Tx_10d_ uninjured** | **Tx_10d_ injured** | **Tx_28d_ injured** | **Log (2) Fold change** | **Adj. p-value** |
| 2 | 10586967 | Gm7265 | 449.07 | 581.37 | 574.80 | 441.28 | 466.92 | 478.78 | -0.3195 | 0.0408 |
| 2 | 10587299 | ICK | 634.32 | 1122.69 | 761.54 | 539.71 | 605.65 | 747.74 | -0.8942 | 0.0001 |
| 2 | 10589327 | UCN2 | 174.07 | 506.34 | 279.73 | 197.46 | 344.62 | 249.54 | -0.5446 | 0.0534 |
| 2 | 10589329 | PFKFB4 | 117.91 | 239.64 | 191.01 | 125.84 | 171.39 | 173.62 | -0.4765 | 0.0097 |
| 2 | 10590933 | PIWIL4 | 149.34 | 316.72 | 201.82 | 151.77 | 161.55 | 180.98 | -0.9192 | 0.0007 |
| 2 | 10591978 | NTM | 249.03 | 409.04 | 221.14 | 172.86 | 244.25 | 274.17 | -0.7422 | 0.0822 |
| 2 | 10593159 | PAFAH1B2 | 2061.58 | 2335.53 | 1954.72 | 1758.08 | 1836.01 | 1787.57 | -0.3474 | 0.0385 |
| 2 | 10593196 | Gm10677 | 367.15 | 527.67 | 486.35 | 343.07 | 350.87 | 503.80 | -0.5820 | 0.0128 |
| 2 | 10593471 | mir-34 | 295.06 | 324.46 | 340.36 | 299.82 | 236.97 | 293.12 | -0.4482 | 0.0064 |
| 2 | 10594301 | CORO2B | 152.30 | 277.98 | 194.20 | 127.64 | 183.02 | 185.98 | -0.6018 | 0.0046 |
| 2 | 10594679 | TLN2 | 300.32 | 471.92 | 381.64 | 322.16 | 382.56 | 434.54 | -0.2984 | 0.0551 |
| 2 | 10596545 | RAD54L2 | 404.95 | 549.30 | 484.30 | 483.05 | 440.84 | 465.08 | -0.3177 | 0.0813 |
| 2 | 10597395 | 4921528I07Rik/Gm2396 | 147.88 | 192.43 | 159.48 | 168.29 | 132.77 | 156.68 | -0.5067 | 0.0759 |
| 2 | 10600017 | HMGB3 | 595.76 | 829.67 | 711.91 | 634.11 | 643.50 | 612.45 | -0.3662 | 0.0791 |
| 2 | 10600249 | PLXNB3 | 503.49 | 708.22 | 625.26 | 459.34 | 400.36 | 528.51 | -0.8229 | 0.0021 |
| 2 | 10601164 | NONO | 1646.14 | 2275.07 | 1859.30 | 1502.33 | 1855.00 | 1746.24 | -0.2932 | 0.0792 |
| 2 | 10601888 | PLP1 (includes EG:18823) | 6749.84 | 6947.54 | 7448.04 | 4635.32 | 4751.01 | 6989.43 | -0.5489 | 0.0144 |
| 2 | 10603310 | mir-188 | 114.79 | 130.31 | 117.64 | 125.24 | 106.59 | 123.70 | -0.2914 | 0.0492 |
| 2 | 10608667 |  | 1272.97 | 1679.32 | 1313.01 | 1070.98 | 1249.94 | 1295.43 | -0.4259 | 0.0663 |
| 3 | 10339641 |  | 315.81 | 881.83 | 1050.13 | 213.46 | 1435.03 | 1381.19 | 0.7302 | 0.0740 |
| 3 | 10339936 |  | 1133.79 | 1969.78 | 2164.10 | 1113.65 | 3048.68 | 2502.31 | 0.6462 | 0.0792 |
| 3 | 10341637 |  | 1556.22 | 2871.01 | 3438.14 | 1584.76 | 4165.75 | 3715.84 | 0.5409 | 0.0557 |
| 3 | 10341744 |  | 206.01 | 212.62 | 181.84 | 215.27 | 313.93 | 226.51 | 0.5653 | 0.0489 |
| 3 | 10342261 |  | 948.72 | 1620.00 | 1719.26 | 914.36 | 2446.96 | 2345.18 | 0.6036 | 0.0241 |
| 3 | 10343175 |  | 171.09 | 160.61 | 191.73 | 180.49 | 257.61 | 140.94 | 0.6299 | 0.0932 |
| 3 | 10343203 |  | 22.52 | 23.26 | 22.56 | 23.05 | 36.45 | 18.39 | 0.6043 | 0.0855 |
| 3 | 10343866 |  | 1900.04 | 3358.18 | 4060.78 | 1951.31 | 4895.43 | 4439.63 | 0.5451 | 0.0490 |
| 3 | 10344191 |  | 25.31 | 24.96 | 33.21 | 26.46 | 41.22 | 21.83 | 0.7045 | 0.0890 |
| 3 | 10344495 |  | 652.10 | 619.61 | 637.53 | 590.03 | 850.26 | 660.49 | 0.4569 | 0.0490 |
| 3 | 10344616 |  | 17.33 | 16.50 | 18.06 | 17.14 | 19.20 | 18.08 | 0.2181 | 0.0671 |
| 3 | 10345807 | IL18R1 | 95.84 | 132.29 | 205.99 | 95.78 | 186.23 | 228.01 | 0.4963 | 0.0314 |
| 3 | 10345921 | C2orf40 | 169.70 | 222.02 | 185.46 | 223.98 | 348.19 | 214.50 | 0.6406 | 0.0503 |
| 3 | 10346191 | STAT1 | 648.40 | 606.35 | 646.09 | 586.34 | 793.07 | 676.34 | 0.3899 | 0.0809 |
| 3 | 10347915 | Csprs (includes others) | 76.59 | 81.24 | 81.57 | 74.56 | 99.52 | 88.95 | 0.2869 | 0.0875 |
| 3 | 10347921 | A530040E14Rik | 265.95 | 415.01 | 425.82 | 271.43 | 655.88 | 527.50 | 0.6685 | 0.0165 |

| Cluster | **Affymetrix transcript #** | Gene Symbol | Average levels of expression (arbitrary units) | | | | | | **Fold change**  **Tx_10d_ injured /Vh_10d_ injured** | |
| --- | --- | --- | --- | --- | --- | --- | --- | --- | --- | --- |
|  |  |  | **Vh_10d_ uninjured** | **Vh_10d_ injured** | **Vh_28d_ injured** | **Tx_10d_ uninjured** | **Tx_10d_ injured** | **Tx_28d_ injured** | **Log (2) Fold change** | **Adj. p-value** |
| 3 | 10347931 | G530012D18Rik | 1305.25 | 3162.78 | 3555.63 | 2545.14 | 5715.18 | 4251.92 | 0.8367 | 0.0092 |
| 3 | 10347948 | Sp100 | 464.78 | 581.25 | 612.71 | 648.89 | 1074.14 | 824.01 | 0.9472 | 0.0232 |
| 3 | 10348244 | INPP5D | 218.58 | 463.44 | 456.35 | 228.11 | 674.35 | 561.84 | 0.5371 | 0.0482 |
| 3 | 10350349 | DENND1B | 377.65 | 456.52 | 477.67 | 322.73 | 568.10 | 474.76 | 0.3182 | 0.0774 |
| 3 | 10351259 | SLC19A2 | 166.41 | 194.23 | 194.15 | 147.91 | 261.90 | 193.37 | 0.4254 | 0.0128 |
| 3 | 10351347 | CREG1 | 365.42 | 722.59 | 904.58 | 382.05 | 1256.99 | 1152.61 | 0.7818 | 0.0112 |
| 3 | 10351509 | FCGR3A | 86.27 | 175.73 | 213.37 | 81.69 | 533.59 | 331.74 | 1.5451 | 0.0001 |
| 3 | 10351658 | CD48 | 147.85 | 642.08 | 677.17 | 218.00 | 1103.73 | 775.26 | 0.7959 | 0.0524 |
| 3 | 10351679 | CD84 | 95.12 | 691.95 | 807.67 | 149.23 | 1173.28 | 976.11 | 0.7760 | 0.0366 |
| 3 | 10351691 | SLAMF6 | 61.57 | 81.79 | 90.32 | 61.49 | 131.35 | 87.70 | 0.6482 | 0.0048 |
| 3 | 10351873 | Ifi204 (includes others) | 88.37 | 181.66 | 180.85 | 96.35 | 624.11 | 245.59 | 1.8174 | 0.0012 |
| 3 | 10351880 | Ifi204 (includes others) | 44.94 | 40.60 | 39.52 | 37.83 | 53.75 | 45.25 | 0.4141 | 0.0470 |
| 3 | 10354677 | ANKRD44 | 351.88 | 337.13 | 415.88 | 355.44 | 427.28 | 491.00 | 0.3340 | 0.0906 |
| 3 | 10356248 | A530032D15Rik (includes others) | 120.11 | 172.16 | 170.24 | 130.03 | 266.21 | 213.63 | 0.6206 | 0.0026 |
| 3 | 10356274 | Csprs (includes others) | 125.27 | 127.52 | 134.96 | 124.85 | 166.77 | 140.11 | 0.3895 | 0.0392 |
| 3 | 10356278 |  | 322.86 | 539.92 | 538.19 | 353.20 | 924.21 | 611.12 | 0.7626 | 0.0044 |
| 3 | 10357239 | TMEM37 | 208.92 | 290.15 | 345.37 | 244.34 | 436.96 | 297.01 | 0.5649 | 0.0160 |
| 3 | 10358038 | RNPEP | 159.66 | 234.27 | 228.55 | 168.00 | 332.08 | 269.04 | 0.4974 | 0.0268 |
| 3 | 10358408 | RGS1 | 56.01 | 105.58 | 86.35 | 64.43 | 142.42 | 98.02 | 0.4290 | 0.0607 |
| 3 | 10360028 | FCGR2B | 216.49 | 405.90 | 436.68 | 231.30 | 586.46 | 508.74 | 0.5313 | 0.0409 |
| 3 | 10360040 | FCGR2A | 177.03 | 481.35 | 565.41 | 247.91 | 842.89 | 763.20 | 0.8322 | 0.0303 |
| 3 | 10360070 | FCER1G | 249.98 | 1070.63 | 1153.34 | 385.48 | 1974.14 | 1456.35 | 0.8886 | 0.0048 |
| 3 | 10360158 | LY9 | 351.83 | 731.12 | 939.59 | 368.02 | 1199.70 | 1207.36 | 0.7248 | 0.0364 |
| 3 | 10360173 | SLAMF7 | 93.30 | 390.19 | 693.03 | 144.48 | 881.82 | 1033.13 | 1.1932 | 0.0009 |
| 3 | 10360367 |  | 61.00 | 72.42 | 82.23 | 83.52 | 218.19 | 129.41 | 1.6039 | 0.0000 |
| 3 | 10360370 | Ifi204 (includes others) | 36.55 | 47.05 | 45.19 | 50.39 | 69.49 | 57.31 | 0.5633 | 0.0616 |
| 3 | 10360377 | Ifi204 (includes others) | 316.96 | 781.35 | 600.50 | 592.90 | 1801.79 | 920.73 | 1.2457 | 0.0710 |
| 3 | 10361186 | SERTAD4 | 237.67 | 309.75 | 308.60 | 278.61 | 410.06 | 352.22 | 0.4145 | 0.0532 |
| 3 | 10361790 | FUCA2 | 382.52 | 595.15 | 542.98 | 405.46 | 851.76 | 692.15 | 0.5234 | 0.0824 |
| 3 | 10362091 | Raet1b | 29.10 | 31.54 | 26.96 | 25.77 | 41.35 | 31.70 | 0.3812 | 0.0792 |

| Cluster | **Affymetrix transcript #** | Gene Symbol | Average levels of expression (arbitrary units) | | | | | | **Fold change**  **Tx_10d_ injured /Vh_10d_ injured** | |
| --- | --- | --- | --- | --- | --- | --- | --- | --- | --- | --- |
|  |  |  | **Vh_10d_ uninjured** | **Vh_10d_ injured** | **Vh_28d_ injured** | **Tx_10d_ uninjured** | **Tx_10d_ injured** | **Tx_28d_ injured** | **Log (2) Fold change** | **Adj. p-value** |
| 3 | 10363070 | Gp49a/Lilrb4 | 183.54 | 976.18 | 1319.92 | 287.41 | 1748.86 | 1830.97 | 0.8232 | 0.0847 |
| 3 | 10363082 | Gp49a/Lilrb4 | 160.63 | 855.91 | 1105.18 | 285.28 | 1404.23 | 1516.79 | 0.7121 | 0.0204 |
| 3 | 10364262 | ITGB2 | 125.03 | 330.30 | 374.29 | 158.30 | 460.49 | 420.83 | 0.4806 | 0.0554 |
| 3 | 10364375 | CSTB | 938.68 | 1590.07 | 1723.43 | 1166.41 | 2854.44 | 2208.12 | 0.8135 | 0.0098 |
| 3 | 10366310 | OSBPL8 | 779.80 | 1171.22 | 1202.99 | 654.30 | 1537.95 | 1407.80 | 0.3864 | 0.0823 |
| 3 | 10366653 | WIF1 | 142.92 | 242.52 | 171.81 | 148.04 | 340.43 | 190.18 | 0.4945 | 0.0097 |
| 3 | 10366667 | GNS | 690.62 | 1173.01 | 1135.57 | 676.73 | 1695.73 | 1274.70 | 0.5328 | 0.0058 |
| 3 | 10366881 | DDIT3 | 875.40 | 1045.68 | 994.89 | 932.98 | 1340.99 | 1090.29 | 0.3620 | 0.0791 |
| 3 | 10367224 | STAT2 | 421.19 | 402.03 | 392.04 | 426.63 | 558.33 | 415.10 | 0.4783 | 0.0208 |
| 3 | 10367634 | AKAP12 | 1041.06 | 1015.11 | 936.01 | 1016.79 | 1257.00 | 1248.76 | 0.3030 | 0.0575 |
| 3 | 10368720 | SLC16A10 | 97.29 | 125.19 | 135.04 | 110.56 | 167.38 | 144.01 | 0.4134 | 0.0532 |
| 3 | 10368947 | AIM1 (includes EG:11630) | 113.18 | 125.29 | 133.99 | 119.55 | 167.84 | 177.54 | 0.4117 | 0.0604 |
| 3 | 10369379 | SLC29A3 | 427.77 | 474.65 | 533.93 | 397.81 | 594.32 | 537.56 | 0.3230 | 0.0298 |
| 3 | 10369413 | SGPL1 | 625.96 | 1070.53 | 1044.25 | 618.46 | 1360.79 | 1153.43 | 0.3504 | 0.0592 |
| 3 | 10369615 | SRGN | 1358.83 | 1269.21 | 1528.85 | 1567.08 | 2140.15 | 1711.46 | 0.7598 | 0.0027 |
| 3 | 10369932 | SUSD2 | 199.68 | 182.06 | 202.98 | 215.30 | 248.91 | 224.67 | 0.4611 | 0.0493 |
| 3 | 10372028 | PLXNC1 | 173.10 | 332.47 | 342.96 | 169.90 | 474.00 | 392.84 | 0.5248 | 0.0992 |
| 3 | 10372410 | GLIPR1 | 332.67 | 495.92 | 541.11 | 276.32 | 763.11 | 668.68 | 0.5697 | 0.0465 |
| 3 | 10372668 | MDM2 | 810.35 | 1004.97 | 842.77 | 768.64 | 1529.74 | 926.50 | 0.6129 | 0.0007 |
| 3 | 10372716 | RAP1B | 3900.96 | 4144.84 | 4186.38 | 3910.97 | 4873.70 | 4771.23 | 0.2328 | 0.0965 |
| 3 | 10372988 | SLC16A7 | 227.75 | 243.52 | 251.46 | 248.79 | 344.60 | 297.51 | 0.5024 | 0.0385 |
| 3 | 10374333 | IKZF1 | 144.38 | 256.44 | 274.62 | 180.17 | 352.64 | 310.75 | 0.4582 | 0.0141 |
| 3 | 10375145 | LCP2 | 218.29 | 485.85 | 570.48 | 252.35 | 719.11 | 716.78 | 0.5493 | 0.0875 |
| 3 | 10375443 | HAVCR2 | 91.67 | 251.93 | 232.64 | 104.62 | 415.80 | 338.59 | 0.7123 | 0.0436 |
| 3 | 10375515 | Olfr56 | 91.47 | 76.97 | 90.03 | 88.93 | 147.33 | 125.75 | 0.9373 | 0.0013 |
| 3 | 10376324 | Gm12250 | 134.17 | 122.30 | 127.33 | 135.30 | 226.87 | 148.86 | 0.8756 | 0.0138 |
| 3 | 10376868 | TRPV2 | 127.70 | 163.25 | 180.17 | 134.92 | 225.18 | 193.13 | 0.4608 | 0.0105 |
| 3 | 10379389 | ADAP2 | 199.39 | 349.58 | 369.15 | 166.86 | 485.36 | 401.08 | 0.4726 | 0.0580 |
| 3 | 10379535 | CCL8 | 98.49 | 307.05 | 135.83 | 109.41 | 765.75 | 192.79 | 1.1623 | 0.0084 |
| 3 | 10379630 | SLFN12L | 163.04 | 372.48 | 314.42 | 232.32 | 683.59 | 356.80 | 0.8811 | 0.0044 |
| 3 | 10379633 | Slfn1 | 156.78 | 269.65 | 196.58 | 210.48 | 987.44 | 299.67 | 1.7828 | 0.0001 |
| 3 | 10379636 | SLFN12 | 90.03 | 109.63 | 96.16 | 104.00 | 197.33 | 105.32 | 0.8046 | 0.0114 |
| 3 | 10381445 | TMEM106A | 148.55 | 381.70 | 366.73 | 190.83 | 654.12 | 455.44 | 0.7605 | 0.0022 |
| 3 | 10382106 | Gm885 | 105.89 | 203.95 | 226.34 | 134.26 | 339.45 | 304.57 | 0.7229 | 0.0399 |
| 3 | 10382438 | CD300A | 231.24 | 562.80 | 592.76 | 254.08 | 887.68 | 741.64 | 0.6551 | 0.0012 |
| 3 | 10384458 | PLEK | 296.87 | 1262.22 | 1349.02 | 357.96 | 1915.30 | 1753.57 | 0.6109 | 0.0207 |

| Cluster | **Affymetrix transcript #** | Gene Symbol | Average levels of expression (arbitrary units) | | | | | | **Fold change**  **Tx_10d_ injured /Vh_10d_ injured** | |
| --- | --- | --- | --- | --- | --- | --- | --- | --- | --- | --- |
|  |  |  | **Vh_10d_ uninjured** | **Vh_10d_ injured** | **Vh_28d_ injured** | **Tx_10d_ uninjured** | **Tx_10d_ injured** | **Tx_28d_ injured** | **Log (2) Fold change** | **Adj. p-value** |
| 3 | 10385271 | CCNG1 | 2310.45 | 2520.44 | 2170.92 | 2618.53 | 3707.18 | 2532.38 | 0.5586 | 0.0000 |
| 3 | 10385500 | IRGM | 169.96 | 214.78 | 189.92 | 166.44 | 528.92 | 271.17 | 1.2925 | 0.0001 |
| 3 | 10385504 | Gm5431 | 49.29 | 75.93 | 72.47 | 43.62 | 173.47 | 102.31 | 1.1714 | 0.0038 |
| 3 | 10385511 | PSME2 | 471.17 | 610.77 | 632.17 | 602.29 | 872.03 | 795.54 | 0.5152 | 0.0051 |
| 3 | 10385513 | 9930111J21Rik2 | 211.71 | 344.24 | 341.77 | 225.72 | 631.06 | 436.73 | 0.8714 | 0.0048 |
| 3 | 10385518 | Tgtp1/Tgtp2 | 365.14 | 289.52 | 290.52 | 395.19 | 556.81 | 411.09 | 0.9413 | 0.0075 |
| 3 | 10385526 | 9930111J21Rik2 | 219.72 | 338.85 | 340.98 | 221.70 | 622.95 | 449.07 | 0.8755 | 0.0070 |
| 3 | 10387536 | CD68 | 325.93 | 2865.05 | 3006.74 | 643.14 | 4750.96 | 3611.82 | 0.7275 | 0.0314 |
| 3 | 10387890 | CXCL16 | 127.86 | 326.35 | 397.15 | 127.45 | 507.85 | 436.38 | 0.6308 | 0.0266 |
| 3 | 10387983 |  | 3182.30 | 3785.41 | 3882.45 | 3726.08 | 4492.15 | 4255.71 | 0.2447 | 0.0833 |
| 3 | 10388902 | LGALS9 | 509.00 | 650.21 | 629.78 | 476.18 | 1134.98 | 837.82 | 0.8178 | 0.0066 |
| 3 | 10389134 | SLFN13 | 96.23 | 176.01 | 130.47 | 113.90 | 226.25 | 170.56 | 0.3583 | 0.0743 |
| 3 | 10389143 | SLFN13 | 88.66 | 167.29 | 141.98 | 91.58 | 360.52 | 247.44 | 1.0912 | 0.0146 |
| 3 | 10389151 | SLFN13 | 104.44 | 177.24 | 158.67 | 122.70 | 279.35 | 219.68 | 0.6559 | 0.0073 |
| 3 | 10389207 | CCL5 | 316.07 | 442.42 | 468.84 | 304.30 | 762.29 | 631.35 | 0.7646 | 0.0013 |
| 3 | 10389894 | ABCC3 | 122.33 | 253.93 | 292.70 | 133.04 | 411.77 | 324.69 | 0.6962 | 0.0088 |
| 3 | 10390640 | IKZF3 | 92.15 | 82.14 | 112.91 | 96.58 | 110.21 | 121.49 | 0.4235 | 0.0232 |
| 3 | 10391207 | DHX58 | 79.47 | 93.83 | 88.23 | 79.06 | 141.21 | 101.21 | 0.5469 | 0.0598 |
| 3 | 10392440 | SLC16A6 | 166.00 | 238.90 | 245.55 | 158.63 | 319.78 | 307.54 | 0.4158 | 0.0366 |
| 3 | 10392815 | CD300C | 170.07 | 466.34 | 524.86 | 235.88 | 820.03 | 628.13 | 0.8074 | 0.0077 |
| 3 | 10392825 | Cd300lh (includes others) | 382.14 | 773.48 | 813.57 | 528.99 | 1278.72 | 702.12 | 0.7531 | 0.0119 |
| 3 | 10392834 | Cd300lh (includes others) | 363.47 | 703.44 | 734.57 | 488.11 | 1180.26 | 629.10 | 0.7792 | 0.0131 |
| 3 | 10392845 | CD300LF | 133.40 | 333.26 | 287.73 | 139.53 | 571.17 | 334.67 | 0.7961 | 0.0032 |
| 3 | 10393573 | LGALS3BP | 236.49 | 418.28 | 334.32 | 288.04 | 974.20 | 424.80 | 1.2708 | 0.0003 |
| 3 | 10394778 | HPCAL1 | 212.48 | 242.56 | 270.87 | 245.49 | 334.67 | 292.10 | 0.4709 | 0.0304 |
| 3 | 10395259 | NAMPT | 893.11 | 818.60 | 802.78 | 855.58 | 1105.23 | 864.05 | 0.4389 | 0.0232 |
| 3 | 10398358 |  | 32.24 | 38.00 | 34.09 | 32.89 | 62.22 | 36.46 | 0.6333 | 0.0581 |
| 3 | 10398372 |  | 42.96 | 41.30 | 43.00 | 53.19 | 79.16 | 47.92 | 0.8470 | 0.0252 |
| 3 | 10398376 |  | 42.96 | 41.30 | 43.00 | 53.19 | 79.16 | 47.92 | 0.8470 | 0.0252 |
| 3 | 10398665 | TNFAIP2 | 97.36 | 170.01 | 255.94 | 119.14 | 247.27 | 400.49 | 0.5319 | 0.0392 |
| 3 | 10398907 | PLD4 | 177.10 | 304.96 | 255.48 | 181.98 | 548.34 | 314.86 | 0.8387 | 0.0041 |
| 3 | 10399005 | CRIP1 | 2435.31 | 2476.03 | 2960.61 | 2673.27 | 3223.94 | 3689.34 | 0.3822 | 0.0489 |
| 3 | 10399178 | CDCA7L | 111.49 | 175.78 | 167.73 | 133.85 | 235.31 | 200.03 | 0.4216 | 0.0208 |
| 3 | 10399202 | MACC1 | 35.36 | 33.77 | 34.97 | 36.92 | 41.49 | 43.18 | 0.2940 | 0.0399 |
| 3 | 10399540 | PQLC3 | 265.70 | 365.02 | 355.27 | 251.53 | 512.41 | 447.92 | 0.4910 | 0.0833 |

| Cluster | **Affymetrix transcript #** | Gene Symbol | Average levels of expression (arbitrary units) | | | | | | **Fold change**  **Tx_10d_ injured /Vh_10d_ injured** | |
| --- | --- | --- | --- | --- | --- | --- | --- | --- | --- | --- |
|  |  |  | **Vh_10d_ uninjured** | **Vh_10d_ injured** | **Vh_28d_ injured** | **Tx_10d_ uninjured** | **Tx_10d_ injured** | **Tx_28d_ injured** | **Log (2) Fold change** | **Adj. p-value** |
| 3 | 10401519 | NPC2 (includes EG:10577) | 1660.85 | 1945.34 | 1918.82 | 1695.63 | 2657.26 | 2057.92 | 0.4507 | 0.0040 |
| 3 | 10401956 |  | 58.68 | 62.27 | 77.89 | 91.37 | 102.52 | 94.07 | 0.6903 | 0.0141 |
| 3 | 10402268 | LGMN | 438.78 | 1735.04 | 1772.45 | 464.77 | 2983.80 | 2142.45 | 0.7805 | 0.0066 |
| 3 | 10403604 | LYST | 707.09 | 904.05 | 959.54 | 591.61 | 1132.57 | 1100.44 | 0.3289 | 0.0559 |
| 3 | 10403871 | AOAH | 75.44 | 416.23 | 386.48 | 84.34 | 733.61 | 537.17 | 0.8085 | 0.0663 |
| 3 | 10404606 | LY86 | 270.46 | 1016.49 | 1058.50 | 266.12 | 1789.79 | 1305.63 | 0.8116 | 0.0398 |
| 3 | 10405216 | SYK | 151.32 | 282.91 | 330.11 | 163.33 | 375.28 | 351.76 | 0.4056 | 0.0530 |
| 3 | 10406198 | FTL | 4145.16 | 5425.28 | 6154.86 | 4143.99 | 7139.56 | 6738.76 | 0.3973 | 0.0207 |
| 3 | 10406270 | GLRX | 265.02 | 236.94 | 250.37 | 256.40 | 325.40 | 240.34 | 0.4534 | 0.0536 |
| 3 | 10406905 | CCDC125 | 146.37 | 226.36 | 226.48 | 182.13 | 326.82 | 298.47 | 0.5120 | 0.0687 |
| 3 | 10406928 | CD180 | 195.11 | 658.17 | 808.22 | 249.81 | 1324.54 | 924.76 | 0.9269 | 0.0198 |
| 3 | 10407985 | GPR141 | 33.63 | 48.49 | 39.74 | 35.06 | 67.22 | 43.09 | 0.4960 | 0.0864 |
| 3 | 10408220 | HIST1H2AB/HIST1H2AE | 66.25 | 92.52 | 65.14 | 77.00 | 141.70 | 64.08 | 0.6372 | 0.0166 |
| 3 | 10408812 | MAK | 105.96 | 119.47 | 147.39 | 106.41 | 150.36 | 149.36 | 0.3283 | 0.0902 |
| 3 | 10409240 | SEMA4D | 206.50 | 266.33 | 287.26 | 198.34 | 340.79 | 332.34 | 0.3507 | 0.0880 |
| 3 | 10410124 | CTSL2 | 2047.67 | 3044.22 | 3724.57 | 2510.11 | 4093.70 | 4688.48 | 0.4223 | 0.0663 |
| 3 | 10411373 | HEXB | 940.38 | 2739.54 | 2512.87 | 1162.99 | 4027.62 | 3257.97 | 0.5515 | 0.0051 |
| 3 | 10411595 | Naip2 | 96.59 | 166.05 | 195.29 | 119.91 | 257.36 | 271.93 | 0.6203 | 0.0108 |
| 3 | 10411622 | NAIP | 54.87 | 113.95 | 142.07 | 62.75 | 192.57 | 192.20 | 0.7558 | 0.0059 |
| 3 | 10411958 | RNF180 | 221.80 | 278.91 | 317.84 | 229.54 | 367.17 | 334.73 | 0.3850 | 0.0579 |
| 3 | 10412211 | GZMA | 60.89 | 74.99 | 92.10 | 67.48 | 121.78 | 82.23 | 0.6716 | 0.0030 |
| 3 | 10413928 | C10orf128 | 116.91 | 616.70 | 512.21 | 185.50 | 1092.02 | 741.33 | 0.8279 | 0.0108 |
| 3 | 10414360 | LGALS3 | 402.90 | 1113.32 | 1276.41 | 599.45 | 1648.74 | 1467.45 | 0.5567 | 0.0317 |
| 3 | 10414548 | RNASE6 | 142.91 | 211.40 | 236.91 | 157.40 | 264.46 | 251.13 | 0.3209 | 0.0392 |
| 3 | 10416230 | TNFRSF10A | 132.40 | 167.98 | 145.17 | 186.24 | 249.62 | 161.91 | 0.5702 | 0.0119 |
| 3 | 10416340 | GFRA2 | 467.89 | 416.74 | 458.07 | 492.36 | 540.52 | 702.20 | 0.3730 | 0.0612 |
| 3 | 10416371 | LPAR6 | 506.85 | 664.44 | 806.31 | 656.31 | 927.36 | 907.67 | 0.4622 | 0.0741 |
| 3 | 10416411 | ESD | 934.08 | 1077.60 | 1040.70 | 1069.34 | 1348.49 | 1154.47 | 0.3141 | 0.0908 |
| 3 | 10416566 | Epsti1 | 134.64 | 268.95 | 294.40 | 183.81 | 473.99 | 348.43 | 0.8128 | 0.0003 |
| 3 | 10420030 | PSME2 | 626.83 | 587.66 | 680.59 | 709.95 | 836.52 | 800.01 | 0.5094 | 0.0005 |
| 3 | 10421488 | FNDC3A | 1043.73 | 1232.00 | 1214.66 | 993.16 | 1511.53 | 1311.38 | 0.2945 | 0.0979 |
| 3 | 10424676 | LY6E | 1045.21 | 1336.67 | 1341.78 | 1307.18 | 2488.08 | 1606.71 | 0.9735 | 0.0232 |
| 3 | 10424731 | GSDMD | 247.99 | 330.19 | 361.81 | 260.72 | 488.86 | 402.32 | 0.5668 | 0.0234 |
| 3 | 10425053 | NCF4 | 93.45 | 156.46 | 177.26 | 111.23 | 225.89 | 234.21 | 0.5291 | 0.0382 |
| 3 | 10425092 | CYTH4 | 282.23 | 466.77 | 585.82 | 281.24 | 666.75 | 681.60 | 0.5120 | 0.0486 |
| 3 | 10427235 | PRR13 | 408.75 | 468.98 | 532.93 | 365.70 | 625.59 | 580.41 | 0.4003 | 0.0430 |

| Cluster | **Affymetrix transcript #** | Gene Symbol | Average levels of expression (arbitrary units) | | | | | | **Fold change**  **Tx_10d_ injured /Vh_10d_ injured** | |
| --- | --- | --- | --- | --- | --- | --- | --- | --- | --- | --- |
|  |  |  | **Vh_10d_ uninjured** | **Vh_10d_ injured** | **Vh_28d_ injured** | **Tx_10d_ uninjured** | **Tx_10d_ injured** | **Tx_28d_ injured** | **Log (2) Fold change** | **Adj. p-value** |
| 3 | 10427628 | IL7R | 96.42 | 308.55 | 499.85 | 98.65 | 546.91 | 536.97 | 0.7698 | 0.0249 |
| 3 | 10427918 | FAM105A | 156.16 | 332.06 | 329.47 | 157.05 | 535.51 | 378.73 | 0.6846 | 0.0939 |
| 3 | 10428353 | LRP12 | 315.83 | 566.29 | 584.97 | 335.62 | 786.47 | 688.76 | 0.4920 | 0.0455 |
| 3 | 10430302 | CSF2RB | 286.17 | 352.45 | 417.88 | 317.79 | 510.54 | 545.84 | 0.5378 | 0.0476 |
| 3 | 10431424 | PLXNB2 | 269.57 | 405.39 | 398.06 | 299.00 | 526.31 | 427.54 | 0.3729 | 0.0825 |
| 3 | 10432986 | AAAS | 307.36 | 328.46 | 321.75 | 322.77 | 407.25 | 316.53 | 0.3049 | 0.0424 |
| 3 | 10433104 | ZNF385A | 239.75 | 255.79 | 258.47 | 254.65 | 311.35 | 289.39 | 0.2848 | 0.0841 |
| 3 | 10434778 | RTP4 | 561.71 | 452.04 | 350.17 | 630.67 | 1344.83 | 558.55 | 1.7211 | 0.0003 |
| 3 | 10435457 | PARP9 | 271.38 | 339.41 | 328.41 | 288.50 | 495.57 | 377.55 | 0.5409 | 0.0108 |
| 3 | 10435704 | CD80 (includes EG:12519) | 82.64 | 147.32 | 167.58 | 121.92 | 209.68 | 186.48 | 0.5016 | 0.0354 |
| 3 | 10435907 | CD200R1 | 50.08 | 106.29 | 112.52 | 58.54 | 150.80 | 139.60 | 0.4888 | 0.0849 |
| 3 | 10435920 |  | 51.73 | 195.03 | 184.69 | 57.25 | 365.86 | 259.86 | 0.8806 | 0.0051 |
| 3 | 10436209 | CBLB | 502.38 | 723.64 | 702.59 | 561.40 | 1076.37 | 777.58 | 0.5726 | 0.0417 |
| 3 | 10437023 | MORC3 | 780.54 | 818.45 | 776.20 | 646.60 | 1004.17 | 871.05 | 0.2959 | 0.0461 |
| 3 | 10437224 | MX1 | 86.41 | 73.08 | 75.27 | 87.08 | 136.16 | 99.06 | 0.9366 | 0.0246 |
| 3 | 10437272 | NLRC3 | 116.63 | 127.40 | 147.68 | 116.41 | 162.56 | 170.35 | 0.3509 | 0.0391 |
| 3 | 10439249 | PARP14 | 457.87 | 663.52 | 614.17 | 414.56 | 859.42 | 787.81 | 0.3728 | 0.0997 |
| 3 | 10439268 |  | 188.17 | 264.28 | 262.35 | 240.86 | 404.54 | 331.35 | 0.6165 | 0.0004 |
| 3 | 10441233 | MX1 | 71.96 | 74.24 | 73.18 | 69.15 | 126.46 | 99.43 | 0.7341 | 0.0138 |
| 3 | 10442596 | MSRB1 | 485.31 | 531.10 | 525.35 | 498.15 | 823.26 | 595.82 | 0.6124 | 0.0112 |
| 3 | 10443980 | MYO1F | 151.49 | 568.73 | 661.72 | 197.10 | 856.26 | 780.43 | 0.5895 | 0.0127 |
| 3 | 10444244 | TAP1 | 168.00 | 198.35 | 225.73 | 191.37 | 297.53 | 241.44 | 0.5770 | 0.0022 |
| 3 | 10444258 | PSMB8 | 539.03 | 609.85 | 661.24 | 627.71 | 992.49 | 860.86 | 0.7050 | 0.0051 |
| 3 | 10444658 | CLIC1 | 725.06 | 1150.65 | 1067.36 | 761.76 | 1593.44 | 1288.42 | 0.4719 | 0.0066 |
| 3 | 10444814 | H2-Q5 | 102.28 | 115.74 | 137.03 | 104.99 | 169.36 | 156.18 | 0.5534 | 0.0630 |
| 3 | 10444830 | HLA-B | 1075.92 | 1310.16 | 1518.37 | 1179.95 | 1938.58 | 1837.78 | 0.5707 | 0.0900 |
| 3 | 10445119 | HLA-F | 267.82 | 361.63 | 383.03 | 365.77 | 512.66 | 468.83 | 0.5035 | 0.0048 |
| 3 | 10445774 | 9830107B12Rik (includes others) | 169.83 | 229.83 | 290.85 | 170.89 | 334.02 | 379.62 | 0.5267 | 0.0189 |
| 3 | 10446253 | VAV1 | 112.26 | 237.87 | 228.04 | 122.59 | 364.37 | 280.05 | 0.6111 | 0.0280 |
| 3 | 10447429 | Gm4832 | 376.11 | 413.01 | 442.75 | 416.34 | 541.93 | 526.65 | 0.3896 | 0.0455 |
| 3 | 10447591 | FTL | 3425.05 | 4403.38 | 4966.15 | 3473.52 | 5997.81 | 5722.79 | 0.4435 | 0.0259 |
| 3 | 10448208 | ZNF548 | 41.70 | 37.55 | 44.41 | 40.86 | 52.69 | 46.42 | 0.4723 | 0.0907 |
| 3 | 10450075 | HLA-C | 1223.91 | 1459.12 | 1501.88 | 1485.68 | 2097.73 | 1910.76 | 0.5399 | 0.0947 |
| 3 | 10450484 | AIF1 (includes EG:11629) | 279.24 | 913.13 | 821.29 | 271.39 | 1377.95 | 1039.27 | 0.5945 | 0.0536 |
| 3 | 10450675 | H2-T24 | 177.19 | 219.81 | 222.86 | 180.74 | 341.76 | 240.33 | 0.6265 | 0.0040 |

| Cluster | **Affymetrix transcript #** | | Gene Symbol | Average levels of expression (arbitrary units) | | | | | | **Fold change**  **Tx_10d_ injured /Vh_10d_ injured** | |
| --- | --- | --- | --- | --- | --- | --- | --- | --- | --- | --- | --- |
|  | |  |  | **Vh_10d_ uninjured** | **Vh_10d_ injured** | **Vh_28d_ injured** | **Tx_10d_ uninjured** | **Tx_10d_ injured** | **Tx_28d_ injured** | **Log (2) Fold change** | **Adj. p-value** |
| 3 | | 10450694 | H2-T10/H2-T22 | 372.85 | 441.69 | 504.00 | 491.33 | 603.16 | 521.56 | 0.4560 | 0.0802 |
| 3 | | 10451287 | ISG15 | 369.66 | 348.45 | 390.91 | 337.71 | 653.31 | 463.72 | 0.9049 | 0.0058 |
| 3 | | 10452978 | Gm6548 | 149.61 | 211.68 | 206.02 | 210.50 | 320.25 | 272.48 | 0.5973 | 0.0249 |
| 3 | | 10452980 | EIF2AK2 | 251.02 | 362.26 | 294.36 | 303.23 | 733.87 | 426.47 | 1.0194 | 0.0131 |
| 3 | | 10455533 | EIF1AY | 550.96 | 814.52 | 673.79 | 614.21 | 944.42 | 729.70 | 0.2144 | 0.0812 |
| 3 | | 10455957 |  | 75.29 | 78.01 | 70.61 | 88.81 | 107.65 | 85.58 | 0.4791 | 0.0579 |
| 3 | | 10455961 | Iigp1 | 263.88 | 249.58 | 179.32 | 319.01 | 439.15 | 248.68 | 0.8627 | 0.0940 |
| 3 | | 10457225 | MAP3K8 | 217.47 | 308.71 | 336.94 | 281.92 | 412.42 | 349.55 | 0.4153 | 0.0314 |
| 3 | | 10458314 | TMEM173 | 167.25 | 258.54 | 277.29 | 204.02 | 406.60 | 305.26 | 0.6460 | 0.0241 |
| 3 | | 10458999 | FBN2 (includes EG:14119) | 219.58 | 590.37 | 309.41 | 232.13 | 797.74 | 403.62 | 0.4410 | 0.0097 |
| 3 | | 10459071 | C5orf62 | 167.29 | 357.53 | 309.21 | 214.38 | 507.43 | 333.31 | 0.4973 | 0.0166 |
| 3 | | 10460118 | SOCS6 | 430.76 | 540.42 | 513.72 | 469.37 | 844.75 | 602.55 | 0.6428 | 0.0407 |
| 3 | | 10460237 | UNC93B1 | 208.26 | 516.36 | 530.35 | 239.18 | 737.46 | 640.78 | 0.5190 | 0.0723 |
| 3 | | 10461558 | SLC15A3 | 171.45 | 574.44 | 618.02 | 213.29 | 1007.60 | 762.79 | 0.8087 | 0.0161 |
| 3 | | 10461594 | Ms4a4b (includes others) | 83.76 | 156.21 | 138.78 | 84.17 | 367.04 | 198.93 | 1.2366 | 0.0001 |
| 3 | | 10461605 | Ms4a4b (includes others) | 37.90 | 46.68 | 58.04 | 38.54 | 67.46 | 64.93 | 0.5132 | 0.0165 |
| 3 | | 10461622 | Ms4a6b | 199.42 | 510.25 | 376.03 | 195.79 | 1134.98 | 661.09 | 1.1658 | 0.0382 |
| 3 | | 10461636 |  | 29.15 | 41.87 | 41.48 | 30.21 | 66.85 | 46.70 | 0.6712 | 0.0011 |
| 3 | | 10461765 | LPXN | 149.27 | 203.93 | 195.69 | 174.76 | 260.54 | 259.03 | 0.3611 | 0.0610 |
| 3 | | 10461856 | GNA14 | 224.19 | 257.74 | 272.57 | 296.18 | 396.48 | 304.28 | 0.6185 | 0.0464 |
| 3 | | 10462140 | DOCK8 | 153.44 | 343.17 | 404.39 | 196.13 | 531.07 | 448.19 | 0.6233 | 0.0006 |
| 3 | | 10462390 | CD274 | 218.40 | 232.33 | 283.58 | 237.01 | 303.40 | 308.31 | 0.3917 | 0.0989 |
| 3 | | 10462499 | A1CF | 20.40 | 22.98 | 22.90 | 19.49 | 28.69 | 23.90 | 0.3259 | 0.0943 |
| 3 | | 10462618 | I830012O16Rik/Ifit3 | 1316.36 | 823.89 | 1003.85 | 1405.53 | 2192.54 | 1863.10 | 1.4223 | 0.0001 |
| 3 | | 10463070 | ENTPD1 | 538.87 | 936.83 | 1153.78 | 855.99 | 1515.45 | 1370.93 | 0.6951 | 0.0003 |
| 3 | | 10464529 | TCIRG1 | 350.64 | 570.72 | 599.56 | 339.95 | 784.49 | 681.37 | 0.4578 | 0.0191 |
| 3 | | 10466210 | MS4A6A | 101.04 | 541.62 | 457.14 | 161.72 | 1211.77 | 693.01 | 1.1572 | 0.0022 |
| 3 | | 10466248 | STX3 | 300.45 | 349.52 | 342.01 | 254.36 | 455.36 | 366.69 | 0.3686 | 0.0650 |
| 3 | | 10466314 |  | 78.73 | 72.58 | 78.88 | 81.77 | 113.88 | 78.71 | 0.6096 | 0.0398 |
| 3 | | 10466374 | TLE4 | 177.59 | 173.61 | 188.00 | 170.09 | 225.72 | 202.66 | 0.3782 | 0.0852 |
| 3 | | 10466938 | PLGRKT | 266.47 | 310.50 | 361.16 | 346.06 | 457.54 | 359.85 | 0.5445 | 0.0359 |
| 3 | | 10467139 | LIPA | 1176.65 | 1469.12 | 2021.25 | 1163.24 | 2276.94 | 2601.19 | 0.6315 | 0.0516 |
| 3 | | 10467508 | BLNK | 142.86 | 437.52 | 492.01 | 140.99 | 739.33 | 607.42 | 0.7344 | 0.0093 |

| Cluster | **Affymetrix transcript #** | Gene Symbol | Average levels of expression (arbitrary units) | | | | | | **Fold change**  **Tx_10d_ injured /Vh_10d_ injured** | |
| --- | --- | --- | --- | --- | --- | --- | --- | --- | --- | --- |
|  |  |  | **Vh_10d_ uninjured** | **Vh_10d_ injured** | **Vh_28d_ injured** | **Tx_10d_ uninjured** | **Tx_10d_ injured** | **Tx_28d_ injured** | **Log (2) Fold change** | **Adj. p-value** |
| 3 | 10471912 | KYNU | 67.94 | 196.22 | 221.20 | 90.71 | 305.26 | 238.99 | 0.6167 | 0.0697 |
| 3 | 10471929 | ARHGAP15 | 128.13 | 328.99 | 400.42 | 140.71 | 549.24 | 511.11 | 0.7265 | 0.0774 |
| 3 | 10472289 | TANK | 396.89 | 496.48 | 506.31 | 362.62 | 673.06 | 587.39 | 0.4368 | 0.0131 |
| 3 | 10473125 | ITGA4 | 368.74 | 651.90 | 736.55 | 374.83 | 1026.77 | 958.52 | 0.6537 | 0.0094 |
| 3 | 10474199 |  | 1732.63 | 1936.31 | 2081.25 | 1894.78 | 2305.10 | 2296.53 | 0.2519 | 0.0970 |
| 3 | 10474467 | MUC15 | 38.41 | 42.31 | 37.90 | 38.35 | 65.16 | 39.49 | 0.5808 | 0.0392 |
| 3 | 10474524 | Olfr1317/Olfr1318 | 36.04 | 45.33 | 41.93 | 42.27 | 66.29 | 49.01 | 0.5083 | 0.0884 |
| 3 | 10475517 | C15orf48 | 58.76 | 68.53 | 69.75 | 61.84 | 123.23 | 73.07 | 0.7317 | 0.0897 |
| 3 | 10475708 | BLVRA | 190.42 | 420.21 | 434.00 | 254.23 | 610.53 | 484.13 | 0.5322 | 0.0417 |
| 3 | 10477250 | HCK | 157.49 | 204.99 | 234.67 | 168.96 | 275.42 | 240.97 | 0.4237 | 0.0486 |
| 3 | 10478594 | CTSA | 1048.57 | 1580.44 | 1577.44 | 994.18 | 2154.33 | 1702.18 | 0.4456 | 0.0066 |
| 3 | 10478875 | RNF114 | 365.70 | 391.48 | 443.43 | 359.15 | 492.83 | 417.21 | 0.3305 | 0.0489 |
| 3 | 10480699 | DPP7 | 210.14 | 352.40 | 354.65 | 239.49 | 493.12 | 350.98 | 0.4903 | 0.0077 |
| 3 | 10482059 | GGTA1P | 508.15 | 529.02 | 597.61 | 512.21 | 681.27 | 713.14 | 0.3636 | 0.0321 |
| 3 | 10482517 | NMI | 469.10 | 519.18 | 524.28 | 563.98 | 732.06 | 617.50 | 0.4988 | 0.0317 |
| 3 | 10483110 | IFIH1 | 457.22 | 429.45 | 452.57 | 478.52 | 761.00 | 654.61 | 0.8325 | 0.0022 |
| 3 | 10483249 | GALNT3 | 157.33 | 322.71 | 236.72 | 145.08 | 535.56 | 309.70 | 0.7401 | 0.0015 |
| 3 | 10484261 | ITGA4 | 58.86 | 65.27 | 85.17 | 61.91 | 86.04 | 95.31 | 0.3947 | 0.0392 |
| 3 | 10485718 | MUC15 | 111.33 | 96.60 | 84.84 | 74.66 | 218.21 | 104.20 | 1.1709 | 0.0077 |
| 3 | 10487208 | ATP8B4 | 105.96 | 294.51 | 312.13 | 114.55 | 561.25 | 410.35 | 0.9158 | 0.0112 |
| 3 | 10487588 | IL1A | 59.33 | 208.34 | 184.67 | 54.61 | 386.02 | 314.04 | 0.9116 | 0.0317 |
| 3 | 10488237 | SNX5 | 1984.27 | 2511.89 | 2528.92 | 1962.79 | 3107.23 | 2840.98 | 0.3077 | 0.0646 |
| 3 | 10489107 | SAMHD1 | 1014.25 | 1050.35 | 1057.15 | 1073.38 | 1502.20 | 1324.48 | 0.5140 | 0.0124 |
| 3 | 10489204 | TGM2) | 255.75 | 318.94 | 297.19 | 298.43 | 443.47 | 372.79 | 0.4840 | 0.0466 |
| 3 | 10489246 | MAFB | 133.08 | 346.67 | 347.92 | 165.02 | 483.49 | 371.62 | 0.4838 | 0.0429 |
| 3 | 10489569 | PLTP | 790.73 | 1004.76 | 1255.78 | 748.92 | 1594.53 | 1725.97 | 0.6651 | 0.0812 |
| 3 | 10489759 | SULF2 | 366.72 | 643.26 | 546.51 | 448.89 | 1001.43 | 674.53 | 0.6325 | 0.0840 |
| 3 | 10490126 | RPS29 | 8219.65 | 7890.89 | 8668.87 | 8491.44 | 9432.30 | 8770.56 | 0.2579 | 0.0398 |
| 3 | 10490150 | ZBP1 | 182.48 | 234.46 | 197.80 | 199.63 | 460.12 | 270.93 | 0.9729 | 0.0001 |
| 3 | 10490212 | CTSZ | 925.01 | 1647.38 | 1795.35 | 1146.14 | 2334.22 | 2118.28 | 0.5033 | 0.0051 |
| 3 | 10490838 | FABP5 | 1424.87 | 2615.01 | 2812.72 | 1454.15 | 3829.73 | 3451.71 | 0.5439 | 0.0119 |
| 3 | 10491272 | GPR160 | 128.72 | 133.07 | 148.74 | 116.25 | 181.30 | 165.00 | 0.4561 | 0.0362 |
| 3 | 10492815 | TMEM154 | 109.53 | 134.07 | 146.57 | 121.10 | 188.08 | 177.89 | 0.4753 | 0.0092 |
| 3 | 10494978 | PTPN22 | 102.89 | 162.82 | 234.27 | 104.33 | 277.75 | 294.59 | 0.7691 | 0.0051 |
| 3 | 10495316 | PSRC1 | 189.73 | 212.22 | 177.97 | 197.37 | 287.96 | 195.89 | 0.4380 | 0.0084 |
| 3 | 10496569 | GBP7 | 530.66 | 485.12 | 528.29 | 480.22 | 677.34 | 735.69 | 0.4914 | 0.0834 |
| 3 | 10496580 | GBP4 | 347.40 | 384.90 | 351.93 | 379.40 | 664.62 | 520.55 | 0.7778 | 0.0066 |

| Cluster | **Affymetrix transcript #** | Gene Symbol | Average levels of expression (arbitrary units) | | | | | | **Fold change**  **Tx_10d_ injured /Vh_10d_ injured** | |
| --- | --- | --- | --- | --- | --- | --- | --- | --- | --- | --- |
|  |  |  | **Vh_10d_ uninjured** | **Vh_10d_ injured** | **Vh_28d_ injured** | **Tx_10d_ uninjured** | **Tx_10d_ injured** | **Tx_28d_ injured** | **Log (2) Fold change** | **Adj. p-value** |
| 3 | 10496756 | MCOLN3 | 105.15 | 259.94 | 252.34 | 101.93 | 435.96 | 330.94 | 0.6957 | 0.0530 |
| 3 | 10497345 | Gm9733 | 37.07 | 38.49 | 41.46 | 35.00 | 61.71 | 46.42 | 0.6525 | 0.0009 |
| 3 | 10497349 | Sirpb1a (includes others) | 154.78 | 401.44 | 315.39 | 184.09 | 748.90 | 509.68 | 0.8522 | 0.0122 |
| 3 | 10497356 | Sirpb1a (includes others) | 89.10 | 270.74 | 224.52 | 101.25 | 517.71 | 334.06 | 0.8520 | 0.0749 |
| 3 | 10497358 | Sirpb1a (includes others) | 92.00 | 274.11 | 202.57 | 101.78 | 557.10 | 344.61 | 1.0007 | 0.0044 |
| 3 | 10497364 | Sirpb1a (includes others) | 139.69 | 359.38 | 288.40 | 165.36 | 688.42 | 464.47 | 0.8941 | 0.0068 |
| 3 | 10497372 | Gm5150 | 100.73 | 131.45 | 162.08 | 104.73 | 190.05 | 150.65 | 0.5210 | 0.0822 |
| 3 | 10497673 | ZMAT3 | 128.80 | 175.47 | 144.43 | 135.96 | 233.96 | 171.20 | 0.4060 | 0.0349 |
| 3 | 10500335 | FCGR1A | 216.68 | 401.64 | 365.27 | 226.15 | 744.11 | 515.39 | 0.8735 | 0.0040 |
| 3 | 10500610 | FAM46C | 279.13 | 397.76 | 363.12 | 298.20 | 577.83 | 398.14 | 0.5319 | 0.0261 |
| 3 | 10501608 | VCAM1 | 411.11 | 512.59 | 482.47 | 427.38 | 719.43 | 630.97 | 0.4984 | 0.0792 |
| 3 | 10502359 | DAPP1 | 98.11 | 165.50 | 189.02 | 134.66 | 242.47 | 239.89 | 0.5543 | 0.0867 |
| 3 | 10502791 | IFI44 | 329.02 | 310.00 | 237.65 | 389.50 | 605.36 | 334.46 | 1.0261 | 0.0113 |
| 3 | 10503098 | LYN | 197.59 | 528.28 | 559.02 | 270.97 | 835.04 | 616.72 | 0.6565 | 0.0739 |
| 3 | 10503134 | SDCBP | 2259.01 | 4180.94 | 3959.20 | 2094.68 | 5204.85 | 4623.94 | 0.3192 | 0.0596 |
| 3 | 10503259 | TP53INP1 | 724.01 | 846.55 | 765.74 | 756.89 | 1426.15 | 869.35 | 0.7514 | 0.0059 |
| 3 | 10504582 | 1300002K09Rik | 180.99 | 223.31 | 265.36 | 199.94 | 287.26 | 290.30 | 0.3610 | 0.0813 |
| 3 | 10505489 | PAPPA | 133.18 | 193.56 | 161.60 | 152.73 | 302.13 | 150.47 | 0.6294 | 0.0040 |
| 3 | 10508069 | FTL | 4000.09 | 5191.87 | 5954.86 | 4125.82 | 6710.05 | 6453.38 | 0.3712 | 0.0167 |
| 3 | 10508074 | CSF3R | 149.36 | 276.33 | 270.01 | 176.40 | 370.73 | 318.74 | 0.4190 | 0.0241 |
| 3 | 10508663 | LAPTM5 | 306.40 | 1485.40 | 1587.84 | 454.33 | 2472.18 | 1986.63 | 0.7402 | 0.0518 |
| 3 | 10508734 | PTAFR | 129.49 | 257.88 | 300.77 | 129.77 | 364.05 | 304.52 | 0.4847 | 0.0523 |
| 3 | 10510150 | Gm13152 | 43.53 | 44.13 | 55.50 | 40.18 | 79.21 | 66.31 | 0.7713 | 0.0232 |
| 3 | 10510178 | ZNF41 | 213.05 | 206.90 | 216.93 | 205.94 | 266.93 | 241.93 | 0.3602 | 0.0925 |
| 3 | 10510215 | ZNF41 | 343.85 | 365.32 | 339.92 | 298.15 | 516.29 | 374.40 | 0.5039 | 0.0348 |
| 3 | 10512067 | DDX58 | 584.99 | 587.13 | 598.82 | 612.37 | 983.22 | 762.45 | 0.7559 | 0.0029 |
| 3 | 10512999 | AI427809 | 184.84 | 403.03 | 454.34 | 225.17 | 648.08 | 620.10 | 0.6580 | 0.0417 |
| 3 | 10513774 |  | 34.80 | 49.98 | 37.31 | 43.51 | 109.18 | 39.32 | 1.0776 | 0.0043 |
| 3 | 10514133 | TTC39B | 178.67 | 314.04 | 342.99 | 218.26 | 435.48 | 354.01 | 0.4695 | 0.0306 |
| 3 | 10514275 | PTPLAD2 | 141.05 | 266.92 | 272.17 | 142.16 | 449.72 | 342.60 | 0.7230 | 0.0422 |
| 3 | 10516932 | SESN2 | 127.67 | 166.40 | 146.76 | 141.90 | 200.81 | 168.85 | 0.2761 | 0.0812 |
| 3 | 10516966 | C1orf38 | 186.83 | 275.14 | 314.01 | 231.01 | 385.94 | 315.10 | 0.4787 | 0.0399 |
| 3 | 10517165 | CD52 | 167.34 | 534.47 | 665.70 | 222.75 | 1257.97 | 709.73 | 1.1750 | 0.0122 |

| Cluster | **Affymetrix transcript #** | Gene Symbol | Average levels of expression (arbitrary units) | | | | | | **Fold change**  **Tx_10d_ injured /Vh_10d_ injured** | |
| --- | --- | --- | --- | --- | --- | --- | --- | --- | --- | --- |
|  |  |  | **Vh_10d_ uninjured** | **Vh_10d_ injured** | **Vh_28d_ injured** | **Tx_10d_ uninjured** | **Tx_10d_ injured** | **Tx_28d_ injured** | **Log (2) Fold change** | **Adj. p-value** |
| 3 | 10517508 | C1QB | 306.84 | 1057.81 | 1092.31 | 394.78 | 1674.58 | 1245.48 | 0.6511 | 0.0757 |
| 3 | 10518069 | EFHD2 | 443.66 | 687.23 | 695.38 | 452.88 | 932.49 | 736.46 | 0.4389 | 0.0132 |
| 3 | 10518300 | TNFRSF1B | 214.33 | 383.31 | 425.37 | 240.49 | 481.65 | 454.90 | 0.3292 | 0.0650 |
| 3 | 10518335 | ZNF41 | 997.13 | 973.21 | 1017.52 | 923.78 | 1322.52 | 1099.02 | 0.4308 | 0.0693 |
| 3 | 10519555 | Abcb1b | 185.65 | 167.37 | 207.05 | 176.99 | 272.23 | 273.42 | 0.6961 | 0.0045 |
| 3 | 10519857 | HGF | 62.39 | 184.89 | 182.66 | 82.20 | 295.33 | 244.26 | 0.6833 | 0.0050 |
| 3 | 10520271 | 2900005J15Rik | 125.05 | 126.51 | 134.42 | 137.28 | 151.13 | 153.93 | 0.2550 | 0.0889 |
| 3 | 10521134 | RPS29 | 6961.25 | 6637.99 | 7270.16 | 7141.52 | 8039.58 | 7438.81 | 0.2774 | 0.0559 |
| 3 | 10524621 | Oasl2 | 304.03 | 273.54 | 239.16 | 344.25 | 647.52 | 391.25 | 1.3098 | 0.0011 |
| 3 | 10525158 | Oas1b | 99.18 | 99.52 | 94.60 | 106.78 | 148.89 | 116.15 | 0.6002 | 0.0335 |
| 3 | 10525439 | P2RX4 (includes EG:18438) | 306.33 | 668.54 | 730.42 | 291.14 | 1085.19 | 895.09 | 0.6995 | 0.0138 |
| 3 | 10527638 | ALOX5AP | 314.16 | 678.44 | 630.36 | 413.42 | 1283.48 | 896.82 | 0.8983 | 0.0131 |
| 3 | 10530145 | TLR1 | 95.08 | 227.05 | 304.69 | 104.83 | 455.02 | 372.97 | 0.9050 | 0.0295 |
| 3 | 10530151 | TLR6 | 124.17 | 191.21 | 177.61 | 139.75 | 270.66 | 228.15 | 0.4955 | 0.0008 |
| 3 | 10530998 | TMPRSS11F | 35.80 | 51.68 | 45.19 | 41.61 | 73.04 | 44.67 | 0.4840 | 0.0614 |
| 3 | 10531415 | CXCL10 | 174.84 | 665.40 | 525.37 | 215.67 | 1119.50 | 718.73 | 0.7332 | 0.0532 |
| 3 | 10531737 | HPSE | 69.24 | 498.30 | 488.76 | 95.33 | 912.57 | 626.41 | 0.8778 | 0.0022 |
| 3 | 10531952 | Abcg3 | 69.84 | 100.77 | 116.03 | 75.15 | 134.62 | 132.58 | 0.4140 | 0.0355 |
| 3 | 10531972 | Gbp8 | 106.66 | 151.57 | 143.73 | 114.33 | 255.65 | 176.06 | 0.7418 | 0.0073 |
| 3 | 10531994 | Gbp6 | 404.64 | 348.78 | 349.21 | 392.88 | 599.12 | 564.87 | 0.7864 | 0.0362 |
| 3 | 10532157 | TMED5 | 234.17 | 228.80 | 199.59 | 235.55 | 284.19 | 202.87 | 0.3117 | 0.0954 |
| 3 | 10532744 | SELPLG | 230.82 | 355.43 | 388.34 | 253.25 | 495.01 | 406.86 | 0.4712 | 0.0321 |
| 3 | 10533198 | OAS2 | 200.05 | 274.61 | 187.71 | 200.52 | 531.68 | 241.64 | 0.9669 | 0.0195 |
| 3 | 10533246 | OAS1 | 152.15 | 205.45 | 174.21 | 189.52 | 441.67 | 228.53 | 1.1166 | 0.0000 |
| 3 | 10533256 | OAS1 | 202.07 | 216.65 | 236.83 | 241.77 | 460.10 | 300.70 | 1.0783 | 0.0025 |
| 3 | 10533304 | TRAFD1 | 612.23 | 638.92 | 698.39 | 583.00 | 809.16 | 730.89 | 0.3393 | 0.0687 |
| 3 | 10534202 | NCF1 | 163.73 | 261.00 | 270.84 | 183.03 | 345.07 | 272.12 | 0.3989 | 0.0822 |
| 3 | 10534909 | SP110 | 218.66 | 318.74 | 327.50 | 221.87 | 546.48 | 377.07 | 0.7683 | 0.0108 |
| 3 | 10537102 | EXOC4 | 563.45 | 584.36 | 574.34 | 564.01 | 732.51 | 588.90 | 0.3333 | 0.0813 |
| 3 | 10537157 | Akr1b10 | 182.44 | 214.87 | 237.53 | 239.45 | 310.53 | 267.75 | 0.5218 | 0.0584 |
| 3 | 10537410 | TBXAS1 | 142.77 | 329.12 | 403.82 | 167.22 | 505.27 | 426.84 | 0.6007 | 0.0348 |
| 3 | 10538126 | GIMAP4 | 361.24 | 382.56 | 536.27 | 411.00 | 565.43 | 596.07 | 0.5800 | 0.0701 |
| 3 | 10538590 | HERC6 | 652.97 | 526.48 | 486.05 | 511.86 | 935.27 | 721.86 | 0.8382 | 0.0015 |
| 3 | 10540542 |  | 579.25 | 575.29 | 734.35 | 685.48 | 770.17 | 784.53 | 0.4111 | 0.0607 |
| 3 | 10540795 | IRAK2 | 179.17 | 193.12 | 214.13 | 184.81 | 247.75 | 250.50 | 0.3616 | 0.0583 |
| 3 | 10541307 | USP18 | 245.21 | 209.05 | 224.51 | 311.15 | 435.26 | 292.14 | 1.0322 | 0.0006 |

| Cluster | **Affymetrix transcript #** | Gene Symbol | Average levels of expression (arbitrary units) | | | | | | **Fold change**  **Tx_10d_ injured /Vh_10d_ injured** | |
| --- | --- | --- | --- | --- | --- | --- | --- | --- | --- | --- |
|  |  |  | **Vh_10d_ uninjured** | **Vh_10d_ injured** | **Vh_28d_ injured** | **Tx_10d_ uninjured** | **Tx_10d_ injured** | **Tx_28d_ injured** | **Log (2) Fold change** | **Adj. p-value** |
| 3 | 10542156 | CLEC2D | 473.16 | 804.15 | 885.10 | 631.95 | 1252.75 | 960.08 | 0.6589 | 0.0670 |
| 3 | 10542164 | CLEC12A | 84.22 | 415.59 | 591.80 | 104.48 | 736.50 | 695.01 | 0.8189 | 0.0309 |
| 3 | 10542181 | CLEC9A | 89.04 | 192.06 | 190.49 | 110.62 | 305.84 | 263.99 | 0.6575 | 0.0914 |
| 3 | 10542205 | Klre1 | 23.36 | 24.64 | 25.67 | 23.61 | 28.63 | 27.83 | 0.2180 | 0.0929 |
| 3 | 10543781 | mir-29 | 14.65 | 15.00 | 15.50 | 14.61 | 17.99 | 15.49 | 0.2586 | 0.0569 |
| 3 | 10544133 | PARP12 | 461.40 | 520.23 | 542.99 | 475.03 | 780.47 | 707.88 | 0.5783 | 0.0039 |
| 3 | 10544588 | GIMAP5 | 89.81 | 110.61 | 151.03 | 102.47 | 159.92 | 180.27 | 0.5061 | 0.0867 |
| 3 | 10544732 | SKAP2 | 1097.87 | 1414.95 | 1397.69 | 1031.39 | 1790.60 | 1479.61 | 0.3374 | 0.0492 |
| 3 | 10545101 | HPGDS | 186.14 | 910.59 | 989.13 | 221.10 | 1479.28 | 1301.57 | 0.7040 | 0.0299 |
| 3 | 10547769 | PTPN6 | 263.43 | 410.58 | 499.13 | 312.49 | 552.26 | 517.94 | 0.4221 | 0.0529 |
| 3 | 10548345 | KLRC4-KLRK1/KLRK1 | 53.82 | 55.51 | 64.09 | 53.62 | 77.96 | 82.07 | 0.4869 | 0.0315 |
| 3 | 10548375 | CLEC7A | 160.46 | 773.05 | 896.95 | 185.67 | 1219.35 | 1385.98 | 0.6970 | 0.0914 |
| 3 | 10548409 | KLRC1 | 29.13 | 29.64 | 30.40 | 28.96 | 42.57 | 37.08 | 0.4971 | 0.0305 |
| 3 | 10548422 | Klri2 | 18.68 | 19.35 | 20.83 | 18.06 | 25.03 | 24.88 | 0.3484 | 0.0954 |
| 3 | 10548504 | Klra21 | 58.47 | 56.34 | 67.12 | 58.48 | 74.94 | 75.90 | 0.4042 | 0.0209 |
| 3 | 10548552 | Klra2 | 82.25 | 162.01 | 145.78 | 89.84 | 257.90 | 205.74 | 0.6579 | 0.0066 |
| 3 | 10548905 | EPS8 | 428.48 | 532.42 | 509.08 | 526.30 | 658.52 | 570.02 | 0.3065 | 0.0614 |
| 3 | 10549495 | RPS29 | 7217.92 | 6925.36 | 7548.40 | 7420.34 | 8303.54 | 7797.96 | 0.2629 | 0.0536 |
| 3 | 10552406 | NKG7 | 106.71 | 115.08 | 141.06 | 110.95 | 169.06 | 166.61 | 0.5590 | 0.0051 |
| 3 | 10554945 | PRCP | 428.78 | 664.02 | 626.45 | 436.76 | 863.44 | 722.60 | 0.3823 | 0.0823 |
| 3 | 10555027 | GAB2 | 552.27 | 838.33 | 925.08 | 579.96 | 1059.66 | 880.90 | 0.3414 | 0.0604 |
| 3 | 10555041 | ALG8 | 358.90 | 503.20 | 418.56 | 420.93 | 704.43 | 499.35 | 0.4707 | 0.0559 |
| 3 | 10555862 | TRIM6-TRIM34 | 528.20 | 487.20 | 543.11 | 535.60 | 740.25 | 752.64 | 0.6269 | 0.0759 |
| 3 | 10557862 | ITGAM | 134.63 | 300.41 | 271.65 | 161.88 | 439.54 | 360.66 | 0.5376 | 0.0317 |
| 3 | 10559446 | LILRB3 | 241.50 | 427.88 | 525.34 | 286.37 | 668.74 | 570.73 | 0.6257 | 0.0187 |
| 3 | 10559486 | LAIR1 | 190.07 | 554.64 | 551.95 | 222.04 | 867.22 | 751.82 | 0.6336 | 0.0167 |
| 3 | 10559649 | COX6B2 | 371.05 | 329.75 | 280.61 | 365.41 | 576.57 | 342.13 | 0.7999 | 0.0486 |
| 3 | 10561306 | PLD3 | 505.45 | 1142.26 | 1093.77 | 435.65 | 1656.07 | 1261.19 | 0.5341 | 0.0577 |
| 3 | 10561920 | HCST | 198.10 | 284.94 | 330.39 | 215.02 | 466.39 | 332.80 | 0.6573 | 0.0569 |
| 3 | 10562192 | FXYD5 | 880.90 | 1560.65 | 1649.63 | 1324.09 | 2743.13 | 1982.10 | 0.8046 | 0.0000 |
| 3 | 10562709 | Cd33 | 252.10 | 516.97 | 521.88 | 281.91 | 776.00 | 609.60 | 0.5636 | 0.0074 |
| 3 | 10563178 | CD37 | 201.30 | 295.59 | 363.61 | 216.41 | 411.93 | 356.83 | 0.4664 | 0.0181 |
| 3 | 10563295 | FTL | 4306.05 | 5704.56 | 6399.87 | 4262.51 | 7386.02 | 6936.10 | 0.3735 | 0.0407 |
| 3 | 10565018 | IQGAP1 | 980.22 | 1530.03 | 1509.94 | 1023.77 | 1966.71 | 1784.30 | 0.3613 | 0.0160 |
| 3 | 10565990 | Art2a-ps/Art2b | 76.13 | 132.18 | 132.29 | 85.62 | 283.69 | 181.86 | 0.9989 | 0.0348 |

| Cluster | **Affymetrix transcript #** | | Gene Symbol | Average levels of expression (arbitrary units) | | | | | | **Fold change**  **Tx_10d_ injured /Vh_10d_ injured** | |
| --- | --- | --- | --- | --- | --- | --- | --- | --- | --- | --- | --- |
|  |  |  |  | **Vh_10d_ uninjured** | **Vh_10d_ injured** | **Vh_28d_ injured** | **Tx_10d_ uninjured** | **Tx_10d_ injured** | **Tx_28d_ injured** | **Log (2) Fold change** | **Adj. p-value** |
| 3 | | 10566144 | TRIM21 | 369.37 | 423.46 | 449.72 | 379.81 | 564.25 | 523.97 | 0.3962 | 0.0616 |
| 3 | | 10566333 | Trim12a | 278.42 | 345.51 | 350.97 | 292.13 | 493.57 | 430.04 | 0.5197 | 0.0399 |
| 3 | | 10566358 | Trim30a/Trim30d | 439.17 | 757.57 | 566.97 | 538.04 | 1653.60 | 925.52 | 1.1440 | 0.0065 |
| 3 | | 10566571 | Gvin1 (includes others) | 102.61 | 103.45 | 90.74 | 118.06 | 175.80 | 117.46 | 0.8203 | 0.0270 |
| 3 | | 10566574 | Gvin1 (includes others) | 270.39 | 300.14 | 321.41 | 297.67 | 440.83 | 329.40 | 0.5528 | 0.0604 |
| 3 | | 10566578 | Gvin1 (includes others) | 193.54 | 193.79 | 168.03 | 213.50 | 352.67 | 230.37 | 0.9371 | 0.0165 |
| 3 | | 10566583 | Gm8995 | 350.57 | 398.14 | 441.69 | 367.80 | 745.04 | 478.96 | 0.9243 | 0.0111 |
| 3 | | 10568024 | CORO1A | 173.93 | 293.34 | 340.45 | 191.96 | 425.24 | 450.55 | 0.5245 | 0.0179 |
| 3 | | 10568355 | PYCARD | 227.78 | 329.85 | 329.31 | 259.61 | 471.54 | 417.00 | 0.5046 | 0.0694 |
| 3 | | 10568873 | ADAM8 | 190.20 | 290.90 | 307.55 | 182.74 | 402.05 | 296.41 | 0.4613 | 0.0259 |
| 3 | | 10569017 | Ifitm3 | 4800.43 | 5143.77 | 5662.81 | 5622.61 | 6788.34 | 6943.22 | 0.4150 | 0.0390 |
| 3 | | 10569057 | RNH1 | 402.66 | 530.49 | 559.73 | 466.39 | 687.72 | 612.24 | 0.3728 | 0.0167 |
| 3 | | 10569102 | IRF7 | 134.91 | 132.45 | 118.54 | 134.16 | 267.68 | 139.53 | 1.0321 | 0.0011 |
| 3 | | 10571984 | DDX60 | 258.73 | 267.26 | 239.09 | 258.29 | 603.68 | 433.41 | 1.1925 | 0.0005 |
| 3 | | 10572212 | GMIP | 143.68 | 209.97 | 215.50 | 167.36 | 247.45 | 238.69 | 0.2321 | 0.0977 |
| 3 | | 10572897 | HMOX1 | 538.23 | 506.20 | 522.11 | 524.15 | 766.35 | 531.57 | 0.5884 | 0.0867 |
| 3 | | 10573583 | MAN2B1 | 468.58 | 1173.50 | 1163.80 | 545.94 | 1700.20 | 1421.66 | 0.5268 | 0.0671 |
| 3 | | 10574135 | NLRC5 | 45.02 | 48.96 | 53.79 | 49.18 | 79.53 | 66.31 | 0.6786 | 0.0137 |
| 3 | | 10574163 | NLRC5 | 160.49 | 189.79 | 203.61 | 183.28 | 277.57 | 270.14 | 0.5410 | 0.0259 |
| 3 | | 10574976 | PLA2G15 | 190.78 | 263.38 | 272.21 | 199.43 | 320.54 | 320.88 | 0.2799 | 0.0880 |
| 3 | | 10575799 | PLCG2 (includes EG:234779) | 185.50 | 262.17 | 258.40 | 213.54 | 346.37 | 306.99 | 0.4037 | 0.0534 |
| 3 | | 10576034 | IRF8 | 177.66 | 340.36 | 350.87 | 221.37 | 461.92 | 416.34 | 0.4380 | 0.0741 |
| 3 | | 10577792 | PLEKHA2 | 215.53 | 253.22 | 243.64 | 225.36 | 346.97 | 296.45 | 0.4689 | 0.0774 |
| 3 | | 10578493 | TLR3 | 277.32 | 259.04 | 283.19 | 282.99 | 365.97 | 337.17 | 0.4990 | 0.0456 |
| 3 | | 10579347 | IFI30 | 424.24 | 713.72 | 683.40 | 498.08 | 1153.01 | 797.79 | 0.6448 | 0.0760 |
| 3 | | 10579532 | BST2 | 499.72 | 671.82 | 542.11 | 622.85 | 1440.99 | 823.91 | 1.0991 | 0.0000 |
| 3 | | 10579636 | CYP4F2 | 73.51 | 124.67 | 121.09 | 83.59 | 161.98 | 135.05 | 0.3789 | 0.0607 |
| 3 | | 10581378 | PSMB10 | 389.19 | 465.45 | 449.10 | 416.83 | 603.70 | 486.55 | 0.3709 | 0.0233 |
| 3 | | 10581434 | DPEP2 | 76.78 | 91.31 | 106.20 | 85.14 | 127.20 | 111.02 | 0.4520 | 0.0791 |
| 3 | | 10582303 | CYBA | 259.25 | 709.60 | 678.55 | 292.65 | 1181.92 | 868.68 | 0.7194 | 0.0026 |
| 3 | | 10582868 | SP110 | 485.10 | 696.36 | 731.92 | 521.95 | 996.45 | 791.43 | 0.5095 | 0.0077 |
| 3 | | 10582874 | SP110 | 68.12 | 105.40 | 101.60 | 67.10 | 192.02 | 119.62 | 0.8663 | 0.0478 |
| 3 | | 10582879 | Csprs (includes others) | 107.77 | 116.48 | 119.05 | 111.55 | 159.59 | 128.17 | 0.4641 | 0.0320 |

| Cluster | **Affymetrix transcript #** | Gene Symbol | Average levels of expression (arbitrary units) | | | | | | **Fold change**  **Tx_10d_ injured /Vh_10d_ injured** | |
| --- | --- | --- | --- | --- | --- | --- | --- | --- | --- | --- |
|  |  |  | **Vh_10d_ uninjured** | **Vh_10d_ injured** | **Vh_28d_ injured** | **Tx_10d_ uninjured** | **Tx_10d_ injured** | **Tx_28d_ injured** | **Log (2) Fold change** | **Adj. p-value** |
| 3 | 10583100 | MMP8 | 54.15 | 98.76 | 66.88 | 54.48 | 160.85 | 101.97 | 0.6343 | 0.0557 |
| 3 | 10583112 | MMP27 | 124.91 | 322.65 | 248.42 | 142.51 | 474.12 | 354.89 | 0.5563 | 0.0954 |
| 3 | 10584334 | SIAE | 282.12 | 271.39 | 321.60 | 292.48 | 371.13 | 320.80 | 0.4543 | 0.0143 |
| 3 | 10585699 | FABP5 | 1497.15 | 2684.52 | 2872.79 | 1542.99 | 3770.88 | 3422.73 | 0.4834 | 0.0130 |
| 3 | 10585874 | HEXA | 661.04 | 1462.59 | 1312.96 | 700.50 | 1930.15 | 1440.23 | 0.3951 | 0.0712 |
| 3 | 10586604 | RPS27L | 1524.92 | 2018.41 | 1638.16 | 1892.30 | 2704.89 | 1899.06 | 0.4096 | 0.0753 |
| 3 | 10587350 | DDX43 | 35.19 | 37.21 | 38.58 | 39.85 | 45.82 | 44.05 | 0.2994 | 0.0084 |
| 3 | 10587503 | SH3BGRL2 | 129.15 | 162.91 | 188.73 | 158.54 | 228.63 | 235.23 | 0.4924 | 0.0701 |
| 3 | 10588037 | RBP1 | 504.23 | 626.85 | 542.83 | 628.04 | 953.92 | 709.04 | 0.6182 | 0.0645 |
| 3 | 10589884 | Bcl2a1c | 83.82 | 202.58 | 245.09 | 102.30 | 296.87 | 330.83 | 0.5353 | 0.0534 |
| 3 | 10590635 | CCR5 | 115.44 | 466.57 | 534.95 | 141.61 | 870.58 | 614.48 | 0.8825 | 0.0246 |
| 3 | 10593050 | IL10RA | 108.78 | 215.06 | 287.23 | 109.61 | 363.25 | 320.93 | 0.7554 | 0.0010 |
| 3 | 10593191 |  | 37.95 | 36.02 | 54.12 | 50.18 | 60.04 | 50.61 | 0.7170 | 0.0584 |
| 3 | 10594053 | PML | 219.37 | 233.75 | 222.36 | 237.19 | 275.54 | 242.22 | 0.2352 | 0.0791 |
| 3 | 10596637 | MAPKAPK3 | 162.33 | 259.98 | 269.91 | 182.38 | 367.86 | 306.49 | 0.5012 | 0.0297 |
| 3 | 10597461 | CMTM7 | 281.55 | 380.83 | 409.95 | 357.00 | 493.07 | 471.55 | 0.3616 | 0.0874 |
| 3 | 10598013 | CCR5 | 115.44 | 466.57 | 534.95 | 141.61 | 870.58 | 614.48 | 0.8825 | 0.0246 |
| 3 | 10598152 | BC147527 | 74.59 | 106.19 | 96.57 | 80.70 | 165.87 | 102.23 | 0.6251 | 0.0226 |
| 3 | 10598499 |  | 6446.91 | 6145.02 | 6836.39 | 6557.28 | 7256.03 | 6833.74 | 0.2411 | 0.0589 |
| 3 | 10599192 | LONRF3 | 251.83 | 401.51 | 419.49 | 255.12 | 571.70 | 471.80 | 0.5093 | 0.0257 |
| 3 | 10599627 | HPRT1 | 998.49 | 1098.36 | 1113.73 | 1065.84 | 1459.19 | 1185.61 | 0.4051 | 0.0340 |
| 3 | 10601385 | Tlr13 | 112.65 | 463.06 | 463.64 | 116.18 | 898.34 | 612.67 | 0.9670 | 0.0022 |
| 3 | 10601416 | P2RY10 | 24.62 | 29.77 | 33.28 | 27.67 | 44.40 | 35.33 | 0.5475 | 0.0265 |
| 3 | 10601581 | TRIM5 | 330.20 | 386.61 | 411.92 | 319.13 | 552.20 | 479.67 | 0.5181 | 0.0606 |
| 3 | 10603860 | CFP | 241.04 | 355.66 | 357.96 | 294.49 | 497.72 | 365.28 | 0.4862 | 0.0790 |
| 3 | 10605181 | RENBP | 125.61 | 266.27 | 268.11 | 161.21 | 394.99 | 341.13 | 0.5812 | 0.0590 |
| 3 | 10605303 | DNASE1L1 | 424.55 | 522.38 | 603.02 | 477.90 | 765.97 | 733.45 | 0.5416 | 0.0066 |
| 3 | 10605874 | EDA2R | 82.10 | 126.14 | 96.62 | 92.39 | 236.74 | 120.27 | 0.9179 | 0.0001 |
| 3 | 10606016 | IL2RG | 129.98 | 282.74 | 246.08 | 147.93 | 578.09 | 350.52 | 1.0711 | 0.0646 |
| 3 | 10606355 | CYSLTR1 | 131.10 | 254.48 | 290.26 | 191.64 | 439.56 | 301.79 | 0.7746 | 0.0617 |
| 3 | 10606694 | BTK | 89.48 | 187.87 | 209.92 | 109.45 | 276.41 | 249.13 | 0.5498 | 0.0762 |
| 3 | 10606714 | GLA | 332.73 | 934.57 | 756.74 | 351.04 | 1595.43 | 1062.94 | 0.7608 | 0.0047 |
| 3 | 10607868 | TLR8 | 72.18 | 181.21 | 181.63 | 84.12 | 266.44 | 233.25 | 0.5676 | 0.0222 |
| 3 | 10607870 | TLR7 | 65.45 | 245.60 | 209.81 | 92.81 | 487.42 | 251.80 | 0.9962 | 0.0001 |
| 3 | 10608646 |  | 181.23 | 192.44 | 194.07 | 220.48 | 264.29 | 224.51 | 0.4728 | 0.0392 |
| 3 | 10608648 |  | 33.34 | 41.96 | 35.23 | 37.69 | 58.57 | 42.42 | 0.4666 | 0.0559 |
| 3 | 10608650 |  | 321.98 | 432.15 | 460.08 | 477.93 | 683.44 | 563.22 | 0.6765 | 0.0173 |
| 3 | 10608652 |  | 57.47 | 62.78 | 72.43 | 58.40 | 83.34 | 66.87 | 0.3899 | 0.0641 |

| Cluster | **Affymetrix transcript #** | Gene Symbol | Average levels of expression (arbitrary units) | | | | | | **Fold change**  **Tx_10d_ injured /Vh_10d_ injured** | |
| --- | --- | --- | --- | --- | --- | --- | --- | --- | --- | --- |
|  |  |  | **Vh_10d_ uninjured** | **Vh_10d_ injured** | **Vh_28d_ injured** | **Tx_10d_ uninjured** | **Tx_10d_ injured** | **Tx_28d_ injured** | **Log (2) Fold change** | **Adj. p-value** |
| 4 | 10343311 |  | 323.32 | 228.08 | 309.84 | 301.61 | 381.05 | 312.52 | 0.7249 | 0.0536 |
| 4 | 10345423 | PLEKHB2 | 272.22 | 227.19 | 235.74 | 223.85 | 278.61 | 239.55 | 0.2973 | 0.0399 |
| 4 | 10360684 | EPHX1 | 609.14 | 396.81 | 559.47 | 697.18 | 714.84 | 699.40 | 0.8212 | 0.0811 |
| 4 | 10362896 | Cd24a | 3055.43 | 1644.70 | 2694.10 | 2522.15 | 2446.69 | 1830.07 | 0.5835 | 0.0019 |
| 4 | 10372807 | MSRB3 | 375.02 | 252.37 | 281.50 | 373.36 | 326.55 | 264.51 | 0.3814 | 0.0359 |
| 4 | 10378068 | XAF1 | 742.43 | 525.38 | 580.80 | 666.76 | 802.68 | 622.39 | 0.6224 | 0.0207 |
| 4 | 10383168 | RNF213 | 788.22 | 569.09 | 606.44 | 693.32 | 858.66 | 671.31 | 0.5803 | 0.0252 |
| 4 | 10383196 | RNF213 | 137.54 | 112.72 | 114.63 | 122.71 | 154.54 | 134.60 | 0.4608 | 0.0339 |
| 4 | 10383200 | RNF213 | 313.59 | 246.24 | 221.67 | 336.12 | 436.28 | 288.75 | 0.8041 | 0.0631 |
| 4 | 10383202 | RNF213 | 751.93 | 522.78 | 529.47 | 783.68 | 930.37 | 624.94 | 0.8117 | 0.0413 |
| 4 | 10383204 | RNF213 | 562.48 | 385.59 | 409.79 | 525.31 | 716.11 | 483.09 | 0.8826 | 0.0314 |
| 4 | 10383212 | RNF213 | 135.94 | 111.65 | 138.04 | 133.53 | 153.36 | 137.70 | 0.4575 | 0.0670 |
| 4 | 10383214 | RNF213 | 595.73 | 441.07 | 458.03 | 565.28 | 663.43 | 548.08 | 0.5698 | 0.0119 |
| 4 | 10385533 | Tgtp1/Tgtp2 | 582.10 | 435.28 | 421.83 | 611.05 | 800.32 | 591.01 | 0.8798 | 0.0398 |
| 4 | 10394843 |  | 231.28 | 183.64 | 207.67 | 210.07 | 232.44 | 198.96 | 0.3342 | 0.0912 |
| 4 | 10395039 | CMPK2 | 81.65 | 74.29 | 77.61 | 85.76 | 98.06 | 83.78 | 0.3882 | 0.0392 |
| 4 | 10399710 | RSAD2 | 178.94 | 118.15 | 116.84 | 192.27 | 297.81 | 202.55 | 1.2647 | 0.0044 |
| 4 | 10408600 | SERPINB6 | 1171.77 | 1056.38 | 1234.90 | 1308.23 | 1439.77 | 1389.06 | 0.4463 | 0.0023 |
| 4 | 10412657 |  | 112.03 | 108.41 | 143.78 | 167.44 | 177.12 | 168.79 | 0.6492 | 0.0759 |
| 4 | 10429564 | Ly6a (includes others) | 1444.14 | 1305.21 | 1156.80 | 2307.32 | 2335.12 | 1399.50 | 0.9668 | 0.0740 |
| 4 | 10429568 | Ly6a (includes others) | 1974.17 | 1124.19 | 1027.71 | 2073.81 | 1885.82 | 1151.41 | 0.8504 | 0.0548 |
| 4 | 10429573 | Ly6a (includes others) | 1186.88 | 745.19 | 691.22 | 1295.18 | 1158.57 | 701.98 | 0.6945 | 0.0582 |
| 4 | 10445293 | PLA2G7 | 1396.25 | 820.89 | 1153.59 | 1074.59 | 1351.09 | 1114.92 | 0.7225 | 0.0144 |
| 4 | 10459288 | ADRB2 | 246.32 | 210.30 | 244.38 | 323.53 | 275.29 | 230.28 | 0.3964 | 0.0740 |
| 4 | 10459467 |  | 69.03 | 61.94 | 64.30 | 71.66 | 73.23 | 68.52 | 0.2399 | 0.0791 |
| 4 | 10460392 | POLD4 | 410.92 | 387.68 | 422.57 | 507.68 | 529.25 | 470.75 | 0.4470 | 0.0665 |
| 4 | 10462603 | FAS | 279.42 | 236.73 | 222.20 | 358.17 | 342.62 | 220.59 | 0.5661 | 0.0761 |
| 4 | 10462613 | IFIT2 | 730.47 | 468.43 | 577.79 | 695.48 | 777.82 | 698.41 | 0.7192 | 0.0098 |
| 4 | 10462621 | I830012O16Rik/Ifit3 | 230.59 | 96.26 | 147.28 | 214.90 | 364.19 | 293.86 | 1.9320 | 0.0003 |
| 4 | 10462623 | IFIT1B | 334.71 | 189.09 | 211.41 | 401.66 | 635.52 | 391.94 | 1.8121 | 0.0004 |
| 4 | 10465826 | C11orf83 | 429.58 | 382.55 | 462.41 | 530.69 | 501.41 | 450.18 | 0.3833 | 0.0222 |
| 4 | 10473384 | SLC43A3 | 1592.69 | 926.20 | 909.54 | 1304.14 | 1247.57 | 1499.55 | 0.4423 | 0.0459 |
| 4 | 10490818 | STMN2 | 382.44 | 191.16 | 267.36 | 412.71 | 300.78 | 258.58 | 0.6595 | 0.0876 |
| 4 | 10496539 | GBP5 | 189.46 | 137.58 | 147.46 | 203.95 | 211.11 | 199.15 | 0.6196 | 0.0434 |
| 4 | 10496813 | CTBS | 402.19 | 314.43 | 359.33 | 379.92 | 387.32 | 359.12 | 0.3030 | 0.0537 |

| Cluster | **Affymetrix transcript #** | Gene Symbol | Average levels of expression (arbitrary units) | | | | | | **Fold change**  **Tx_10d_ injured /Vh_10d_ injured** | |
| --- | --- | --- | --- | --- | --- | --- | --- | --- | --- | --- |
|  |  |  | **Vh_10d_ uninjured** | **Vh_10d_ injured** | **Vh_28d_ injured** | **Tx_10d_ uninjured** | **Tx_10d_ injured** | **Tx_28d_ injured** | **Log (2) Fold change** | **Adj. p-value** |
| 4 | 10514388 |  | 16.59 | 14.37 | 16.33 | 18.70 | 17.97 | 16.63 | 0.3198 | 0.0143 |
| 4 | 10518455 | AGTRAP | 327.28 | 273.68 | 301.58 | 275.60 | 373.67 | 309.72 | 0.4479 | 0.0580 |
| 4 | 10523451 | ANXA3 | 2724.92 | 1874.97 | 2033.35 | 2817.73 | 2670.65 | 2566.67 | 0.5113 | 0.0067 |
| 4 | 10537227 | C7orf49 | 910.69 | 646.94 | 856.95 | 1111.19 | 906.05 | 997.34 | 0.4925 | 0.0249 |
| 4 | 10541910 | VWF | 525.04 | 391.60 | 476.09 | 551.23 | 495.58 | 454.66 | 0.3426 | 0.0564 |
| 4 | 10542880 | METTL20 | 527.15 | 251.28 | 280.54 | 529.13 | 379.77 | 274.45 | 0.6118 | 0.0108 |
| 4 | 10542885 | C12orf35 | 720.62 | 634.04 | 588.52 | 658.26 | 812.64 | 707.30 | 0.3603 | 0.0398 |
| 4 | 10542911 | SAMD9L | 1348.08 | 1094.16 | 1159.25 | 1265.81 | 1661.29 | 1290.39 | 0.6081 | 0.0349 |
| 4 | 10569962 | CCL25 | 333.45 | 253.41 | 260.53 | 385.63 | 360.41 | 258.39 | 0.5168 | 0.0333 |
| 4 | 10570434 | IFITM1 | 150.09 | 145.55 | 157.01 | 213.83 | 195.75 | 183.95 | 0.4267 | 0.0792 |
| 4 | 10578448 | CYP4V2 | 359.79 | 269.59 | 356.95 | 362.11 | 340.12 | 375.98 | 0.3372 | 0.0365 |
| 4 | 10585194 | IL18 (includes EG:16173) | 106.53 | 84.81 | 105.23 | 127.72 | 138.23 | 111.65 | 0.7046 | 0.0317 |
| 4 | 10585842 | Nptn | 2021.83 | 1601.49 | 1792.26 | 2117.84 | 1838.84 | 1799.94 | 0.1990 | 0.0805 |
| 4 | 10603182 | ARHGAP6 | 97.12 | 92.17 | 106.44 | 117.99 | 121.55 | 109.61 | 0.3983 | 0.0646 |
| 4 | 10603573 | SYTL5 | 76.95 | 70.16 | 75.64 | 98.05 | 114.36 | 72.89 | 0.7020 | 0.0144 |
