# Supplementary material for: Axonal neuregulin 1 is a rate limiting but not essential factor for nerve remyelination
Source: Brain. 2013 Jun 24;136(7):2279–97. doi: 10.1093/brain/awt148 (PMC3692042; doi:10.1093/brain/awt148)
Supplement: Supplementary Data [file supp_awt148_brain-2012-02167-File018.doc]

**Supplemental Table 1.Transcripts differentially expressed between Tx and Vh-treated animals in the injured sciatic nerve 10d post injury.**

Levels of expression correspond to hybridisation intensities after normalisation, background subtraction and summarisation of individual probe sets to the “transcript cluster level”, and are indicated as averages for each of the experimental groups considered. Log 2 Fold change is also indicated as well as respective adjusted p-value (only transcripts with FDR p-value <0.1 are indicated).

**Supplemental Figure 1. Models of nerve injury**

Schematic diagrams of models of peripheral nerve injury used in this study. ***A*** Sciatic nerve crush, the sciatic nerve is crushed with forceps and tissue is harvested for analysis either 10 days, 28 days, or 2 months post injury. ***B*** Sciatic nerve transection with immediate renanastomosis. The sciatic nerve transected and then immediately repaired with fibrin glue. Tissue is harvested for analysis at, 2 months or 3 months post injury. In the case of assessment of SC proliferation post sciatic nerve transection, no repair was carried out, and animals received BrdU injections once a day for the 3 days before the tissue was harvested at day 4 post transection. ***C*** Delayed reinnervation model. The tibial nerve is double ligated and then transected close to the branching site. The tibial nerve is left to denervate for 2 weeks. Then the common peroneal nerve is ligated, transected above the ligature and the proximal end of the common peroneal is glued into the freshly trimmed distal stump of the tibial. The animals are left for 10 days for axons to grow into the tibial nerve. Animals are injected with BrdU for 3 days before tissue is harvested. As a control, a cohort of animals underwent the initial tibial nerve transection, at 2 weeks the common peroneal was double ligated but the common peroneal was not glued to the tibial, therefore no repair took place.

**Supplemental Figure 2.Tamoxifen efficiently induces Cre recombinase activity in Cre-ERTM;R26R reporter mice.** Following Tx treatment but not Vh treatment, β-galactosidase activity can be detected using the X-gal reaction resulting in a blue product in the sciatic nerve, the motoneurons of the lumbar spinal cord and the lumbar dorsal root ganglion (DRG). Blue staining was not seen in tissue from Vh treated animals. Vh – vehicle, Tx – tamoxifen Scale bars 20μm.

**Supplemental Figure 3.Nrg1 signalling is dispensable for peripheral nerve maintenance in adulthood.**

***A*** Electron micrographs of transverse sections of the sciatic nerve of Vh control and conNrg1 mutant mice 12 weeks post dosing. Scale bars 2μm. ***B*** Total axon numbers per sciatic nerve section. ***C*** % of axons myelinated per sciatic nerve section n = 4. ***D*** G-ratio frequency distribution and ***E*** frequency distribution of the number of unmyelinated axons associated with each SC in Vh control and conNrg1 mutant mouse sciatic nerves 12 weeks post dosing, n = 4. ***F*** photomicrographs of intraepidermal nerve fibres (IENF) labelled with pan-neuronal marker PGP9.5, individual fibres marked by arrows. ***G***IENF analysis n = 5-8. ***H*** photomicrographs of neuromuscular junctions (NMJs) labelled for: myelin using myelin basic protein (MBP, blue), the presynaptic component and axons using synaptic vesicle marker SV2 and neurofilament marker NF (green) and the post synaptic component α-bungarotoxin (red). Normal NMJ morphology is seen in uninjured Vh control and conNrg1 mutant animals ***I*** Quantification of the number of NMJs that are innervated 12 weeks following dosing n = 3.

**Supplemental Figure 4.Sciatic nerve transection**

***A*** Electronmicrograph of transverse section of sciatic nerve from a conNrg1 mutant animal distal to transection site 2 months post injury. Although some remyelination has occurred large diameter axons are still unmyelinated. Scale bar 2µm. ***B*** SFI over 3 months following injury, no significant functional recovery was seen in any group n=6-9.***C*** Photomicrographs of NMJs in conNrg1 mutant mice 3months post sciatic nerve transection, the gastrocnemius labelled for: MBP (blue), SV2 and neurofilament marker NF(green) and α-bungarotoxin (red). Extensive remyelination has occurred, although segments of axons remain unmyelinated (arrows). Scale bar 20µm. ***D*** Photomicrographs of the longitudinal spinal cord sections taken from the animals that underwent sciatic nerve transection and were followed for 3 months after the injury. Pictures are of the lumbar level of the cord from the Vhcontrol and conNrg1 mutant mice, motoneurons are identified by their characteristic morphology. Nrg1 labelling can be seen as punctate staining in Control and Vh spinal cords but is no longer present in conNrg1 mutants. Scale bar 50µm.

**Supplemental Figure 5. Changes in expression of α and β isoforms of Nrg1 following sciatic nerve crush in wild type animals.**

Quantitative real time PCR with primers designed against either Nrg1 αEGF domain ***A***, or Nrg1 βEGF domain ***B***, relative to GAPDH expression in sciatic nerves from C57Bl/6 mice that were uninjured or 1 or 10days post sciatic nerve crush. There is negligible change in the expression of β isoforms whereas α isoforms are upregulated by 1 day post injury and are still high at 10 days post injury. One Way ANOVA post hoc Tukey n = 8.

**Supplemental Figure 6. 3 months post sciatic nerve crush Nrg1 is still absent in conNrg1 mutant mice nerves.**

Western blot showing the absence of either the FL Nrg1 form or the CTF 3 months post injury in conNrg1 mutants compared to Vh control.
